# Identification and Optimization of Novel Inhibitors of the Polyketide Synthase 13 Thioesterase Domain with Antitubercular Activity

**DOI:** 10.1021/acs.jmedchem.3c01514

**Published:** 2023-11-10

**Authors:** Simon R. Green, Caroline Wilson, Thomas C. Eadsforth, Avinash S. Punekar, Fabio K. Tamaki, Gavin Wood, Nicola Caldwell, Barbara Forte, Neil R. Norcross, Michael Kiczun, John M. Post, Eva Maria Lopez-Román, Curtis A. Engelhart, Iva Lukac, Fabio Zuccotto, Ola Epemolu, Helena I. M. Boshoff, Dirk Schnappinger, Chris Walpole, Ian H. Gilbert, Kevin D. Read, Paul G. Wyatt, Beatriz Baragaña

**Affiliations:** ‡ Drug Discovery Unit, Division of Biological Chemistry and Drug Discovery, School of Life Sciences, University of Dundee, Dundee DD1 5EH, U.K.; † Department of Microbiology and Immunology, Weill Cornell Medical College, New York, New York 10065, United States; § Global Health Medicines R&D, GlaxoSmithKline, Severo Ochoa 2, Tres Cantos, 28760 Madrid Spain; ∥ Tuberculosis Research Section, Laboratory of Clinical Immunology and Microbiology, NIAID, National Institutes of Health, 9000 Rockville Pike, Bethesda, Maryland 20892, United States; ⊥ Structural Genomics Consortium, Research Institute of the McGill University Health Centre, 1001 Boulevard Décarie, Site Glen Block E, ES1.1614, Montréal, QC H4A 3J1, Canada

## Abstract

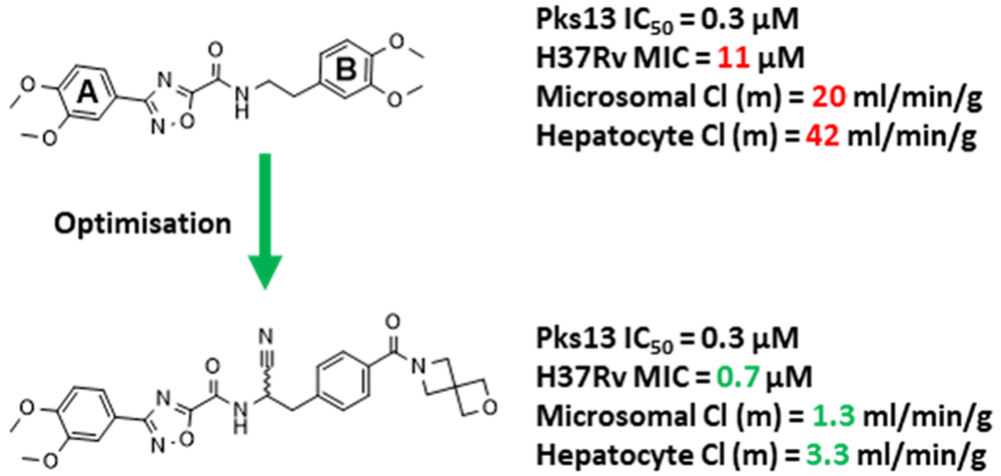

There is an urgent need for new tuberculosis (TB) treatments, with novel modes of action, to reduce the incidence/mortality of TB and to combat resistance to current treatments. Through both chemical and genetic methodologies, polyketide synthase 13 (Pks13) has been validated as essential for mycobacterial survival and as an attractive target for *Mycobacterium tuberculosis* growth inhibitors. A benzofuran series of inhibitors that targeted the Pks13 thioesterase domain, failed to progress to preclinical development due to concerns over cardiotoxicity. Herein, we report the identification of a novel oxadiazole series of Pks13 inhibitors, derived from a high-throughput screening hit and structure-guided optimization. This new series binds in the Pks13 thioesterase domain, with a distinct binding mode compared to the benzofuran series. Through iterative rounds of design, assisted by structural information, lead compounds were identified with improved antitubercular potencies (MIC < 1 μM) and *in vitro* ADMET profiles.

## Introduction

Prior to the Covid-19 pandemic, tuberculosis (TB) was the world’s leading infectious disease killer, resulting in 1.4 million deaths in 2019.^1^ The extent of the disease led the World Health Organization to initiate The End TB Strategy in 2015, with the stated goals that by 2035 there would be a 95% reduction in TB deaths and a 90% reduction in the incidence of TB.^2^ Although progress was being made toward these goals, the Covid-19 pandemic has resulted in significant set-backs,^3,4^ with the incidence of TB rising by 3.6% between 2020 and 2021, reversing declines of about 2% per year for most of the past two decades.^5^ In addition, the number of annual deaths has also increased during this period, with 1.6 million TB-related deaths in 2021.^5^ The burden of drug-resistant TB also increased during the pandemic,^5^ and there are already reports of resistance toward the newest TB drugs, bedaquiline and linezolid.^6,7^ As such, there is an ever-increasing need for new TB therapeutics and, in particular, agents against clinically novel targets where there would be no anticipated pre-existing clinical resistance.

Polyketide synthase 13 (Pks13: Rv3800c) was highlighted as a novel target for *Mycobacterium tuberculosis* growth inhibitors, as a result of the discovery of two phenotypic screening hits where resistant mutants indicated involvement of Pks13.^8−10^ Pks13 is essential for mycobacterial survival^11−13^ and is responsible for the last stage of mycolic acid synthesis.^11,14^ These long-chain fatty acids are a characteristic of the cell wall from the genus *Mycobacterium* and are known to be critical for the pathogenicity, virulence, and survival of *M. tuberculosis*.^15^ Pks13 is a multidomain protein, containing an acyl transferase (AT) domain, a ketosynthase (KS) domain, acyl carrier protein (ACP) domains, and a thioesterase (TE) domain.^11,16^ Mutants that were shown to be resistant to a benzofuran phenotypic screening hit were confirmed to contain single amino acid changes within the TE domain (D1607N and D1644G),^9^ while mutants resistant to a thiophene hit were found in the ACP domain.^8^ The original benzofuran hit was progressed to an early lead with the identification of TAM16, which demonstrated excellent *in vivo* efficacy in both acute and chronic murine models of *M. tuberculosis* infection.^17^ Further lead optimization on the benzofuran core, toward identification of a preclinical candidate, highlighted an off-target human ether-à-go-go-related gene (hERG) liability that could not be eliminated.^18^ This liability ultimately resulted in the termination of the development of the benzofuran series, due to concerns over potential cardiotoxicity.

Although originally identified as a phenotypic hit, lead optimization of the benzofuran series had been pursued as a structure-based drug discovery program, as both an *in vitro* Pks13 TE enzyme assay and a crystal structure were available.^17^ Given the impressive *in vivo* activity of TAM16, Pks13 is considered an attractive target for the discovery of new antitubercular agents. Since the benzofuran series was terminated because of a liability associated with its pharmacophore, a lipophilic basic amine, it was decided to pursue an *in vitro* screening program to identify novel chemical starting points against Pks13, devoid of this cardiotoxicity flag. This report describes the screening program and the optimization of the most promising hit, based on a novel oxadiazole core.

## Chemistry

### Synthetic Routes

**Scheme 1:**
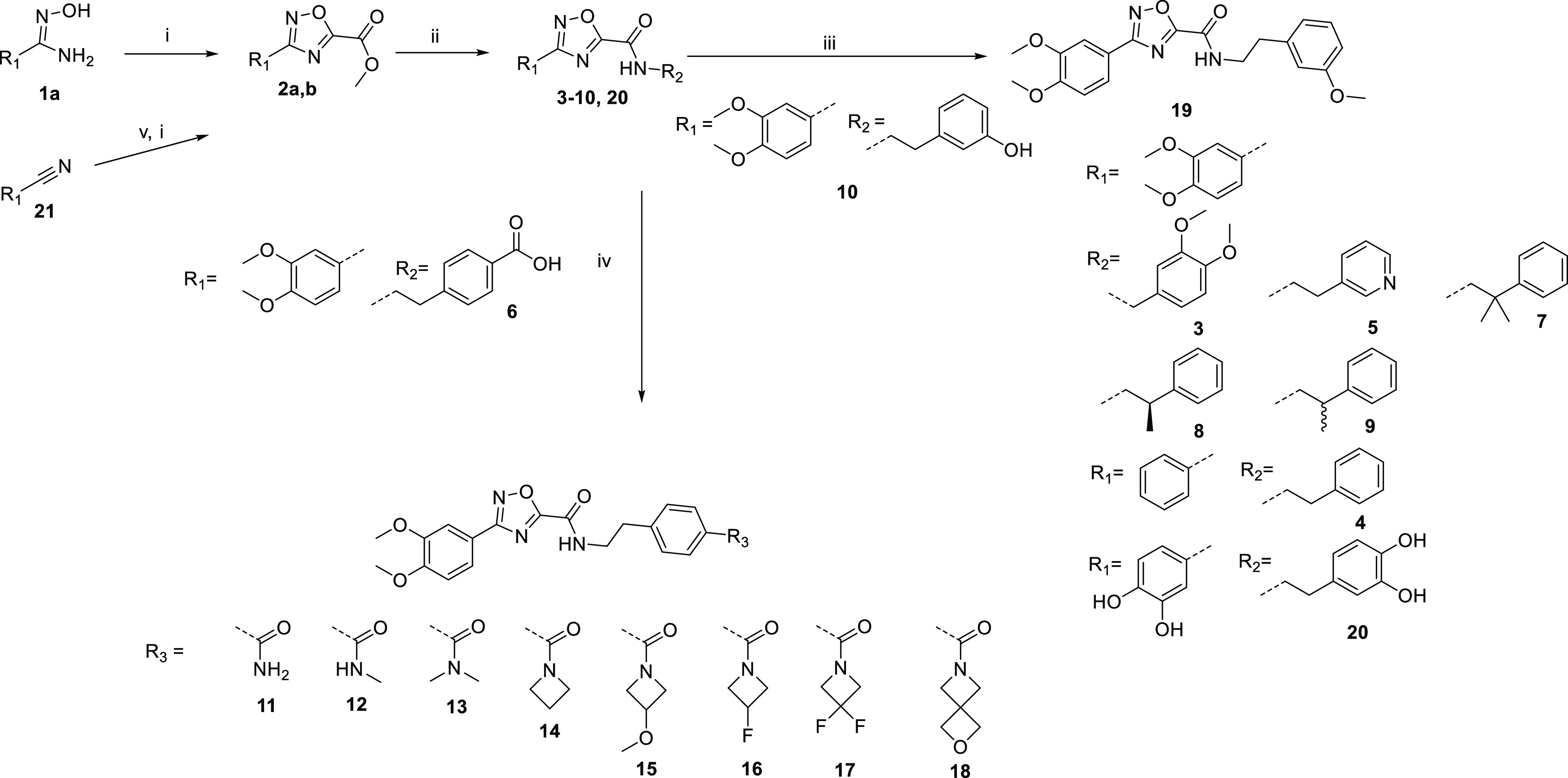
General Route to 1,2,4-Oxadiazole Carboxamides *Reagents and conditions*: (i) triethylamine, methyl 2-chloro-2-oxo-acetate, DCM, 0–40 °C; (ii) triethylamine, amine, MeOH, 60 °C; (iii) methyl iodide, K_2_CO_3_, DMF, rt; (iv) T3P, amine, triethylamine, DMF, rt, or HATU, amine, triethylamine, DMF, 0 °C to rt, or ammonia HOBt, EDCI HCl, DIPEA, THF, rt; (v) hydroxylamine hydrochloride, DIPEA, ethanol, 80 °C.

To explore the structure–activity relationship (SAR) of the phenethyl amide, the synthesis of several analogues was achieved using the route outlined in Scheme 1. The hydroxy amidine **1a** was condensed with methyl 2-chloro-2-oxo-acetate to afford the substituted oxadiazole methyl ester **2a**. The amides **3**–**10** were formed directly from the methyl ester of **2a**. The carboxylic acid **6** was converted to amides **11**–**18**. The *m*-phenol of **10** was alkylated to form the *m*-methoxyphenyl **19**. Compound **20** was synthesized from 3,4-dihydroxybenzonitrile **21**—it was converted to crude hydroxy amidine, which was condensed with methyl 2-chloro-2-oxo-acetate to give the 1,2,4-oxadiazole **2b**, and then the methyl ester of **2b** was converted to the amide **20**.

**Scheme 2:**
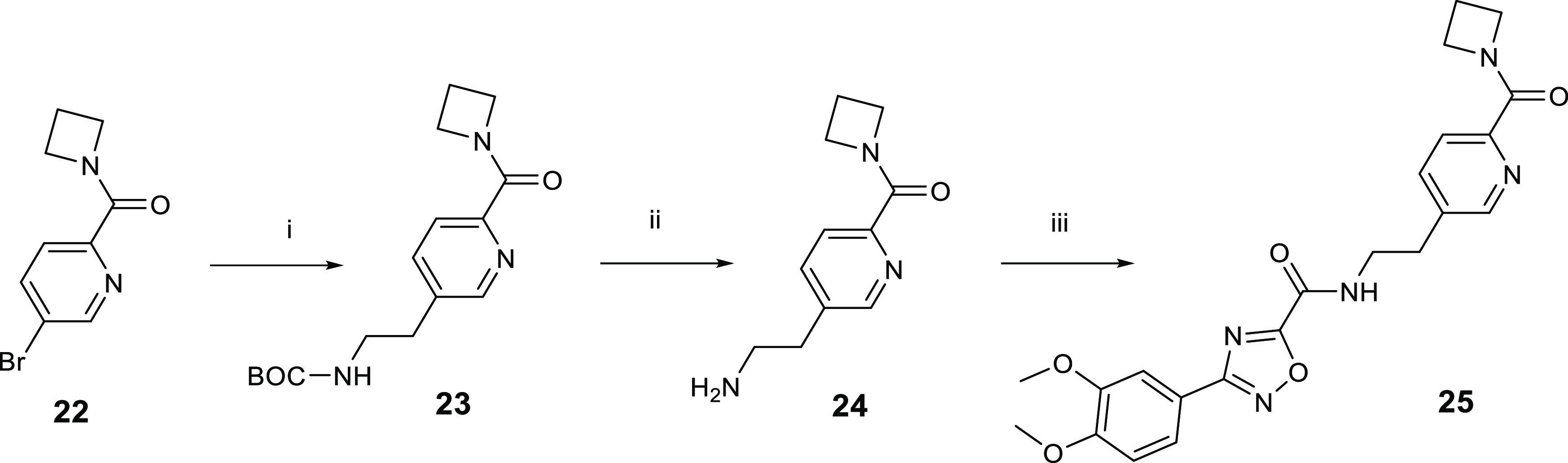
Route to Introduce a Substituted Pyridyl *Reagents and conditions*: (i) potassium (2-((*tert*-butoxycarbonyl)amino)ethyl)trifluoroborate, Pd(dppf)Cl_2_, Cs_2_CO_3_, toluene (3 mL), and H_2_O, 25–100 °C; (ii) TFA/DCM, 0–25 °C; (iii) ethyl 3-(3,4-dimethoxyphenyl)-1,2,4-oxadiazole-5-carboxylate, triethylamine, MeOH, 60 °C.

A route used to introduce a substituted pyridyl is outlined in Scheme 2. A palladium-catalyzed reaction with bromide **22** and potassium (2-((*tert*-butoxycarbonyl)amino)ethyl)trifluoroborate gave the Boc-protected amine **23**. The Boc protecting group was removed to give the amine **24**. The amine of **24** was then converted to the amide **25** by reaction with ethyl 3-(3,4-dimethoxyphenyl)-1,2,4-oxadiazole-5-carboxylate.

**Scheme 3:**
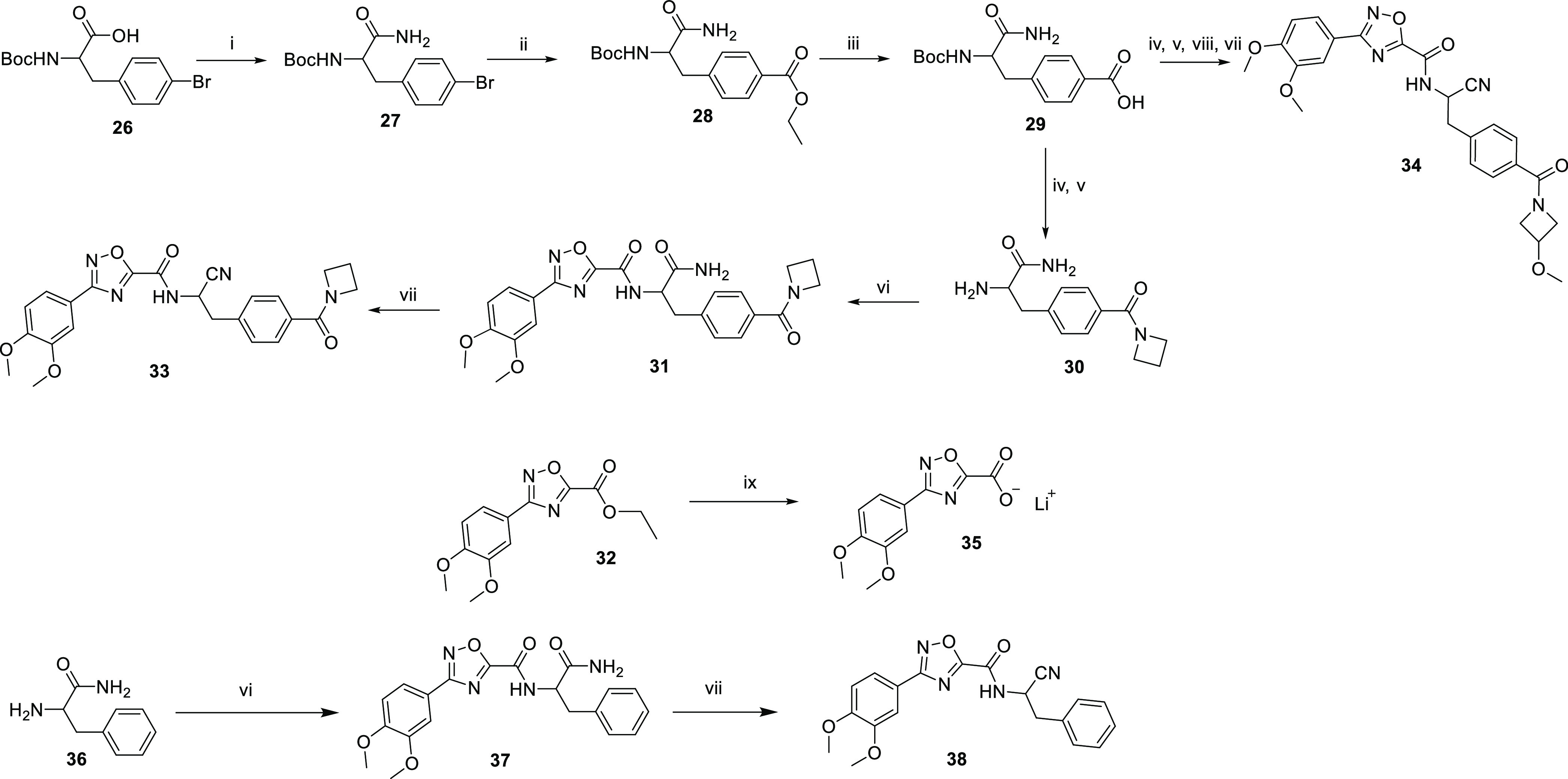
Route to Linker Chain Substituted with Nitrile *Reagents and conditions*: (i) HATU, DIPEA, NH_4_Cl, DCM, 25 °C; (ii) CO, Pd(dppf)Cl_2_, triethylamine, EtOH, 80 °C; (iii) LiOH, EtOH, water, 25 °C; (iv) HATU, DIPEA, amine, DCM or DMF, 20−25 °C; (v) TFA, DCM, 20–25 °C; (vi) ethyl 3-(3,4-dimethoxyphenyl)-1,2,4-oxadiazole-5-carboxylate, triethylamine, MeOH, 60 °C; (vii) triethylamine, TFAA, THF, N_2_, 0–25 °C or 0–20 °C; (viii) lithium 3-(3,4-dimethoxyphenyl)-1,2,4-oxadiazole-5-carboxylic acid, HATU, DIPEA, DMF 0–20 °C; (ix) LiOH·H_2_O, MeOH, water, 50 °C.

A route used to introduce a nitrile on the linker chain is outlined in Scheme 3. The carboxylic acid **26** was converted to the primary amide **27**. A palladium-catalyzed carbonylation reaction^19^ on the bromide of **27** gave the ethyl ester **28**. Carboxylic acid **29** was formed from the hydrolysis of ester **28**; the carboxylic acid **29** was then converted to the amide, and the Boc protecting group was removed from the amine to give **30**. The amide **31** was formed by reaction of the ester of **32** with the amine **30**. The primary amide of **31** was converted to the nitrile **33** by treatment with TFAA, triethylamine in THF. A similar route was used to make **34**: the carboxylic acid **29** was converted to the amide, and after Boc deprotection of the amine, the amide was yielded from the coupling with the lithium salt of the carboxylic acid **35**, which was formed from the hydrolysis of ester **32**, and finally the primary amide was converted to the nitrile **34**. A similar route was also used to make **38**: the amide **37** was formed by reaction of the ester of **32** with the amine **36**, and then the primary amide of **37** was converted to the nitrile **38** by treatment with TFAA, triethylamine in THF.

**Scheme 4:**
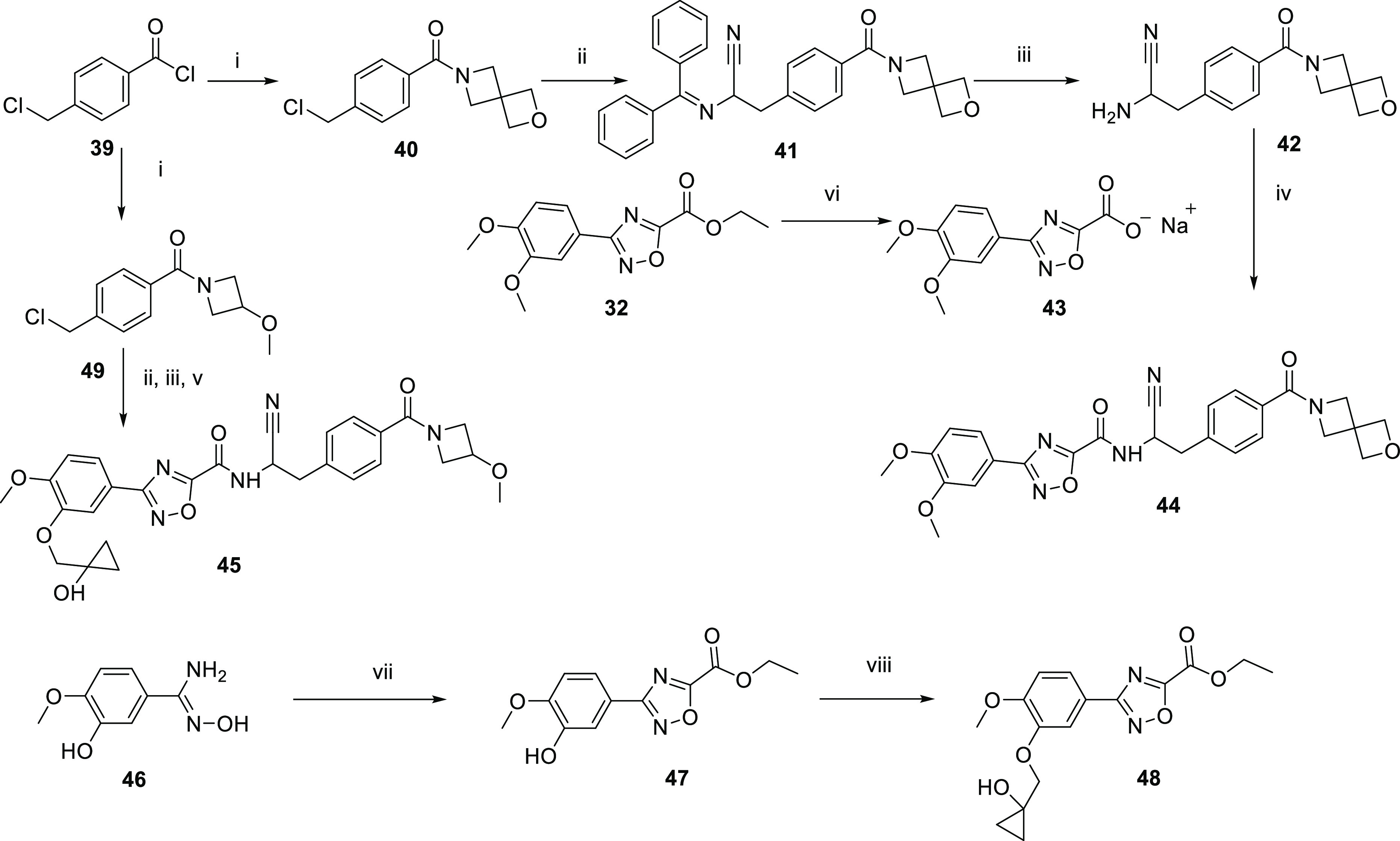
Alternative Route to Linker Chain Substituted with Nitrile *Reagents and conditions*: (i) amine, triethylamine, DCM, 0 °C, or DMAP, triethylamine, amine, N_2_ 0 °C to rt; (ii) 2-(benzhydrylideneamino)acetonitrile, NaOH, benzyltriethylammonium chloride, DCM, rt or 2-(benzhydrylideneamino)acetonitrile, NaOH, THF, 20 °C; (iii) HCl, water, dioxane, 20 °C or HCl, THF rt; (iv) sodium 3-(3,4-dimethoxyphenyl)-1,2,4-oxadiazole-5-carboxylate, HATU, DIPEA, DMF, N_2_, rt; (v) ethyl 3-[3-[(1-hydroxycyclopropyl)methoxy]-4- methoxy-phenyl]-1,2,4-oxadiazole-5-carboxylate, triethylamine, MeOH, 40 °C; (vi) NaOH, EtOH, rt; (vii) ethyl 2-chloro-2-oxo-acetate, DIPEA, THF, 0–80 °C; (viii) 1-tetrahydropyran-2-yloxycyclopropyl)methanol, PPh_3_, DEAD, THF, 0–25 °C.

An alternative route to introduce a nitrile on the linker chain is outlined in Scheme 4. The acid chloride **39** was converted to the amide **40**. 2-(Benzhydrylideneamino)acetonitrile was then alkylated with the chloride **40** to give the imine **41**.^20^ Hydrolysis of the imine **41** yielded the aminonitrile **42**. The sodium salt of the carboxylic acid **43** was formed from the hydrolysis of the ester **32**. Amide formation^21^ was achieved by reaction of the sodium salt of the carboxylic acid **43** with aminonitrile **42**, HATU, and DIPEA in DMF to afford **44**. To synthesize **45**, the hydroxy amidine **46** was condensed with ethyl 2-chloro-2-oxo-acetate to afford **47**. **48** was formed by a Mitsunobu^22^ reaction on the phenol of **47**. The acid chloride **39** was converted to the amide **49**. 2-(Benzhydrylideneamino)acetonitrile was then alkylated with the chloride **49** to give the imine, and hydrolysis of the imine yielded the aminonitrile. The amide **45** was formed by reaction of the aminonitrile with the ethyl ester **48**. The route in Scheme 3 introduced the nitrile at the start of the synthesis and was shorter than the route in Scheme 2. This route was used to make **44** at a higher scale (410 mg) and high levels of purity (>98%, no impurity >0.5%) for *in vivo* studies.

**Scheme 5:**
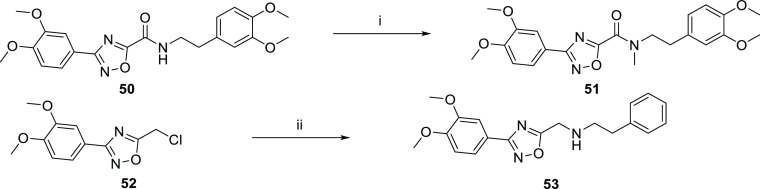
Routes to Amide Modifications *Reagents and conditions*: (i) NaH, methyl iodide, DMF rt; (ii) 2-phenylethanamine, triethylamine, DCM, 40 °C.

Routes to amide modifications are outlined in Scheme 5. The amide of the commercial compound **50** was alkylated with methyl iodide to give the methyl amide **51**. Alkylation of 2-phenylethanamine with the chloride **52** gave the amine **53**.

**Scheme 6:**
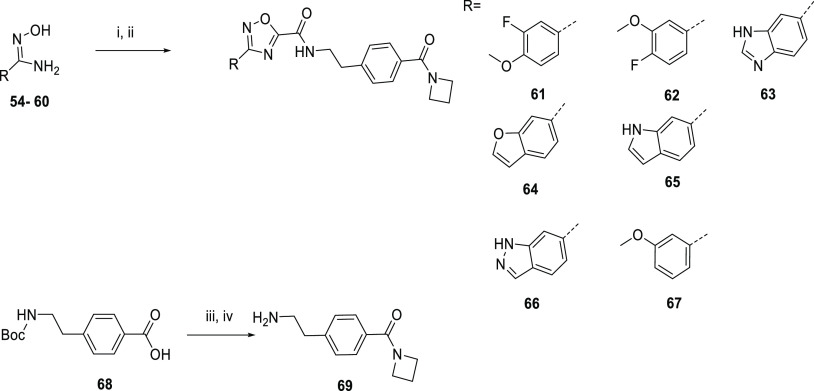
Route to Dimethoxyphenyl Ring Modifications *Reagents and conditions*: (i) ethyl 2-chloro-2-oxo-acetate, DIPEA, THF, 0–80 °C; (ii) [4-(2-aminoethyl)phenyl](azetidin-1-yl), triethylamine, MeOH, 60 °C; (iii) HATU, DIPEA, amine, DCM, 25 °C; (iv) TFA/DCM 25 °C.

A route to explore dimethoxyphenyl ring modifications is outlined in Scheme 6. The hydroxy amidines **54**–**60** were condensed with ethyl 2-chloro-2-oxo-acetate to afford substituted oxadiazole ethyl esters, and the amides **61**–**67** were formed by reaction of the ethyl esters with amine **69**. **69** was formed by converting the carboxylic acid of **68** to the amide, and then the Boc protecting group was removed from the amine group.

**Scheme 7:**
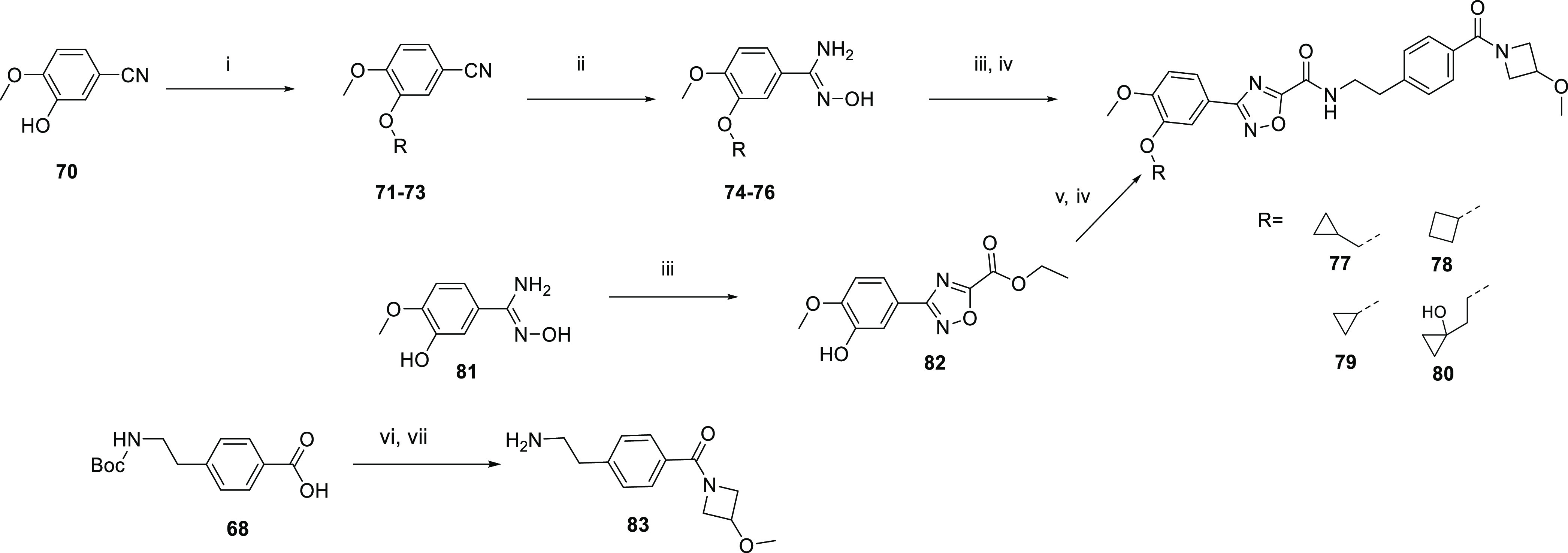
Routes to Methoxy Modifications *Reagents and conditions*: (i) bromide, K_2_CO_3_, DMF, 40–60 °C, or bromide, KI, CsCO_3_, DMSO, 140 °C; (ii) hydroxylamine hydrochloride, DIPEA, EtOH, 70–80 °C; (iii) ethyl 2-chloro-2-oxo-acetate, DIPEA, THF, 0–60 °C; (iv) 4-(2-aminoethyl)phenyl]-(3-methoxyazetidin-1-yl)methanone hydrochloride, triethylamine, MeOH, 60–70 °C; (v) 1-tetrahydropyran-2-yloxycyclopropyl)methanol, PPh_3_, DEAD, THF, 0–25 °C; (vi) HATU, DIPEA, amine, DCM, 25 °C; (vii) HCl/dioxane.

Routes to methoxy modifications are outlined in Scheme 7. The phenol **70** was alkylated to form the ethers **71**–**73**, and then the nitrile of **71**–**73** was converted to the hydroxyamidines **74**–**76**. Condensation with ethyl 2-chloro-2-oxo-acetate and reaction with the amine **83** gave the amides **77**–**79**. Amine **83** was formed by converting the carboxylic acid of **68** to the amide, and then the Boc protecting group was removed from the amine group. To synthesize **80**, the hydroxy amidine **81** was condensed with ethyl 2-chloro-2-oxo-acetate to afford substituted oxadiazole ethyl ester **82**. The amide was formed directly from the ethyl ester of **82**, and **80** was formed by a Mitsunobu^22^ reaction.

**Scheme 8:**
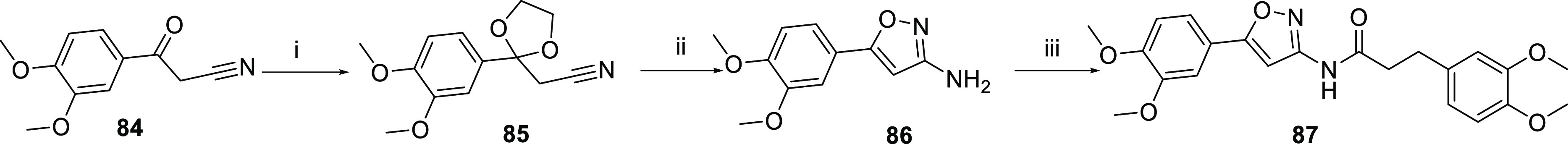
Route to Isoxazole Core with a Reverse Amide *Reagents and conditions*: (i) ethylene glycol, 4-methylbenzenesulfonic acid hydrate, toluene, 110 °C; (ii) hydroxylamine hydrochloride, 7 M NH_3_/methanol, quinolin-8-ol, MeOH, 70 °C, HCl, EtOH, 120 °C; (iii) 3-(3,4-dimethoxyphenyl)propanoic acid, thionyl chloride, DCM, 40 °C.

A route to the isoxazole core with a reverse amide is outlined in Scheme 8. The carbonyl of **84** was protected to form **85**. Formation of the hydroxy amidine and carbonyl deprotection and cyclization gave the isoxazole **86**. Reaction of the amine of **86** with 3-(3,4-dimethoxyphenyl)propanoic acid and thionyl chloride gave the amide **87**.

**Scheme 9:**
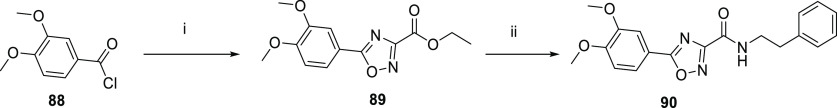
Route to Alternative 1,2,4-Oxadiazole Isomer *Reagents and conditions*: (i) ethyl 2-(hydroxyamino)-2-imino-acetate, triethylamine, DCM, 0 °C to rt; (ii) phenethylamine, triethylamine, MeOH, 60 °C.

A route to the alternative 1,2,4-oxadiazole isomer is outlined in Scheme 9. The 1,2,4-oxadiazole **89** was formed by reaction of the acid chloride **88** with ethyl 2-(hydroxyamino)-2-imino-acetate. The amide **90** was formed by reaction of the ester of **89** with phenethylamine.

**Scheme 10:**
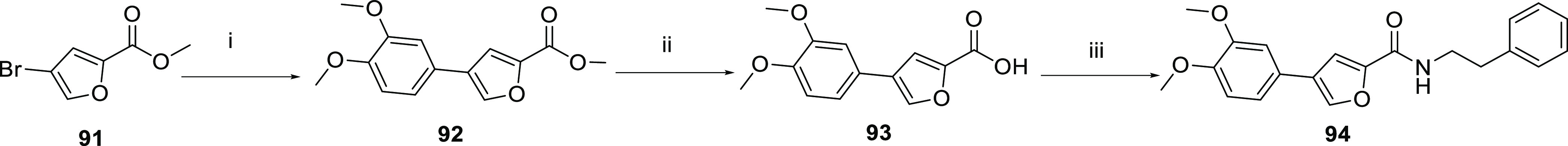
Route to Furan Core *Reagents and conditions*: (i) (3,4-dimethoxyphenyl)boronic acid, K_3_PO_4_, Pd(dtbpf)Cl_2_, triethylamine, DCM, N_2_ 80 °C; (ii) NaOH, water, EtOH, 80 °C; (iii) 2-phenylethanamine, HATU, triethylamine, DMF, rt.

A route to the furan core is outlined in Scheme 10. A Suzuki reaction^23^ on the bromide of **91** with (dimethoxyphenyl)boronic acid gave **92**. Hydrolysis of the methyl ester of **92** formed the carboxylic acid **93**, which was converted to the amide **94**.

**Scheme 11:**
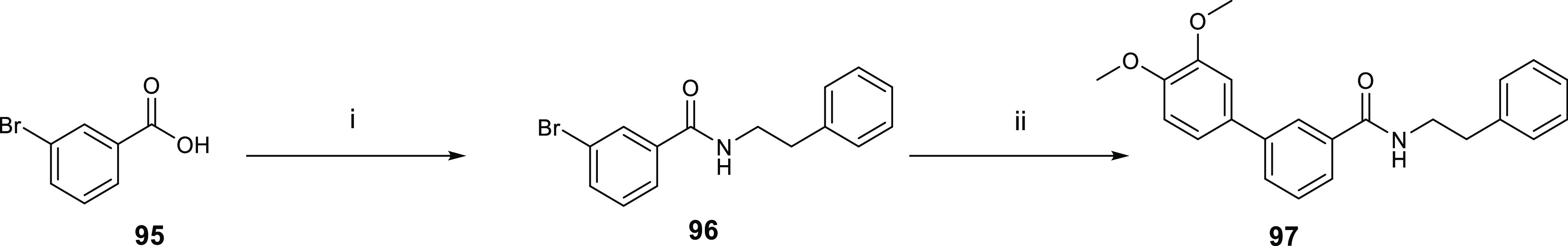
Route to Phenyl Core *Reagents and conditions*: (i) HATU, triethylamine, 2-phenylethanamine, DMF, rt; (ii) (3,4-dimethoxyphenyl)boronic acid, K_3_PO_4_, Pd(dtbpf)Cl_2_, triethylamine, DCM, N_2_ 80 °C.

A route to the phenyl core is outlined in Scheme 11. The carboxylic acid of **95** is converted to the amide **96**. A Suzuki reaction^23^ on the bromide of **96** with (dimethoxyphenyl)boronic acid gave **97**.

**Scheme 12:**
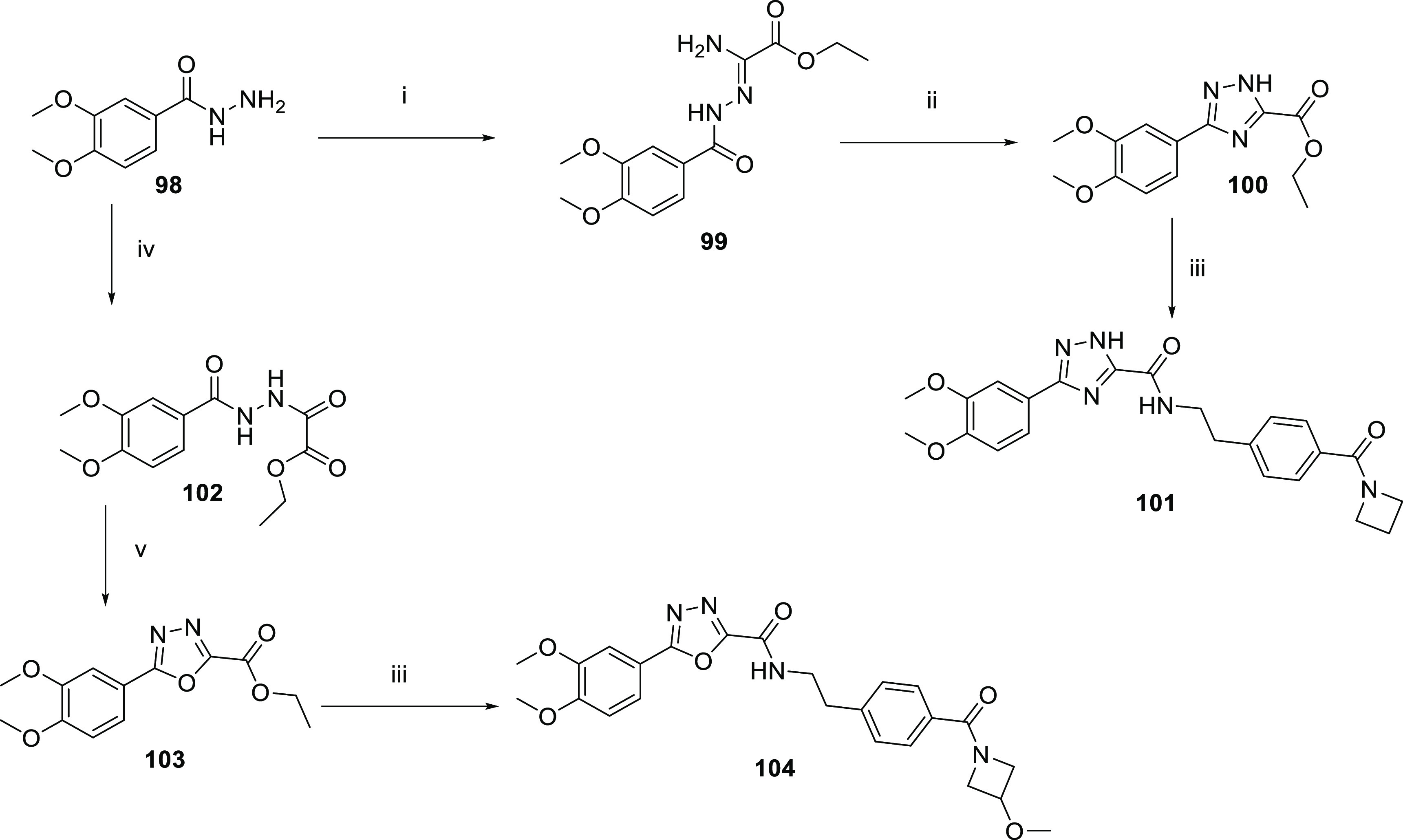
Routes to Triazole and 1,3,4-Oxadiazole Cores *Reagents and conditions*: (i) ethyl 2-amino-2-thioxo-acetate, 180 °C; (ii) AcOH, 100 °C; (iii) amine, triethylamine, MeOH, 60 °C; (iv) ethyl 2-chloro-2-oxoacetate, triethylamine, DCM, 0–25 °C; (v) pTsOH, DCM, 0–25 °C; (vi) HATU, DIPEA, amine, DCM, 25 °C; (vii) HCl/dioxane or TFA/DCM 25 °C.

Routes to the triazole and 1,3,4-oxadiazole cores are outlined in Scheme 12. The hydrazide **98** was reacted with ethyl 2-amino-2-thioxo-acetate to form intermediate **99**, which was cyclized to the triazole **100**. The ester of **100** was converted to the amide **101**. The hydrazide **98** was reacted with ethyl 2-chloro-2-oxoacetate to formthe intermediate **102**, and cyclization gave the 1,3,4-oxadiazole **103**. The ester of **103** was converted to the amide **104**.

## Results and Discussion

### *M. tuberculosis* Pks13 TE Domain Inhibitor Screening

A screening campaign was run on ∼150,000 compounds that came from seven different chemical libraries. These libraries represented a diverse collection of potential start points including: known phenotypic actives; a small polar molecule collection; the standard Dundee Drug Discovery Unit (DDU) compound collection; and the Gates Global Health Chemical Diversity Library.^24^ These compounds were tested at a single concentration (from 300 μM to 30 μM, depending on the library) in an *in vitro* Pks13 TE enzyme assay.^17^ From the initial screen, there were ∼1500 compounds that warranted further follow up, an initial hit rate of ∼1%.

**Figure 1:**
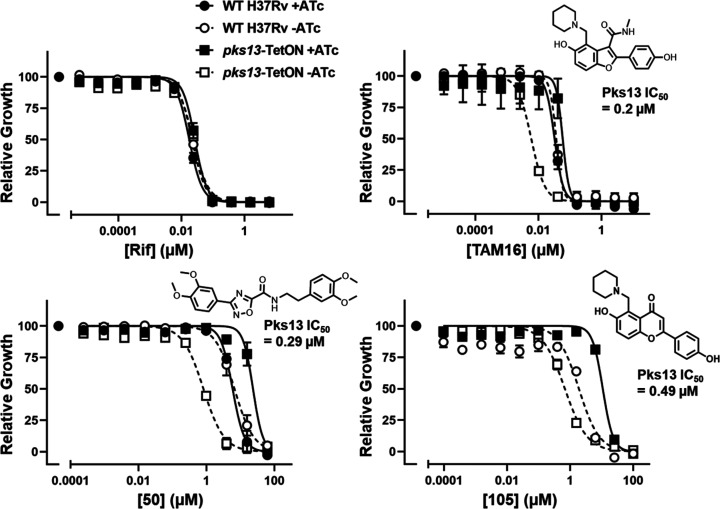
Dose response curves for compounds **50** and **105** tested against a tet-regulated Pks13 strain. Growth in the presence of anhydrotetracycline (ATc) results in modest transcriptional overexpression of *pks13*, while removal of ATc results in transcriptional repression of *pks13* expression. Growth in the presence of negative control (rifampicin), positive control (TAM16), **50**, and **105** is shown relative to DMSO-treated samples. Data are representative of two independent experiments.

Hit confirmation involved the following: (1) assessment in the *in vitro* enzyme assay over a dose response curve; (2) assessment in a counter screen to eliminate false positive compounds that interfered with fluorescence of the TE reaction product, 4-methylumbelliferone (Ex. 350 nm/Em. 450 nm); (3) assessment in a biolayer interferometry binding assay to confirm direct binding to the TE domain; and (4) evaluation in a Pks13 hypomorph strain (a strain of *M. tuberculosis* with the *pks13* gene under the control of the tetracycline promoter, so that expression levels can be controlled by the addition or removal of anhydrotetracycline [ATc]). Only two compounds, **50** and **105**, completed the assessment successfully, with the anticipated readout in the hypomorph strain in which overexpression of Pks13 (+ATc) reduced potency of the compounds, while underexpression of Pks13 (−ATc) increased compound potency (Figure 1). Both of these compounds had sub-micromolar IC_50_ values in the Pks13 enzyme assay, and they both gave a >10-fold shift in growth inhibitory MIC in the Pks13 hypomorph strain ± Atc (10.6-fold for **105** and >16-fold for **50**). Ten closely related analogues of **105**, with a chromone scaffold, were all active in the Pks13 assay and have also been reported elsewhere as Pks13 inhibitors.^25^ Significantly, there was a clear structural similarity between **105** and the original TE inhibitor TAM16; in particular, both contained the basic lipophilic piperidine group that was believed to be responsible for the off-target hERG channel inhibition that had resulted in the termination of the benzofuran series.^18^ It was determined experimentally that **105** did indeed strongly inhibit the hERG channel (Q-patch IC_50_ = 0.8 μM), and, as a result, no further work on this series was performed. On the other hand, **50** represented a very different, oxadiazole-based series with no obvious hERG liabilities (Q-patch IC_50_ > 20 μM) and, therefore, became the focus of further investigation.

### Crystal Structure of Pks13 TE Domain in Complex with **50**

**Figure 2:**
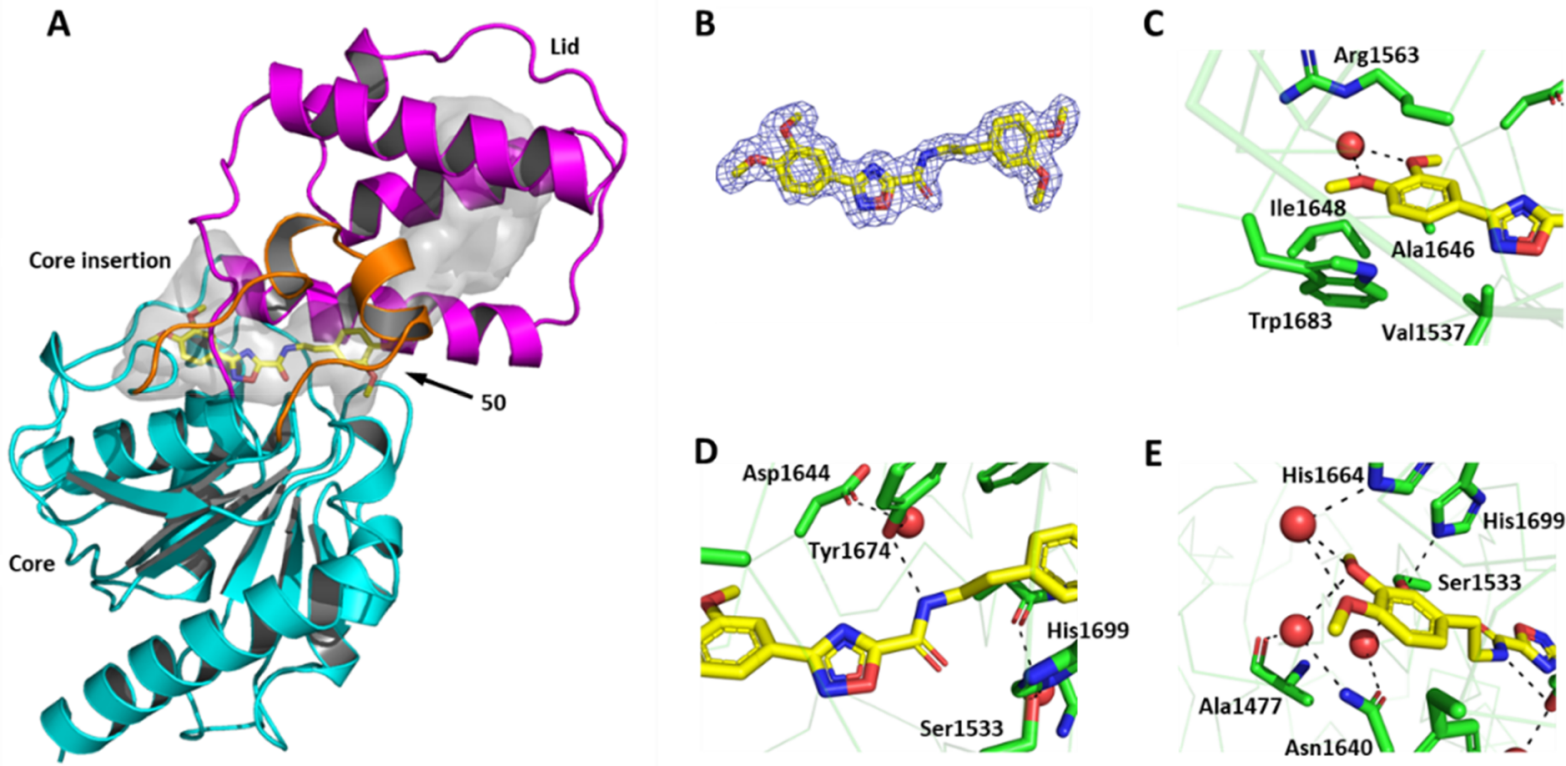
Novel binding mode of **50** to *M. tuberculosis* Pks13 thioesterase domain. Overall view of the structure of the Pks13-TE-**50** complex (PDB ID 8Q0T) represented as cartoon. An enlarged L-shaped binding pocket, shown as a gray tunnel, is the binding site for **50** (yellow sticks) (**A**). *2F*_o_ – *F*_c_ map (blue), contoured at 1σ around **50** (**B**). Close-up views of the interactions formed by binding of **50** show the dimethoxyphenyl and oxadiazole rings bound in the active site (**C**, **D**, and **E**). Residues and side chains within local proximity to the ligand are shown as sticks, while hydrogen bonds are represented by black dashed lines, with water molecules shown as red spheres.

To elucidate the binding mode of **50** within the Pks13 TE domain and to support the medicinal chemistry program, the X-ray co-crystal structure was determined. While conditions had been published previously^17^ for obtaining crystals of both the apo Pks13 TE domain (PDB 5V3W) and a back-soaking platform to produce complexes with a benzofuran inhibitor series (PDB 5V3Y), modifications to these conditions were required to allow suitable co-complexes to be determined. The structure of Pks13 TE domain in complex with **50** was solved at 1.8 Å resolution (Figure 2A). The Pks13 TE domain harbors a deep, long inverted “L” shaped hydrophobic substrate-binding site formed mostly by the lid region and, at the interface between the core, core insertion, and the lid region (Figure 2A). The long edge of the L-shape makes a surface-exposed “cavity” spanning a length of ∼30 Å, and the short edge of the L-shape makes a “tunnel” that extends deep below the catalytic triad (Ser1533, Asp1560, and His1699), spanning a length of ∼12 Å in the apo Pks13 TE domain.^17^ The unambiguous elongated stretch of electron density (Figure 2B) within the tunnel, confirmed that **50** binds in an extended orientation into the tunnel region of the substrate-binding site (Figure 2A). Unlike when the benzofuran inhibitors bind to the TE domain, there were no major rearrangements of the protein upon **50** binding, which takes up an orientation similar to that seen for the bound polypropylene glycol P-400 of the apo structure (PDB 5V3W). Upon **50** binding, the Arg1563 side-chain orientation shifts outward, resulting in the opening of the tunnel. It appears that the Arg1563 side-chain orientation determines the length of the tunnel, and it is crucial for holding substrates. The dimethoxyphenyl (ring A) sits at the bottom of the tunnel in a hydrophobic groove formed by Trp1683, Ile1648, Ala1646, and the aliphatic side chain of Arg1563 (Figure 2C). The phenyl ring makes a hydrophobic stacking interaction with the aliphatic side chain of Arg1563, and the methoxy groups both interact with a water molecule (Figure 2C). The oxadiazole ring is positioned at the center of the tunnel. The amide group is positioned close to the catalytic triad, with the nitrogen of the amide forming a H-bond to Tyr1674 (Figure 2D). The other dimethoxyphenyl (ring B) sits in a solvent-exposed cavity, at the interface between the core and the lid regions. The phenyl group lies close to the core insertion, while the two oxygen atoms of the methoxy groups become part of the water network and make water-mediated H-bonds with His1664, Asn1640, and the backbone carbonyl oxygen of Ala1477 (Figure 2E).

**Figure 3:**
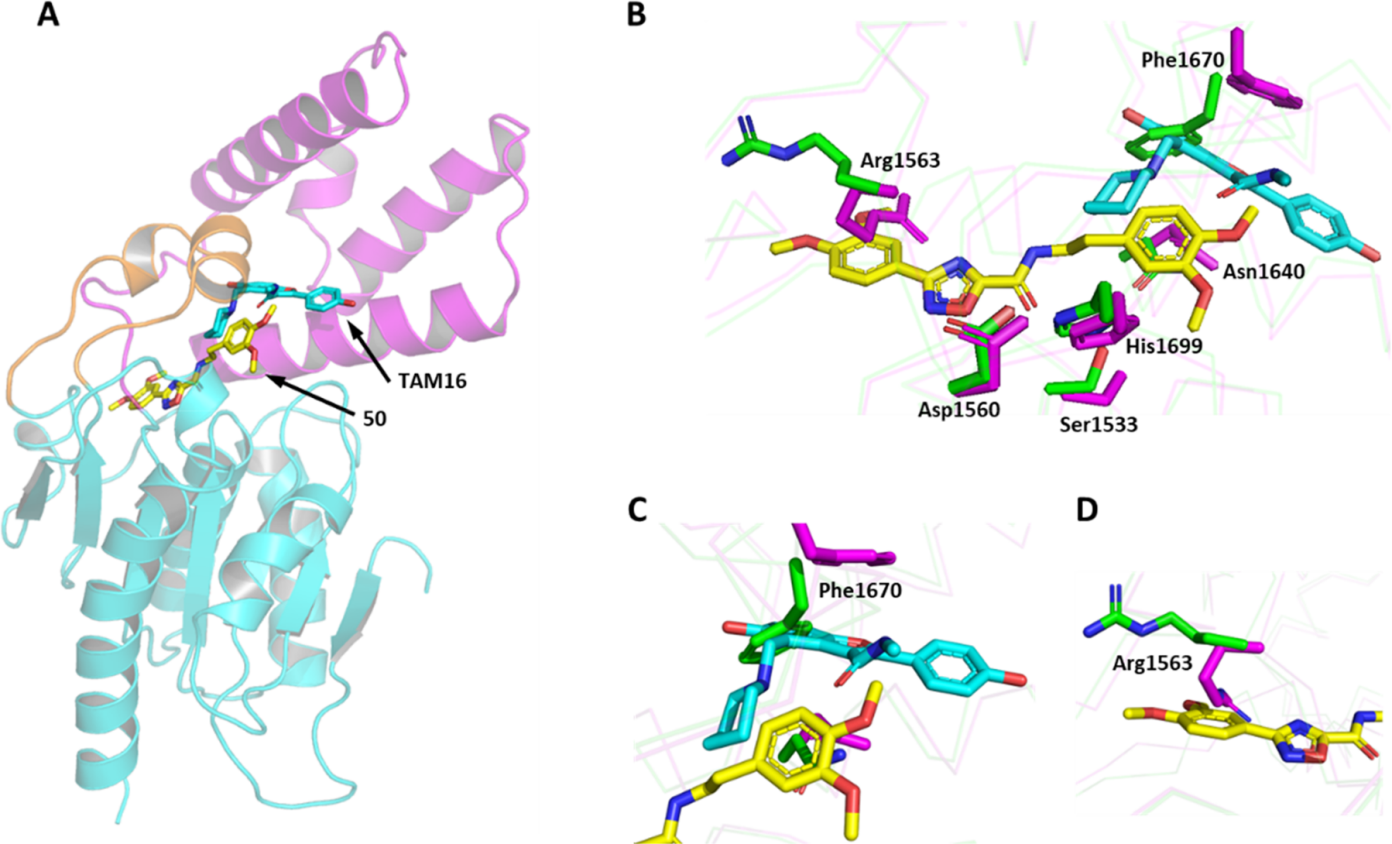
Differences in binding modes between TAM16 and **50** within the Pks13 TE domain, showing that structural rearrangement is required to accommodate different ligands. Cartoon representation of the relative orientations of **50** (yellow) and TAM16 (cyan) binding within the TE domain of Pks13 (**A**). Superimposition of Pks13-TAM16 (PDB ID 5V3Y: cyan and purple) with the Pks13-**50** (PDB ID 8Q0T: yellow and green) complexes, showing that significant structural rearrangement is required to allow the binding of TAM16 compared to **50** (**B**). **50** enters the tunnel, allowing it closer access to the catalytic triad (Ser1533, Asp1560, and His1699) in comparison to TAM16. Phe1670 “flip-out” from the **50**-bound structure (green) is required for TAM16 binding (purple) (**C**). Rearrangement of the side chain of Arg1563 is also required from the Pks13-TAM16 (purple) to that of Pks13-**50** (green), allowing the ligand to fully enter the tunnel, and a stacking interaction forms with the phenyl ring of the bound ligand (**D**).

The binding site for **50** was distinct from the previously reported binding site for the benzofuran inhibitors^17,18^ (Figure 3). Whereas **50** binds in the tunnel close to the catalytic triad, TAM16 binds at the interface between the lid and the core insertion; unlike **50**, TAM16 does not enter the tunnel containing the catalytic triad (Figure 3A). While the apo Pks13 TE domain does not show any major rearrangement after binding **50**, there are more significant perturbations when TAM16 is bound. Compared to the apo and **50**-bound structures, the Phe1670 side chain flips by about 80°, making space for the flat bicyclic benzofuran ring of TAM16 to lodge itself between Phe1670 and Asn1640. The phenyl ring of Phe1670 makes van der Waals stacking interactions with the furan ring, and a direct H-bond is formed between Asn1640 and the piperidine nitrogen within TAM16.^17^ Importantly, this Phe1670 “flip-out” conformation is required for TAM16 binding, as the apo and **50**-bound conformations are incompatible with TAM16 binding (Figure 3B,C). Additionally, the orientation of the side chain of Arg1563 in the apo and TAM16 structure is incompatible with **50** binding. Arg1563 reorients itself to open the bottom of the binding tunnel to allow compound access and forms hydrophobic stacking interactions with the phenyl ring of the bound ligand (Figure 3B,D).

### Initial SAR Exploration of Phenyl Substituents and Linker Length

Although the potency of **50** in the Pks13 assay was good, the MIC activity was modest. Initial SAR exploration involved simple modifications to **50** (Table 1). Shortening the linker length from ethyl to methyl (**3**) was not tolerated in the Pks13 assay. This was presumed to be due to the shorter linker not allowing the dimethoxyphenyl ring B to reach its solvent-exposed cavity, resulting in steric clashes with the residues at the mouth of the catalytic active-site tunnel, mainly Asn1640, Tyr1674, and Ser1533. Compound **50** has four methoxy groups that make hydrogen-bond interactions with the water network within the protein. To assess their contributions to potency, the methoxys were removed. From the crystal structure, it was clear that the methoxy groups on ring B were in a solvent-exposed area of the pocket and therefore probably had a low contribution to potency due to a high desolvation penalty. As expected, the phenethyl amide **106** retained potency against Pks13, although surprisingly it did lose MIC potency. The methoxy groups in ring A sit in a hydrophobic pocket, not as solvent-exposed as ring B, and make direct interactions with a water molecule. As such, it was anticipated that they would have a lower desolvation penalty for the interactions in this pocket and therefore a higher contribution to potency. As anticipated, the 3-phenyl-1,2,4-oxadiazole **107** had a modest impact on Pks13 potency (∼5-fold reduction). Removal of all four methoxy groups (**4**) reduced the potency further. The phenol analogue **20** also had a detrimental effect on potency and had no MIC activity. From this initial set of modifications, and the knowledge from the crystal structure that the dimethoxyphenyl ring B was in a solvent-exposed area, we anticipated that it was the most promising vector to modulate physicochemical properties, while retaining potency, by the introduction of polar groups or substituents that reduce lipophilicity.

**Table 1:**
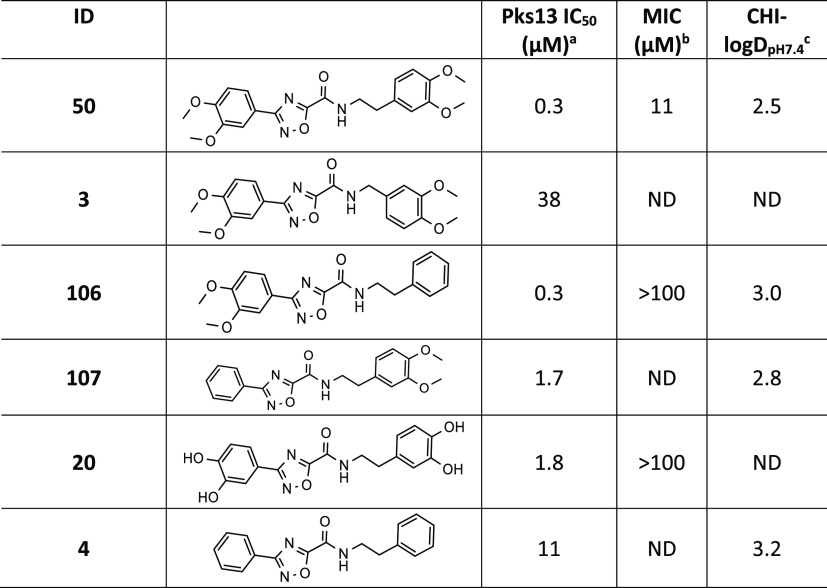
Preliminary Exploration of Phenyl Substitutions and Linker Length

a *M. tuberculosis* Pks13 TE domain 50% inhibitory concentration as assessed using the reported methodology.^17^

b H37Rv MIC is the minimum concentration required to inhibit the growth of *M. tuberculosis* (H37Rv) in liquid culture.

c CHI-LogD_pH7.4_ is a measure of lipophilicity at pH 7.4. ND = not determined.

### Dimethoxyphenyl (Ring B) SAR

Modifications to the dimethoxyphenyl of **50** were carried out to explore SAR, MIC potency, and *in vitro* metabolic stability, all of which needed to be improved (Table 2). Some of the modifications were aimed at replacing H-bonds with the water network, with direct H-bonds to the protein, in particular to residues Asn1640, His1664, and Asp1666 (Figure 2E). Changes to the dimethoxyphenyl group are summarized in Table 2. Removing one methoxy group (**109**, **19**) and replacing the 3,4-dimethoxyphenyl with pyridin-3-yl (**5**) retained Pks13 potency, but none showed an improvement in overall properties. The morpholine **108** had good *in vitro* metabolic stability, likely due to the reduction in lipophilicity, but lost potency against the enzyme. The benzoic acid **6** also had good *in vitro* metabolic stability and retained potency in the Pks13 assay but had no MIC activity. A set of amides were prepared to explore SAR; all retained Pks13 potency except for the dimethyl amide **13**. The primary amide and methyl amide **11** and **12**, although potent against Pks13, with good microsomal stability, had only modest MIC activity and poor *in vitro* hepatocyte stability. Amides with azetidine rings (**14**–**18**, **25**) all retained good activity against the Pks13 TE domain, and all had improved MIC potency. The crystal structure of the Pks13 TE domain bound with the azetidine amide **14** was solved and showed that the ligand binds in a similar orientation to **50**. As predicted by computational modeling, **14** had gained an extra direct H-bond interaction between the side chain of His1664 and the carbonyl oxygen of the azetidine (Figure S1A), thereby replacing a water molecule previously bound in the **50** structure. The azetidine ring orients away from the main bulk of the protein and out of the opening of the ligand-binding pocket, toward solvent. The azetidine amides were the first molecules to show an improved MIC potency. The improved activity was confirmed to still be on-target, through the use of the Pks13 hypomorph strain (Figure S2). The azetidine amide analogues also had improved *in vitro* mouse microsomal stability, but only the 2-oxa-6-azaspiro[3.3]heptane amide **18**, the analogue with the lowest CHI-logD, had mouse hepatocyte stability suitable for further progression. Overall, the majority of ring B modifications retained potency against the Pks13 TE domain, but many lost MIC activity. The azetidine amides had improved MIC activity, the 3-methoxyazetidine amide **15** was by far the most potent, and the 2-oxa-6-azaspiro[3.3]heptane amide **18** had the best overall properties.

**Table 2:**
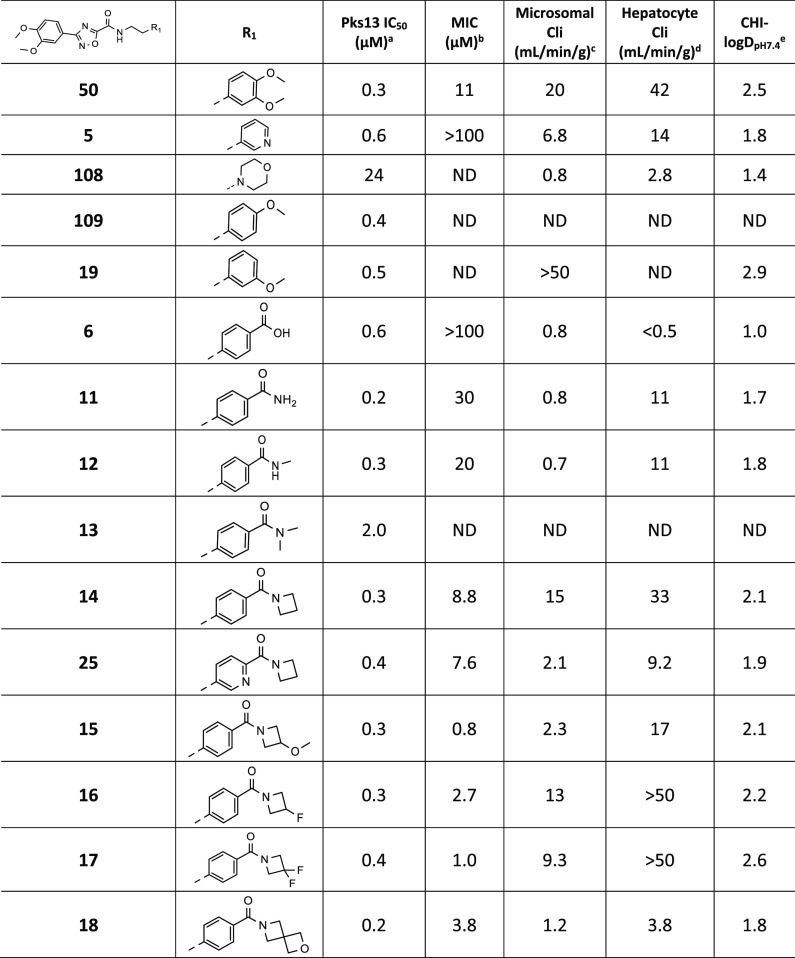
Dimethoxyphenyl (Ring B) SAR

a *M. tuberculosis* Pks13 TE domain 50% inhibitory concentration as assessed using the reported methodology.^17^

b H37Rv MIC is the minimum concentration required to inhibit the growth of *M. tuberculosis* (H37Rv) in liquid culture.

c Intrinsic microsomal clearance (Cli) using CD1 mouse liver microsomes.

d Intrinsic clearance (Cli) in mouse hepatocytes.

e CHI-LogDpH7.4 is a measure pf lipophilicity at pH 7.4. ND = not determined.

### Linker SAR

The crystal structure highlighted that the ethyl linker occupies a hydrophobic region. Its length is important to confer the correct geometry of ring B to avoid clashing with Asn1640, Tyr1674, and Ser1533, that line the tunnel and whose orientations appear to box the ligand in (Figure 2E). Despite the narrow binding site, the structural information inspired potential modifications to improve the ligand/protein shape complementarity. Modifications on the ethyl linker were explored with the unsubstituted phenyl to determine if enzyme inhibition could be improved (Table 3). The compound with the 1-hydroxy-3-phenylpropan-2-yl group (**110**) was not tolerated in the Pks13 assay. Introducing methyl groups on the linker next to the phenyl (**7**–**9**) was also detrimental to Pks13 potency. From the crystal structure, **50** binds in the active site close to the catalytic Ser1533; thus, there was the opportunity to target Ser1533 through a covalent interaction and thereby increase the potency of this series. The racemic nitrile analogue **38** was designed as a potential covalent inhibitor. However, while the nitrile was well tolerated and the Pks13 potency was maintained, it did not improve metabolic stability compared to **106**.

**Table 3:**
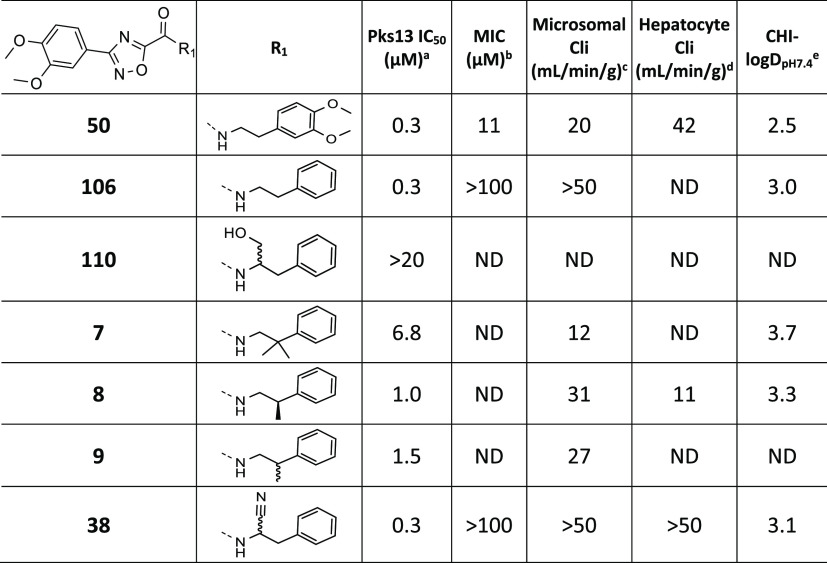
Linker SAR

a–e See Table 2 for explanation.

### Core SAR

The crystal structure of **50** indicated that there were no interactions with the 1,2,4-oxadiazole and that the amide sits in the middle of the narrow tunnel, establishing a H-bond with Tyr1674. The methyl amide **51** was inactive in the Pks13 assay (Table 4). This potency drop upon methylation could be due to a combination of the loss of a H-bond with the protein and a change in the amide conformation, triggered by the loss of the intramolecular H-bond, unfavorable for binding. The isoxazole **111** had good Pks13 potency but had no MIC activity and poor metabolic stability. Pks13 potency was maintained when the amide was reversed with this isoxazole core (**87**), but this compound still had no MIC activity and had poor microsomal stability. Removing the carbonyl (**53**) was detrimental to Pks13’s potency, suggesting basicity was not well tolerated. The alternative 1,2,4-oxadiazole isomer **90** reduced Pks13 potency. Replacing the heterocycle with a furan, phenyl, and triazole (**94**, **97**, and **101**) was detrimental to potency. As previously reported,^26^ the 1,3,4-oxadiazole **104** led to a reduction in lipophilicity and an improvement in mouse microsomal and hepatocyte stability but, disappointingly, displayed reduced potency compared to **15**. In brief, there were no improvements in overall properties with these heterocycle modifications.

**Table 4:**
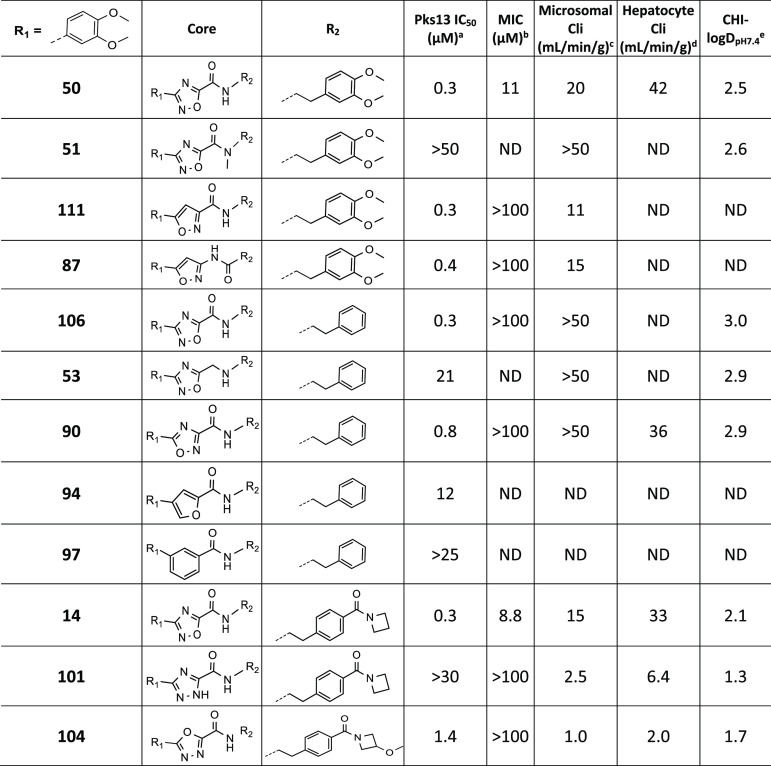
Core SAR

a–e See Table 2 for explanation.

### Dimethoxyphenyl (Ring A) Substitution SAR

The phenyl ring attached to the 1,2,4-oxadiazole establishes hydrophobic stacking interactions with Arg1563. The methoxy groups are positioned close to a small hydrophobic groove, consisting of Ala1646, Ile1648, Val1537, and Trp1683, and they interact with a water molecule (Figure 2C). However, the methoxy substituents were likely to be contributing to the compounds’ underlying metabolic instability. Therefore, substitution and changes to the aromatic ring were explored (Table 5), with the aim to maintain potency and improve metabolic stability. Replacing either methoxy group with a fluorine (**61**, **62**) modestly reduced Pks13 potency but eliminated MIC activity, although replacing the 4-methoxy did appear to improve metabolic stability. Removal of the 4-methoxy (**67**), although it had no impact on Pks13 inhibition, did not show the same improvement in stability, and it also reduced MIC activity. Replacing the 3,4-dimethoxyphenyl with benzimidazole (**63**) was detrimental to both Pks13 potency and MIC activity. The benzofuran, indole, and indazole (**64**–**66**) were all active in the Pks13 assay, but only **66** had some MIC activity. Guided by structural information, the 3-methoxy was replaced with other ethers to try and extend into a small lipophilic subpocket, to access additional molecular interactions. The 3-cyclopropylmethoxy, 3-cyclobutoxy, and 3-cyclopropoxy (**77**–**79**) were all well tolerated, but none of them offered a significant improvement over **15**. Introducing a hydroxy group on the cyclopropyl ring of **77** (**80**) reduced lipophilicity, resulting in an improvement in metabolic stability while displaying low micromolar MIC activity. In summary, changes to the 3-ether were tolerated in the MIC assay, and **80** had the best profile, with good MIC activity and mouse metabolic stability.

**Table 5:**
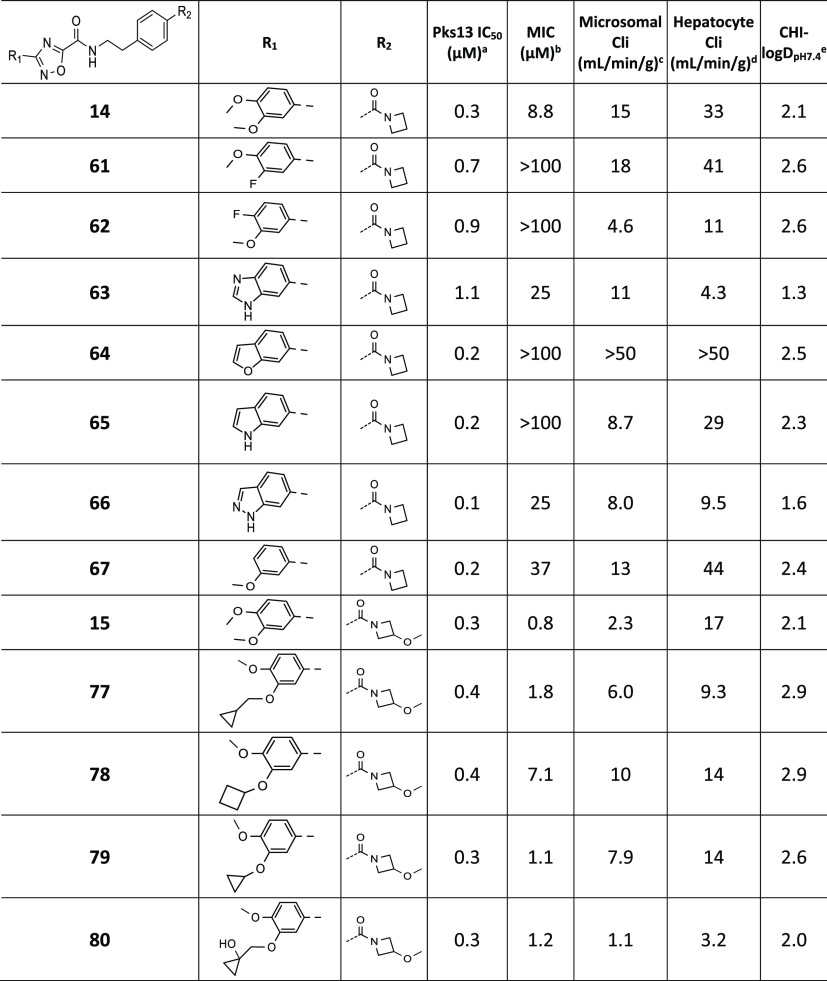
Dimethoxyphenyl (Ring A) SAR

a–e See Table 2 for explanation.

### Combination Modifications

Based on the SAR results described above, modifications with the most promising attributes were combined (Table 6). These all involved the inclusion of the nitrile substitution on the linker, as seen in **38**; the nitrile was of interest because it had the potential to form a covalent interaction with the catalytic Ser1533. The azetidine amide **33** had very good MIC activity but was not metabolically stable. A crystal structure was obtained of **33**, which adopted the same orientation in the elongated binding site as **50**. However, analysis of the crystal structure showed that the cyano group of **33** was positioned away from the oxyanion hole and did not make the anticipated covalent interaction with the active-site Ser1533 (Figure S1B). Instead, the cyano group lies close to the side chain of Asn1640, making a H-bond with Asn1640 itself, in addition to a highly conserved water molecule that makes specific H-bonds with side chains of Asp1644 and Tyr1674 and the main-chain carbonyl of Asn1640. Additionally, the carbonyl oxygen of the azetidine ring extends from the molecule, taking up a position in an orientation similar to that seen in the **14** structure, previously occupied by a water molecule bound between the methoxy groups of ring B of **50**. The 2-oxa-6-azaspiro[3.3]heptane amide **44** had good MIC activity and metabolic stability. The 3-methoxyazetidine amides **34** and **45** also had very good MIC activity and good microsomal stability, but, unfortunately, they had unacceptably low hepatocyte metabolic stability. Overall, although the nitrile analogues did not appear to form covalent interactions with the Pks13 TE domain, they were potent Pks13 inhibitors with improved MIC activity. Compound **44** had the best overall profile, combining submicromolar MIC activity with good mouse metabolic stability.

**Table 6:**
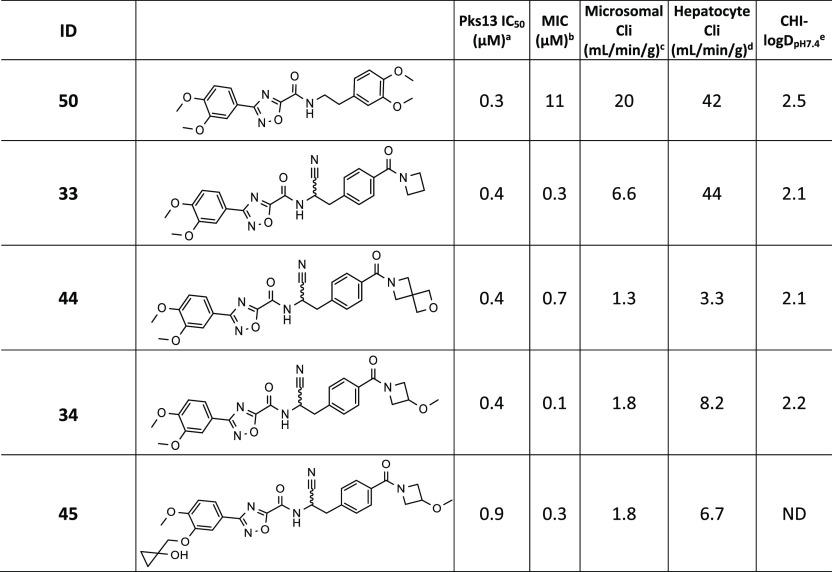
Combination Modifications

a–e See Table 2 for explanation.

### *In Vitro* DMPK and *In Vivo* Profiling of the Series

As the SAR for the series progressed, overall, there was excellent correlation between extracellular MIC and intramacrophage IC_90_. Human cell cytotoxicity was low for the compounds in the series, so the most promising compounds were profiled in pharmacokinetic studies to determine if any were suitable for evaluation in efficacy studies (Table 7). The three compounds evaluated were all well tolerated, with no adverse displays observed during the study. The inital compound to be tested was **15**, which was the first compound to have a MIC < 1.0 μM, although this compound did have poor *in vitro* hepatocyte metabolic stability (18 mL/min/g). The compound was dosed orally at 200 mg/kg, as this was the dose planned to be used for initial efficacy studies. Unfortunately, even at this high dose, the *in vivo* exposure was poor, with the *C*_max_ failing to get above that required to see antibacterial activity (∼500 ng/mL). Not unexpectedly, the *in vivo* clearance was poor (59 mL/min/g), in agreement with the poor *in vitro* hepatocyte stability. The next compound profiled was **80**, which had significantly improved metabolic stability and retained an MIC potency of ∼1 μM. In this case, **80** was dosed at a more standard 10 mg/kg, while the low *in vivo* exposure was explored. As anticipated from the *in vitro* data, there was an improved *in vivo* clearance for **80** (36 mL/min/g), but again it had poor *in vivo* exposure, failing to get close to the MIC levels. **80** had poor solubility (33 μM) and PAMPA permeability (14 nm/s), both of which may have been contributing to the poor *in vivo* exposure. The final compound profiled *in vivo* was **44**. It was the most polar compound and displayed good solubility (197 μM) but still suffered with poor PAMPA permeability (6 nm/s). Despite the improvement in solubility, when dosed orally, **44** had very poor exposure. Therefore, none of the three compounds tested had suitable oral exposure for progression into mouse models of acute TB infection. Potentially this could be related to the modest permeability for these three molecules and a more general issue for this chemotype, because none of the compounds from this series, tested in the PAMPA assay, demonstrated good permeability (>100 nm/s).

**Table 7:** Profile of Key Compounds from the Oxadiazole Series

	**15**		**80**		**44**
**Route (dose)**	**IV** (3 mg/kg)	**PO** (200 mg/kg)	**IV** (3 mg/kg)	**PO** (10 mg/kg)	**PO** (10 mg/kg)
***C***_**max**_**(ng/mL)**	–	151	–	61	56
***T***_**max**_**(h)**	–	2	–	0.5	5
***T***_**1/2**_**(h)**	0.3	–	0.4	–	–
**AUC**_**0–480**_**(μg·min/mL)**	56	48	81	4	5
**Clb** (mL/min/kg)	59	–	36	–	–
***V*_dss_** (L/kg)	1.1	–	0.9	–	–
***F* (%)**	–	1.5		1.5	–
**H37Rv MIC (μM)**^a^	0.8		1.2		0.7
**Intramacrophage (μM)**^b^	0.6		0.8		1.3
**HepG2 EC**_**50**_**(μM)**^c^	>100		>100		>100
**Stability micro.**^d^**/hep.**^e^ (mL/min/g)	2.3/17		1.1/3.2		1.3/3.3
**Aq. Solubility (μM)**^f^	19		33		197
**PAMPA Pe** (nm/s)^g^	57		14		6.1

a See Table 2 for explanation.

b Intramacrophage is the concentration required to inhibit 90% of the luminescent signal from a luciferase-expressing *M. tuberculosis* strain growing in THP1 monocytes.

c HepG2 50% inhibitory concentration.

d Intrinsic microsomal clearance (Cli) using CD1 mouse liver microsomes.

e Intrinsic clearance (Cli) in mouse hepatocytes.

f Aqueous solubility is kinetic aqueous solubility.

g PAMPA = parallel artificial membrane permeability assay.

## Conclusion

Pks13 is an attractive target for the identification of new TB treatments. Pks13 TE inhibitors have shown excellent activity in both acute and chronic TB mouse models^17,18^ but failed to advance to clinical trials due to cardiotoxicity risks.^18^ To identify alternative drug discovery starting points against this target, 150,000 compounds were screened in an *in vitro* thioesterase assay; a novel oxadiazole series was selected for further hit assessment. Testing against a Pks13 hypomorph strain confirmed that the oxadiazole hit **50**, killed bacteria by targeting Pks13. As this was a structurally enabled drug discovery program, the co-structure of **50** bound to Pks13 TE domain was acquired and showed a different binding mode compared to that of the previously published benzofuran series.^17^ Due to small conformational shifts on binding of the benzofuran, the two binding modes were incompatible and so did not allow the rational design of hybrid inhibitors. The oxadiazoles lie deeper in the substrate-binding pocket, close to the catalytic triad, and, as such, avoid the key interaction between benzofuran and protein, a H-bond between the protonated nitrogen of the piperidine with the carbonyl oxygen of the Asn1640 side chain. This was encouraging because the piperidine within the benzofuran series was responsible for the hERG inhibition and the resultant cardiovascular liability for that series.

Optimization of the oxadiazole hit focused on improving potency and metabolic stability. As seen for other Pks13 TE inhibitors,^17,18,27^ small changes in the chemical structure that had or would be expected to have minimal impact on enzyme inhibition, reduced or lost MIC potency. The reason for this disparity was not clear. The oxadiazole core was important for potency; alternative aromatic heterocycles were less potent or inactive. Changes to both phenyl rings were identified that improved MIC activity. Introduction of a nitrile into the ethyl linker, combined with changes in the phenyl ring, led to the greatest improvement in MIC potency (**33**, **34**, and **45**) for the series. Despite the jump in potency, a co-crystal structure of **33** bound to the Pks13 TE domain did not show evidence of a covalent interaction with the catalytic serine in the active site. Iterative rounds of design and synthesis improved the antitubercular potency by >200-fold and improved *in vitro* metabolic stability to be within acceptable limits for evaluation *in vivo* (<5 mL/min/g). Three lead molecules were selected for progression to mouse pharmacokinetic studies but, unfortunately, did not show suitable exposure for progression to *in vivo* efficacy studies. As oxadiazoles are components of known drugs, including antitubercular agents,^28,29^ there is potential for a successful further evaluation of this series, which is beyond the scope of this report. Moreover, while this optimization of the oxadiazole series was not successful, this work does demonstrate that a diverse range of scaffolds can inhibit the Pks13 thioesterase domain; therefore, screening for compounds with better drug-like properties should be encouraged.

## Experimental Section

### General Chemistry Methods

Chemicals and solvents were purchased from commercial vendors and were used as received, unless otherwise stated. Dry solvents were purchased in Sure Seal bottles stored over molecular sieves. Unless otherwise stated herein, reactions have not been optimized. Analytical thin-layer chromatography (TLC) was performed on precoated TLC plates (Kieselgel 60 F254, BDH). Developed plates were air-dried and analyzed under a UV lamp (UV 254/365 nm), and/or KMnO_4_ was used for visualization. Flash chromatography was performed using Combiflash Companion Rf (Teledyne ISCO) and prepacked silica gel columns purchased from Grace Davison Discovery Science or SiliCycle. Mass-directed preparative HPLC separations were performed using a Waters HPLC (2545 binary gradient pumps, 515 HPLC make-up pump, 2767 sample manager) connected to a Waters 2998 photodiode array and a Waters 3100 mass detector. Preparative HPLC separations were performed with a Gilson HPLC (321 pumps, 819 injection module, 215 liquid handler/injector) connected to a Gilson 155 UV/vis detector. On both instruments, HPLC chromatographic separations were conducted using Waters XBridge C18 columns, 19 mm × 100 mm, 5 μm particle size, using 0.1% ammonia in water (solvent A) and acetonitrile (solvent B) as mobile phase. ^1^H NMR spectra were recorded on a Bruker Advance II 500 or 400 spectrometer operating at 500 and 400 MHz (unless otherwise stated) using CDCl_3_, DMSO-*d*_6_, or CD_3_OD solutions. Chemical shifts (δ) are expressed in ppm, recorded using the residual solvent as the internal reference in all cases. Signal splitting patterns are described as singlet (s), doublet (d), triplet (t), multiplet (m), broadened (br), or a combination thereof. Coupling constants (*J*) are quoted to the nearest 0.1 Hz. Low-resolution electrospray (ES) mass spectra were recorded on a Bruker Daltonics MicroTOF mass spectrometer run in positive mode. High-resolution mass spectroscopy (HRMS) was performed using a Bruker Daltonics MicroTOf mass spectrometer. LC-MS analysis and chromatographic separation were conducted with either a Bruker Daltonics MicroTOF mass spectrometer connected to an Agilent diode array detector or a Thermo Dionex Ultimate 3000 RSLC system with a diode array detector, where the column used was a Waters XBridge column (50 mm × 2.1 mm, 3.5 μm particle size) and the compounds were eluted with a gradient of 5–95% acetonitrile/water + 0.1% ammonia, or with an Agilent Technologies 1200 series HPLC connected to an Agilent Technologies 6130 quadrupole LC-MS, connected to an Agilent diode array detector, where the column used was a Waters XBridge column (50 mm × 2.1 mm, 3.5 μm particle size) or a Waters X-select column (30 mm × 2.1 mm, 2.5 μm particle size) with a gradient of 5–90% acetonitrile/water + 0.1% formic acid; or with an Advion Expression mass spectrometer connected to a Thermo Dionex Ultimate 3000 HPLC with a diode array detector, where the column used was a Waters XBridge column (50 mm × 2.1 mm, 3.5 μm particle size) or a Waters X-select column (30 mm × 2.1 mm, 2.5 μm particle size) with a gradient of 5–90% acetonitrile/water + 0.1% formic acid. All final compounds showed chemical purity of ≥95% as determined from the UV chromatogram (190–450 nm) obtained by LC-MS analysis. Microwave-assisted chemistry was performed using a CEM or a Biotage microwave synthesizer.

### Methyl 3-(3,4-Dimethoxyphenyl)-1,2,4-oxadiazole-5-carboxylate (**2a**)

To a solution of *N*′-hydroxy-3,4-dimethoxy-benzamidine (1 g, 5.10 mmol) and triethylamine (1.4 mL, 10.2 mmol) in DCM (10 mL) at 0 °C was added methyl 2-chloro-2-oxo-acetate (0.7 mL, 7.64 mmol). The reaction mixture was stirred at 0 °C for 10 min and then heated to 40 °C for 16 h, concentrated *in vacuo*, dissolved in water (20 mL), extracted with EtOAc (3 × 20 mL), passed through a hydrophobic frit, and concentrated *in vacuo*. Purification by flash column chromatography afforded methyl 3-(3,4-dimethoxyphenyl)-1,2,4-oxadiazole-5-carboxylate (92 mg, 66%) as a white solid. ^1^H NMR (400 MHz, DMSO-*d*_6_): δ 7.65 (dd, *J* = 8.4, 2.0 Hz, 1H), δ 7.51 (d, *J* = 2.0 Hz, 1H), δ 7.16 (d, *J* = 8.5 Hz, 1H), δ 3.99 (s, 3H), δ 3.86 (s, 3H), δ 3.84 (s, 3H). LC-MS: *m*/*z* 265 [M+H]^+^.

### General Procedure A to Synthesize **3–5**, **7–10**, **20**

To a solution of methyl 3-(3,4-dimethoxyphenyl)-1,2,4-oxadiazole-5-carboxylate and amine (0.9 equiv) in methanol was added triethylamine (2–3 equiv). The reaction mixture was stirred at 60 °C for 16 h, concentrated *in vacuo*, dissolved in EtOAc (10 mL), washed with water (3 × 10 mL), passed through a hydrophobic frit, and concentrated *in vacuo*.

### 3-(3,4-Dimethoxyphenyl)-*N*-[(3,4-dimethoxyphenyl)methyl]-1,2,4-oxadiazole-5-carboxamide (**3**)

Following general procedure A, 3-(3,4-dimethoxyphenyl)-*N*-[(3,4-dimethoxyphenyl)methyl]-1,2,4-oxadiazole-5-carboxamide was obtained from methyl 3-(3,4-dimethoxyphenyl)-1,2,4-oxadiazole-5-carboxylate (100 mg, 0.38 mmol), 3,4-dimethoxyphenyl)methanamine (51 μL, 0.34 mmol), and triethylamine (95 μL, 0.68 mmol) in methanol (5 mL). Purification by trituration with 1:1 methanol:DMSO afforded 3-(3,4-dimethoxyphenyl)-*N*-[(3,4-dimethoxyphenyl)methyl]-1,2,4-oxadiazole-5-carboxamide (82 mg, 51%) as a white solid. ^1^H NMR (500 MHz, DMSO-*d*_6_): δ 9.89 (t, *J* = 6.1 Hz, 1H), 7.65 (dd, *J* = 8.4, 2.0 Hz, 1H), 7.54 (d, *J* = 2.0 Hz, 1H), 7.17 (d, *J* = 8.5 Hz, 1H), 6.99 (d, *J* = 1.7 Hz, 1H), 6.92–6.87 (m, 2H), 4.42 (d, *J* = 6.1 Hz, 2H), 3.85 (s, 6H), 3.75 (s, 3H), 3.73 (s, 3H). HRMS (ESI) calcd for [M+H]^+^ C_20_H_22_N_3_O_6_, 400.1509, found 400.1496.

### 3-Phenyl-*N*-(2-phenylethyl)-1,2,4-oxadiazole-5-carboxamide (**4**)

Following general procedure A, compound 3-phenyl-*N*-(2-phenylethyl)-1,2,4-oxadiazole-5-carboxamide was obtained from methyl 3-phenyl-1,2,4-oxadiazole-5-carboxylate (246 mg, 1.20 mmol), 2-phenylethanamine (131 mg, 1.08 mmol), and triethylamine (0.5 mL, 3.61 mmol) in methanol (5 mL). Purification by prep-HPLC afforded 3-phenyl-*N*-(2-phenylethyl)-1,2,4-oxadiazole-5-carboxamide (145 mg, 39%) as a white solid. ^1^H NMR (500 MHz, DMSO-*d*_6_): δ 9.55 (s, 1H), 8.07–8.05 (m, 2H), 7.66–7.59 (m, 3H), 7.32–7.29 (m, 2H), 7.27–7.25 (m, 2H), 7.23–7.20 (m, 1H), 3.56–3.53 (m, 2H), 2.89 (t, *J* = 6.0 Hz, 2H). LC-MS: *m*/*z* 294 [M+H]^+^.

### 3-(3,4-Dimethoxyphenyl)-*N*-[2-(3-pyridyl)ethyl]-1,2,4-oxadiazole-5-carboxamide (**5**)

Following general procedure A, compound 3-(3,4-dimethoxyphenyl)-*N*-[2-(3-pyridyl)ethyl]-1,2,4-oxadiazole-5-carboxamide was obtained from methyl 3-(3,4-dimethoxyphenyl)-1,2,4-oxadiazole-5-carboxylate (60 mg, 0.23 mmol), 2-(3-pyridyl)ethanamine hydrochloride (32 mg, 0.20 mmol), and triethylamine (79 μL, 0.57 mmol) in methanol (5 mL). Purification by prep-HPLC afforded 3-(3,4-dimethoxyphenyl)-*N*-[2-(3-pyridyl)ethyl]-1,2,4-oxadiazole-5-carboxamide (25 mg, 30%) as a white solid. ^1^H NMR (500 MHz, DMSO-*d*_6_): δ 9.53 (t, *J* = 5.6 Hz, 1H), 8.48 (d, *J* = 1.9 Hz, 1H), 8.43 (dd, *J* = 4.8, 1.6 Hz, 1H), 7.67 (dt, *J* = 7.9, 1.9 Hz, 1H), 7.65 (dd, *J* = 8.4, 2.0 Hz, 1H), 7.53 (d, *J* = 2.0 Hz, 1H), 7.33 (dd, *J* = 7.8, 4.8 Hz, 1H), 7.17 (d, *J* = 8.5 Hz, 1H), 3.85 (s, 6H), 3.59–3.55 (m, 2H), 2.92 (t, *J* = 7.2 Hz, 2H). LC-MS: *m*/*z* 355 [M+H]^+^.

### 4-[2-[[3-(3,4-Dimethoxyphenyl)-1,2,4-oxadiazole-5-carbonyl]amino]ethyl]benzoic Acid (**6**)

To a solution of 4-(2-aminoethyl)benzoic acid hydrochloride (274 mg, 1.36 mmol) and methyl 3-(3,4-dimethoxyphenyl)-1,2,4-oxadiazole-5-carboxylate (400 mg, 1.51 mmol) in methanol (5 mL) was added triethylamine (0.6 mL, 4.54 mmol). The reaction mixture was stirred at 60 °C for 16 h, concentrated *in vacuo*, dissolved in EtOAc (10 mL), washed with 2 M HCl (3 × 10 mL), passed through a hydrophobic frit, and concentrated *in vacuo* to afford 4-[2-[[3-(3,4-dimethoxyphenyl)-1,2,4-oxadiazole-5-carbonyl]amino]ethyl]benzoic acid (516 mg, 85%) as a white solid. ^1^H NMR (500 MHz, DMSO-*d*_6_): δ 12.76 (br s, 1H), 9.58 (t, *J* = 5.7 Hz, 1H), 7.83 (d, *J* = 8.1 Hz, 2H), 7.6 (dd, *J* = 8.3, 2.0 Hz, 1H), 7.53 (d, *J* = 2.0 Hz, 1H), 7.27 (d, *J* = 7.9 Hz, 2H), 7.16 (d, *J* = 8.5 Hz, 1H), 3.85 (s, 6H), 3.57 (q, *J* = 6.8 Hz, 2H), 2.92 (t, *J* = 7.4 Hz, 2H). LC-MS: *m*/*z* 398 [M+H]^+^.

### 3-(3,4-Dimethoxyphenyl)-*N*-(2-methyl-2-phenyl-propyl)-1,2,4-oxadiazole-5-carboxamide (**7**)

Following general procedure A, compound 3-(3,4-dimethoxyphenyl)-*N*-(2-methyl-2-phenyl-propyl)-1,2,4-oxadiazole-5-carboxamide was obtained from methyl 3-(3,4-dimethoxyphenyl)-1,2,4-oxadiazole-5-carboxylate (100 mg, 0.38 mmol), 2-methyl-2-phenyl-propan-1-amine hydrochloride (63 mg, 0.34 mmol), and triethylamine (132 μL, 0.95 mmol) in methanol (5 mL). Purification by prep-HPLC afforded 3-(3,4-dimethoxyphenyl)-*N*-(2-methyl-2-phenyl-propyl)-1,2,4-oxadiazole-5-carboxamide (76 mg, 50%) as a yellow oil. ^1^H NMR (500 MHz, DMSO-*d*_6_): δ 9.10 (br s, 1H), 7.64 (dd, *J* = 8.4, 2.0 Hz, 1H), 7.51 (d, *J* = 2.0 Hz, 1H), 7.45–7.43 (m, 2H), 7.36–7.33 (m, 2H), 7.24–7.21 (m, 1H), 7.16 (d, *J* = 8.5 Hz, 1H), 3.85 (s, 6H), 3.51 (s, 2H), 1.33 (s, 6H). LC-MS: *m*/*z* 382 [M+H]^+^.

### 3-(3,4-Dimethoxyphenyl)-*N*-[(2*R*)-2-phenylpropyl]-1,2,4-oxadiazole-5-carboxamide (**8**)

Following general procedure A, in a sealed microwave tube 3-(3,4-dimethoxyphenyl)-*N*-[(2*R*)-2-phenylpropyl]-1,2,4-oxadiazole-5-carboxamide was obtained from methyl 3-(3,4-dimethoxyphenyl)-1,2,4-oxadiazole-5-carboxylate (100 mg, 0.37 mmol) in MeOH (5 mL), (2*R*)-2-phenylpropan-1-amine (51 mg, 0.37 mmol), and Et_3_N (0.13 mL, 0.94 mmol). Purification by mass-directed HPLC with 5–95% MeCN acidic afforded 3-(3,4-dimethoxyphenyl)-*N*-[(2*R*)-2-phenylpropyl]-1,2,4-oxadiazole-5-carboxamide (87 mg, 59%). ^1^H NMR (500 MHz, DMSO): δ 9.44 (t, *J* = 5.8 Hz, 1H), 7.65 (dd, *J* = 2.0, 8.4 Hz, 1H), 7.53 (d, *J* = 2.0 Hz, 1H), 7.35–7.27 (m, 4H), 7.24–7.17 (m, 2H), 3.86 (s, 6H), 3.53–3.41 (m, 2H), 3.16–3.10 (m, 1H), 1.25 (d, *J* = 7.0 Hz, 3H). HRMS (ESI) calcd for [M+H]^+^ C_20_H_22_N_3_O_4_, 368.1605, found 368.1594.

### 3-(3,4-Dimethoxyphenyl)-*N*-(2-phenylpropyl)-1,2,4-oxadiazole-5-carboxamide (Racemic) (**9**)

Following general procedure A in a sealed microwave tube, compound 3-(3,4-dimethoxyphenyl)-*N*-(2-phenylpropyl)-1,2,4-oxadiazole-5-carboxamide was obtained from methyl 3-(3,4-dimethoxyphenyl)-1,2,4-oxadiazole-5-carboxylate (100 mg, 0.37 mmol), 2-phenylpropan-1-amine (51 mg, 0.37 mmol), and Et_3_N (0.13 mL, 0.94 mmol). Purification by mass-directed HPLC with 5–95% MeCN acidic afforded 3-(3,4-dimethoxyphenyl)-*N*-(2-phenylpropyl)-1,2,4-oxadiazole-5-carboxamide (71 mg, 48%). ^1^H NMR (500 MHz, DMSO): δ 9.44 (t, *J* = 5.4 Hz, 1H), 7.65 (dd, *J* = 2.0, 8.4 Hz, 1H), 7.53–7.52 (m, 1H), 7.35–7.17 (m, 6H), 3.86 (s, 6H), 3.53–3.41 (m, 2H), 3.16–3.10 (m, 1H), 1.25 (d, *J* = 6.9 Hz, 3H). HRMS (ESI) calcd for [M+H]^+^ C_20_H_21_N_3_O 368.1610, found 368.1615.

### 3-(3,4-Dimethoxyphenyl)-*N*-(3-hydroxyphenethyl)-1,2,4-oxadiazole-5-carboxamide (**10**)

Following general procedure A, 3-(3,4-dimethoxyphenyl)-*N*-(3-hydroxyphenethyl)-1,2,4-oxadiazole-5-carboxamide was obtained from methyl 3-(3,4-dimethoxyphenyl)-1,2,4-oxadiazole-5-carboxylate (200 mg, 0.76 mmol), 3-(2-aminoethyl)phenol hydrochloride (118 mg, 0.68 mmol), and triethylamine (317 μL, 2.27 mmol) in methanol (5 mL). Purification by prep-HPLC afforded 3-(3,4-dimethoxyphenyl)-*N*-(3-hydroxyphenethyl)-1,2,4-oxadiazole-5-carboxamide (156 mg, 50%) as a white solid. ^1^H NMR (500 MHz, DMSO-*d*_6_): δ 9.48 (t, *J* = 5.7 Hz, 1H), 9.27 (s, 1H), 7.65 (dd, *J* = 8.3, 1.9 Hz, 1H), 7.53 (d, *J* = 1.9 Hz, 1H), 7.16 (d, *J* = 8.4 Hz, 1H), 7.10–7.07 (m, 1H), 6.67–6.65 (m, 2H), 6.62–6.60 (m, 1H), 3.86 (s, 3H), 3.85 (s, 3H), 3.49 (q, *J* = 7.0 Hz, 2H), 2.80 (t, *J* = 7.5 Hz, 2H). LC-MS: *m*/*z* 370 [M+H]^+^.

### *N*-[2-(4-Carbamoylphenyl)ethyl]-3-(3,4-dimethoxyphenyl)-1,2,4-oxadiazole-5-carboxamide (**11**)

To a solution of 4-[2-[[3-(3,4-dimethoxyphenyl)-1,2,4-oxadiazole-5-carbonyl]amino]ethyl]benzoic acid (100 mg, 0.25 mmol) in THF (5 mL) were added ammonia HOBt (57 mg, 0.38 mmol), EDCI·HCl (72 mg, 0.38 mmol), and DIPEA (110 μL, 0.63 mmol). The reaction mixture was stirred at rt for 16 h, diluted with water (10 mL), extracted with EtOAc (3 × 10 mL), passed through a hydrophobic frit, and concentrated *in vacuo*. Purification by prep-HPLC afforded *N*-[2-(4-carbamoylphenyl)ethyl]-3-(3,4-dimethoxyphenyl)-1,2,4-oxadiazole-5-carboxamide (41 mg, 37%) as a white solid. ^1^H NMR (500 MHz, DMSO-*d*_6_): δ 9.51 (br s, 1H), 7.89 (br s, 1H), 7.82 (d, *J* = 8.2 Hz, 2H), 7.65 (dd, *J* = 8.4, 1.9 Hz, 1H), 7.53 (d, *J* = 1.9 Hz, 1H), 7.33 (d, *J* = 8.2 Hz, 2H), 7.26 (br s, 1H), 7.17 (d, *J* = 8.5 Hz, 1H), 3.85 (s, 6H), 3.58–3.55 (m, 2H), 2.94 (t, *J* = 7.3 Hz, 2H). LC-MS: *m*/*z* 395 [M–H]^−^.

### General Procedure B to Synthesize **12–14**

To a solution of 4-[2-[[3-(3,4-dimethoxyphenyl)-1,2,4-oxadiazole-5-carbonyl]amino]ethyl]benzoic acid (100 mg, 0.25 mmol), T3P (0.3 mL, 0.28 mmol), and triethylamine (0.1 mL, 0.75 mmol) in DMF (5 mL) was added amine (0.28 mmol). The reaction mixture was stirred at rt for 16 h, concentrated *in vacuo*, diluted with EtOAc (20 mL), washed with water (3 × 20 mL), passed through a hydrophobic frit, and concentrated *in vacuo*.

### 3-(3,4-Dimethoxyphenyl)-*N*-[2-[4-(methylcarbamoyl)phenyl]ethyl]-1,2,4-oxadiazole-5-carboxamide (**12**)

Following general procedure B, compound 3-(3,4-dimethoxyphenyl)-*N*-[2-[4-(methylcarbamoyl)phenyl]ethyl]-1,2,4-oxadiazole-5-carboxamide was obtained from 4-[2-[[3-(3,4-dimethoxyphenyl)-1,2,4-oxadiazole-5-carbonyl]amino]ethyl]benzoic acid (100 mg, 0.25 mmol), T3P (0.16 mL, 0.28 mmol), triethylamine (0.1 mL, 0.75 mmol), and methylamine hydrochloride (18 mg, 0.28 mmol) in DMF (5 mL). Purification by prep-HPLC afforded 3-(3,4-dimethoxyphenyl)-*N*-[2-[4-(methylcarbamoyl)phenyl]ethyl]-1,2,4-oxadiazole-5-carboxamide (43 mg, 37%) as a white solid. ^1^H NMR (500 MHz, DMSO-*d*_6_): δ 9.52 (t, *J* = 5.6 Hz, 1H), 8.35–8.33 (m, 1H), 7.77 (d, *J* = 8.2 Hz, 2H), 7.65 (dd, *J* = 8.4, 1.9 Hz, 1H), 7.52 (d, *J* = 1.9 Hz, 1H), 7.34 (d, *J* = 8.2 Hz, 2H), 7.17 (d, *J* = 8.5 Hz, 1H), 3.84 (s, 6H), 3.58–3.54 (m, 2H), 2.94 (t, *J* = 7.4 Hz, 2H), 2.77 (d, *J* = 4.6 Hz, 3H). LC-MS: *m*/*z* 411 [M+H]^+^.

### 3-(3,4-Dimethoxyphenyl)-*N*-[2-[4-(dimethylcarbamoyl)phenyl]ethyl]-1,2,4-oxadiazole-5-carboxamide (**13**)

Following general procedure B, 3-(3,4-dimethoxyphenyl)-*N*-[2-[4-(dimethylcarbamoyl)phenyl]ethyl]-1,2,4-oxadiazole-5-carboxamide was obtained from 4-[2-[[3-(3,4-dimethoxyphenyl)-1,2,4-oxadiazole-5-carbonyl]amino]ethyl]benzoic acid (100 mg, 0.25 mmol), T3P (0.3 mL, 0.28 mmol), triethylamine (0.1 mL, 0.75 mmol), and dimethylamine hydrochloride (23 mg, 0.28 mmol) in DMF (5 mL). Purification by prep-HPLC afforded 3-(3,4-dimethoxyphenyl)-*N*-[2-[4-(dimethylcarbamoyl)phenyl]ethyl]-1,2,4-oxadiazole-5-carboxamide (3 mg, 3%) as a white solid. ^1^H NMR (500 MHz, DMSO-*d*_6_): δ 9.56 (t, *J* = 5.7 Hz, 1H), 7.65 (dd, *J* = 8.3, 2.0 Hz, 1H), 7.52 (d, *J* = 1.9 Hz, 1H), 7.35–7.30 (m, 4H), 7.17 (d, *J* = 8.5 Hz, 1H), 3.85 (s, 6H), 3.57–3.55 (q, *J* = 7.0 Hz, 2H), 2.96–2.90 (m, 8H). LC-MS: *m*/*z* 425 [M+H]^+^.

### N-[2-[4-(Azetidine-1-carbonyl)phenyl]ethyl]-3-(3,4-dimethoxyphenyl)-1,2,4-oxadiazole-5-carboxamide (**14**)

Following general procedure B, *N*-[2-[4-(azetidine-1-carbonyl)phenyl]ethyl]-3-(3,4-dimethoxyphenyl)-1,2,4-oxadiazole-5-carboxamide was obtained from 4-[2-[[3-(3,4-dimethoxyphenyl)-1,2,4-oxadiazole-5-carbonyl]amino]ethyl]benzoic acid (100 mg, 0.25 mmol), T3P (0.3 mL, 0.28 mmol), triethylamine (0.1 mL, 0.75 mmol), and azetidine (16 mg, 0.28 mmol) in DMF (5 mL). Purification by prep-HPLC afforded *N*-[2-[4-(azetidine-1-carbonyl)phenyl]ethyl]-3-(3,4-dimethoxyphenyl)-1,2,4-oxadiazole-5-carboxamide (65 mg, 53%) as a white solid. ^1^H NMR (500 MHz, DMSO-*d*_6_): δ 9.56 (s, 1H), 7.65 (dd, *J* = 8.4, 2.0 Hz, 1H), 7.57 (d, *J* = 8.2 Hz, 2H), 7.52 (d, *J* = 1.9 Hz, 1H), 7.32 (d, *J* = 8.2 Hz, 2H), 7.17 (d, *J* = 8.5 Hz, 1H), 4.28 (t, *J* = 7.5 Hz, 2H), 4.02 (t, *J* = 7.6 Hz, 2H), 3.85 (s, 6H), 3.55 (t, *J* = 7.2 Hz, 2H), 2.93 (t, *J* = 7.4 Hz, 2H), 2.24 (quin, *J* = 7.7 Hz, 2H). HRMS (ESI): calcd for [M+H]^+^ C_23_H_25_N_4_O_5_, 437.1825, found 437.1840.

### 3-(3,4-Dimethoxyphenyl)-*N*-(4-(3-methoxyazetidine-1-carbonyl)phenethyl)-1,2,4-oxadiazole-5-carboxamide (**15**)

To a solution of 4-[2-[[3-(3,4-dimethoxyphenyl)-1,2,4-oxadiazole-5-carbonyl]amino]ethyl]benzoic acid (0.95 g, 2.39 mmol, 1 equiv) and 3-methoxyazetidine hydrochloride (354 mg, 2.87 mmol, 1.2 equiv) in dry DMF (20 mL) were added HATU (1.36 g, 3.59 mmol, 1.5 equiv) and DIPEA (926 mg, 7.17 mmol, 1.25 mL, 3 equiv) in turns below 0 °C. The resulting mixture was stirred at 20 °C for 1 h. The reaction mixture was poured into water (20 mL) and extracted with ethyl acetate (30 mL × 5). The combined organic layers were washed with water (20 mL × 2) and brine (20 mL) and dried over Na_2_SO_4_. After filtration and concentration, the filtrate was concentrated *in vacuo*. The residue was dissolved in DMF (20 mL). The precipitate was collected by filtration and washed with MeOH (20 mL). The filtrate cake was dried *in vacuo* to afford 3-(3,4-dimethoxyphenyl)-*N*-(4-(3-methoxyazetidine-1-carbonyl)phenethyl)-1,2,4-oxadiazole-5-carboxamide (681 mg, 1.46 mmol, 61% yield) as a white solid. ^1^H NMR (400 MHz, DMSO-*d*_6_): δ 9.56 (br t, *J* = 5.7 Hz, 1H), 7.65 (dd, *J* = 1.8, 8.4 Hz, 1H), 7.58 (d, *J* = 8.1 Hz, 2H), 7.52 (d, *J* = 1.8 Hz, 1H), 7.34 (d, *J* = 8.1 Hz, 2H), 7.17 (d, *J* = 8.4 Hz, 1H), 4.41 (br s, 1H), 4.21 (br d, *J* = 5.0 Hz, 2H), 4.11 (br d, *J* = 7.8 Hz, 1H), 3.85 (s, 7H), 3.56 (q, *J* = 6.9 Hz, 2H), 3.21 (s, 3H), 2.94 (br t, *J* = 7.3 Hz, 2H). ^13^C NMR (125 MHz, DMSO): δ 169.5, 169.4, 168.4, 153.4, 152.3, 149.6, 142.6, 131.5, 129.1, 128.3, 121.2, 118.2, 112.5, 110.2, 69.4, 60.2, 56.2, 56.1, 55.8, 40.8, 34.7. HRMS (ESI): calcd for [M+H]^+^ C_24_H_27_N_4_O_6_, 467.1931, found 467.1925.

### General Procedure C to Synthesize **16–18**

To a mixture of 4-(2-(3-(3,4-dimethoxyphenyl)-1,2,4-oxadiazole-5-carboxamido)ethyl)benzoic acid (100 mg, 251 μmol, 1 equiv) and the corresponding amine (1.2 equiv) in DMF (1 mL) were added HATU (143 mg, 377 μmol, 1.5 equiv) and DIPEA (97 mg, 754 μmol, 131 μL, 3 equiv) in turns at 25 °C. The mixture was stirred at 25 °C for 1 to 12 h. The residue was poured into water (10 mL). The aqueous phase was extracted with ethyl acetate (5 mL × 3). The combined organic phase was washed with brine (5 mL), dried with anhydrous Na_2_SO_4_, filtered, and concentrated *in vacuo*. The residue was purified by prep-HPLC.

### 3-(3,4-Dimethoxyphenyl)-*N*-(4-(3-fluoroazetidine-1-carbonyl)phenethyl)-1,2,4-oxadiazole-5-carboxamide (**16**)

Following general procedure C, 3-(3,4-dimethoxyphenyl)-*N*-(4-(3-fluoroazetidine-1-carbonyl)phenethyl)-1,2,4-oxadiazole-5-carboxamide was obtained from 4-(2-(3-(3,4-dimethoxyphenyl)-1,2,4-oxadiazole-5-carboxamido)ethyl)benzoic acid (100 mg, 251 μmol, 1 equiv) and 3-fluoroazetidine (33 mg, 301 μmol, 1.2 equiv, HCl salt) after 1 h under stirring. Purification by prep-HPLC (water 0.225%FA–ACN]; B%: 33–55%, 9 min) afforded 3-(3,4-dimethoxyphenyl)-*N*-(4-(3-fluoroazetidine-1-carbonyl)phenethyl)-1,2,4-oxadiazole-5-carboxamide as a white solid (34 mg, 74 μmol, 29%). ^1^H NMR (400 MHz, DMSO-*d*_6_): δ 9.54 (br s, 1H), 7.75–7.46 (m, 4H), 7.35 (br d, *J* = 6.2 Hz, 2H), 7.16 (br d, *J* = 7.5 Hz, 1H),5.68–5.26 (m, 1H), 4.70–4.27 (m, 3H), 4.04 (br s, 1H), 3.85 (br s, 6H), 3.56 (br s, 2H), 2.94 (br s, 2H). HRMS (ESI): calcd for [M+H]^+^ C_23_H_24_N_4_O_5_F, 455.1731, found 455.1744.

### *N*-(4-(3,3-Difluoroazetidine-1-carbonyl)phenethyl)-3-(3,4-dimethoxyphenyl)-1,2,4-oxadiazole-5-carboxamide (**17**)

Following general procedure C, *N*-(4-(3,3-difluoroazetidine-1-carbonyl)phenethyl)-3-(3,4-dimethoxyphenyl)-1,2,4-oxadiazole-5-carboxamide was obtained from 4-(2-(3-(3,4-dimethoxyphenyl)-1,2,4-oxadiazole-5-carboxamido)ethyl)benzoic acid (100 mg, 251 μmol, 1 equiv) and 3,3-difluoroazetidine (28 mg, 301 μmol, 1.2 equiv) after 12 h under stirring. After purification by prep-HPLC (water 0.225%FA–ACN]; B%: 30–60%, 9 min), *N*-(4-(3,3-difluoroazetidine-1-carbonyl)phenethyl)-3-(3,4-dimethoxyphenyl)-1,2,4-oxadiazole-5-carboxamide was obtained as a white solid (31 mg, 65 μmol, 26%). ^1^H NMR (400 MHz, DMSO-*d*_6_): δ 9.55 (t, *J* = 5.7 Hz, 1H), 7.69–7.61 (m, 3H), 7.53 (d, *J* = 2.0 Hz, 1H), 7.38 (d, *J* = 8.2 Hz, 2H),7.18 (d, *J* = 8.6 Hz, 1H), 4.88–4.33 (m, 4H), 3.86 (s, 6H), 3.61–3.54 (m, 2H), 2.96 (t, *J* = 7.3 Hz, 2H). HRMS (ESI): calcd for [M+H]^+^ C_23_H_22_N_4_O_5_F_2_, 473.1637, found 473.1647.

### *N*-(4-(2-Oxa-6-azaspiro[3.3]heptane-6-carbonyl)phenethyl)-3-(3,4-dimethoxyphenyl)-1,2,4-oxadiazole-5-carboxamide (**18**)

Following general procedure C, *N*-(4-(2-oxa-6-azaspiro[3.3]heptane-6-carbonyl)phenethyl)-3-(3,4-dimethoxyphenyl)-1,2,4-oxadiazole-5-carboxamide was obtained from 4-(2-(3-(3,4-dimethoxyphenyl)-1,2,4-oxadiazole-5-carboxamido)ethyl)benzoic acid (100 mg, 251 μmol, 1 equiv) and 2-oxa-6-azaspiro[3.3]heptane (29 mg, 301 μmol, 1.2 equiv) after 12 h under stirring. After purification by prep-HPLC (water 0.225% FA–ACN]; B%: 30–60%, 9 min), *N*-(4-(2-oxa-6-azaspiro[3.3]heptane-6-carbonyl)phenethyl)-3-(3,4-dimethoxyphenyl)-1,2,4-oxadiazole-5-carboxamide was obtained as a white solid (23 mg, 48 μmol, 19%). ^1^H NMR (400 MHz, DMSO-*d*_6_): δ 9.55 (t, *J* = 5.7 Hz, 1H), 7.66 (dd, *J* = 2.0, 8.3 Hz, 1H), 7.60–7.51 (m, 3H), 7.34 (d, *J* = 8.2 Hz,2H), 7.18 (d, *J* = 8.6 Hz, 1H), 4.67 (br d, *J* = 1.3 Hz, 4H), 4.46 (br s, 2H), 4.20 (br s, 2H), 3.86 (s, 6H), 3.61–3.52 (m, 2H), 2.95 (t, *J* = 7.3 Hz, 2H). LC-MS: *m*/*z* 479 [M+H]^+^.

### 3-(3,4-Dimethoxyphenyl)-*N*-[2-(3-methoxyphenyl)ethyl]-1,2,4-oxadiazole-5-carboxamide (**19**)

To a solution of 3-(3,4-dimethoxyphenyl)-*N*-(3-hydroxyphenethyl)-1,2,4-oxadiazole-5-carboxamide (90 mg, 0.24 mmol) in DMF (5 mL) were added potassium carbonate (118 mg, 0.85 mmol) and methyl iodide (42 μL, 0.68 mmol). The reaction mixture was stirred at rt for 16 h, concentrated *in vacuo*, dissolved in EtOAc (10 mL), washed with water (3 × 10 mL), passed through a hydrophobic frit, and concentrated *in vacuo*. Purification by prep-HPLC afforded 3-(3,4-dimethoxyphenyl)-*N*-[2-(3-methoxyphenyl)ethyl]-1,2,4-oxadiazole-5-carboxamide (21 mg, 6%) as a colorless oil. ^1^H NMR (400 MHz, DMSO-*d*_6_): δ 9.52 (s, 1H), 7.65 (dd, *J* = 8.4, 2.0 Hz, 1H), 7.52 (d, *J* = 2.0 Hz, 1H), 7.24–7.16 (m, 2H), 6.83–6.77 (m, 3H), 3.85 (s, 6H), 3.73 (s, 3H), 3.53 (t, *J* = 7.5 Hz, 2H), 2.86 (t, *J* = 7.4 Hz, 2H). HRMS (ESI): calcd for [M+H]^+^ C_20_H_22_N_3_O_5_, 384.1570, found 384.1557.

### Methyl 3-(3,4-Dihydroxyphenyl)-1,2,4-oxadiazole-5-carboxylate (**2b**)

To a solution of 3,4-dihydroxybenzonitrile (500 mg, 3.74 mmol) and hydroxylamine hydrochloride (386 mg, 5.5 mmol) in ethanol (5 mL) was added DIPEA (1 mL, 5.9 mmol). The reaction mixture was heated at 80 °C for 16 h, concentrated *in vacuo*, and then dissolved in DCM (5 mL). Triethylamine (1 mL, 7.4 mmol) was added, and the reaction mixture was cooled to 0 °C. Methyl 2-chloro-2-oxo-acetate (0.5 mL, 5.5 mmol) was added, and the reaction mixture was heated at 40 °C for 16 h, concentrated *in vacuo*, dissolved in water (10 mL), extracted with EtOAc (3 × 10 mL), passed through a hydrophobic frit, and concentrated *in vacuo*. Purification by flash column chromatography afforded methyl 3-(3,4-dihydroxyphenyl)-1,2,4-oxadiazole-5-carboxylate (527 mg, 60%) as a yellow solid. ^1^H NMR (400 MHz, DMSO-*d*_6_): δ 9.72 (s, 1H), 9.50 (s, 1H), 7.43 (d, *J* = 2.1 Hz, 1H), 7.40 (dd, *J* = 8.2, 2.1 Hz, 1H), 6.90 (d, *J* = 8.2 Hz, 1H), 3.98 (s, 3H).

### 3-(3,4-Dihydroxyphenyl)-*N*-[2-(3,4-dihydroxyphenyl)ethyl]-1,2,4-oxadiazole-5-carboxamide (**20**)

Following general procedure A, compound 3-(3,4-dihydroxyphenyl)-*N*-[2-(3,4-dihydroxyphenyl)ethyl]-1,2,4-oxadiazole-5-carboxamide was obtained from methyl 3-(3,4-dihydroxyphenyl)-1,2,4-oxadiazole-5-carboxylate (100 mg, 0.42 mmol), 4-(2-aminoethyl)benzene-1,2-diol hydrochloride (72 mg, 0.38 mmol), and triethylamine (148 μL, 1.06 mmol) in methanol (5 mL). Purification by prep-HPLC afforded 3-(3,4-dihydroxyphenyl)-*N*-[2-(3,4-dihydroxyphenyl)ethyl]-1,2,4-oxadiazole-5-carboxamide (11 mg, 7%) as an off-white solid. ^1^H NMR (500 MHz, DMSO-*d*_6_): δ 9.70 (s, 1H), 9.47 (s, 1H), 9.40 (t, *J* = 5.8 Hz, 1H), 8.76 (s, 1H), 8.66 (s, 1H), 7.43 (d, *J* = 2.1 Hz, 1H), 7.37 (dd, *J* = 8.2, 2.1 Hz, 1H), 6.90 (d, *J* = 8.2 Hz, 1H), 6.65–6.61 (m, 2H), 6.47 (dd, *J* = 8.0, 2.1 Hz, 1H), 3.44–3.40 (m, 2H), 2.68 (t, *J* = 7.6 Hz, 2H). HRMS (ESI): calcd for [M+H]^+^ C_17_H_16_N_3_O_6_, 358.1034, found 358.1028.

### *tert*-Butyl (2-(6-(Azetidine-1-carbonyl)pyridin-3-yl)ethyl)carbamate (**23**)

To a mixture of azetidin-1-yl-(5-bromopyridin-2-yl)methanone (400 mg, 1.66 mmol, 1 equiv) and potassium (2-((*tert*-butoxycarbonyl)amino)ethyl)trifluoroborate (624 mg, 2.49 mmol, 1.5 equiv) in a solution of toluene (3 mL) and H_2_O (1 mL) were added Pd(dppf)Cl_2_ (121 mg, 165.92 μmol, 0.1 equiv) and Cs_2_CO_3_ (1.35 g, 4.15 mmol, 2.5 equiv) in turns at 25 °C, and the resulting mixture was stirred at 100 °C for 12 h under N_2_. The residue was poured into water (20 mL). The aqueous phase was extracted with ethyl acetate (10 mL × 3). The combined organic phase was washed with brine (5 mL), dried with anhydrous Na_2_SO_4_, filtered, and concentrated *in vacuo*. The crude product was purified by reversed-phase HPLC (0.1% FA condition). *tert*-Butyl (2-(6-(azetidine-1-carbonyl)pyridin-3-yl)ethyl)carbamate (80 mg, crude, 15%) was obtained as a white solid. LC-MS: *m*/*z* 306 [M+H]^+^.

### (5-(2-Aminoethyl)pyridin-2-yl)(azetidin-1-yl)methanone (**24**)

To a mixture of *tert*-butyl (2-(6-(azetidine-1-carbonyl)pyridin-3-yl)ethyl)carbamate (80 mg, 261 μmol, 1 equiv) in DCM (2 mL) was added TFA (616 mg, 5.40 mmol, 400 μL, 20.62 equiv) in one portion at 0 °C. The mixture was stirred at 25 °C for 1 h and concentrated to get the residue. The residue was used in the next step without purification. (5-(2-Aminoethyl)pyridin-2-yl)(azetidin-1-yl)methanone (50 mg, crude, 93%) was obtained as a white oil. LC-MS: *m*/*z* 206 [M+H]^+^.

### *N*-(2-(6-(Azetidine-1-carbonyl)pyridin-3-yl)ethyl)-3-(3,4-dimethoxyphenyl)-1,2,4-oxadiazole-5-carboxamide (**25**)

To a mixture of (5-(2-aminoethyl)pyridin-2-yl)(azetidin-1-yl)methanone (48 mg, 237 μmol, 1.1 equiv) and ethyl 3-(3,4-dimethoxyphenyl)-1,2,4-oxadiazole-5-carboxylate (60 mg, 215 μmol, 1 equiv) in MeOH (1 mL) was added triethylamine (65 mg, 646 μmol, 90 μL, 3 equiv) 25 °C, and the resulting mixture was stirred at 60 °C for 12 h. The residue was poured into water (20 mL). The aqueous phase was extracted with ethyl acetate (10 mL × 3). The combined organic phase was washed with brine (5 mL), dried with anhydrous Na_2_SO_4_, filtered, and concentrated *in vacuo*. The residue was purified by prep-HPLC (water 0.05% ammonia hydroxide v/v–ACN; B%: 15–45%, 10 min). *N*-(2-(6-(azetidine-1-carbonyl)pyridin-3-yl)ethyl)-3-(3,4-dimethoxyphenyl)-1,2,4-oxadiazole-5-carboxamide (40 mg, 91 μmol, 42% yield) was obtained as a white solid. LC-MS: *m*/*z* 438.2 [M+H]^+^. ^1^H NMR: (400 MHz DMSO-*d*_6_): δ 8.68 (t, *J* = 5.8 Hz, 1H), 7.63 (d, *J* = 1.5 Hz, 1H), 7.05–6.93 (m, 2H), 6.78 (dd, *J* = 2.0, 8.4 Hz,1H), 6.65 (d, *J* = 2.0 Hz, 1H), 6.30 (d, *J* = 8.4 Hz, 1H), 3.68 (t, *J* = 7.7 Hz, 2H), 3.19 (t, *J* = 7.7 Hz, 2H), 2.98 (s, 6H), 2.73 (q, *J* = 6.8 Hz, 2H), 2.10 (t, *J* = 7.0 Hz, 2H), 1.39 (quin, *J* = 7.7 Hz, 2H).

### *tert*-Butyl (1-amino-3-(4-bromophenyl)-1-oxopropan-2-yl)carbamate (**27**)

To a solution of 3-(4-bromophenyl)-2-((*tert*-butoxycarbonyl)amino)propanoic acid (30 g, 87.16 mmol, 1 equiv), HATU (39.77 g, 104.59 mmol, 1.2 equiv), and DIPEA (33.79 g, 261.48 mmol, 45.54 mL, 3 equiv) in DCM (600 mL) was added NH_4_Cl (13.99 g, 261.48 mmol, 3 equiv). The mixture was stirred at 25 °C for 16 h, poured into H_2_O (500 mL), and extracted with EtOAc (700 mL × 3). The combined organic layer was washed with brine (500 mL), dried over Na_2_SO_4_, filtered, and concentrated. The residue was purified by column chromatography (SiO_2_, PE:EtOAc = 5:1 to 1:1). *tert*-butyl (1-amino-3-(4-bromophenyl)-1-oxopropan-2-yl)carbamate (30 g, crude, 100%) was obtained as a white solid. ^1^H NMR (400 MHz, DMSO-*d*_6_): δ 7.46 (d, *J* = 8.3 Hz, 2H), 7.38 (br s, 1H), 7.22 (br d, *J* = 8.3 Hz, 2H), 7.02 (br s, 1H), 6.83 (d, *J* = 8.7 Hz, 1H), 4.13–4.02 (m, 1H), 2.93 (dd, *J* = 4.3, 13.7 Hz, 1H), 2.75–2.69 (m, 1H), 1.29 (d, *J* = 9.5 Hz, 9H).

### Ethyl 4-(3-Amino-2-((*tert*-butoxycarbonyl)amino)-3-oxopropyl)benzoate (**28**)

To a solution of *tert*-butyl (1-amino-3-(4-bromophenyl)-1-oxopropan-2-yl)carbamate (25 g, 72.84 mmol, 1 equiv) in EtOH (250 mL) were added triethylamine (22.11 g, 218.52 mmol, 30.42 mL, 3 equiv) and Pd(dppf)Cl_2_ (5.33 g, 7.28 mmol, 0.1 equiv). The mixture was stirred at 80 °C under CO (72.84 mmol, 1 equiv) for 20 h, filtered, and concentrated. The residue was purified by column chromatography (SiO_2_, PE:EtOAc = 3:1 to 1:1). Ethyl 4-(3-amino-2-((*tert*-butoxycarbonyl)amino)-3-oxopropyl)benzoate (12 g, 35.67 mmol, 48%) was obtained as a brown solid. ^1^H NMR (400 MHz, DMSO-*d*_6_): δ 7.86 (d, *J* = 8.2 Hz, 2H), 7.40 (br d, *J* = 8.1 Hz, 3H), 7.03 (br s, 1H), 6.87 (d, *J* = 8.8 Hz, 1H), 4.29 (q, *J* = 7.1 Hz, 2H), 4.12 (dt, *J* = 4.5, 9.4 Hz, 1H), 3.03 (dd, *J* = 4.4, 13.7 Hz, 1H), 2.80 (br dd, *J* = 10.3, 13.5 Hz, 1H), 1.30–1.26 (m, 9H), 1.25–1.09 (m, 3H).

### 4-(3-Amino-2-((*tert*-butoxycarbonyl)amino)-3-oxopropyl)benzoic Acid (**29**)

To a solution of ethyl 4-(3-amino-2-((*tert*-butoxycarbonyl)amino)-3-oxopropyl)benzoate (12 g, 35.67 mmol, 1 equiv) in EtOH (120 mL) was added a solution of LiOH (2.56 g, 107.02 mmol, 3 equiv) in water (30 mL) at 25 °C. The mixture was stirred at 25 °C for 16 h, poured into water (150 mL),and diluted with EtOAc (200 mL), and the aqueous phase was acidified to pH 2 with HCl (1 N) and diluted with EtOAc (100 mL × 3). The combined organic layer was washed with brine (100 mL), dried over Na_2_SO_4_, filtered, and concentrated. 4-(3-Amino-2-((*tert*-butoxycarbonyl)amino)-3-oxopropyl)benzoic acid (5.5 g, 17.84 mmol, 50%) was obtained as a yellow solid. ^1^H NMR (400 MHz, DMSO-*d*_6_): δ 12.86–12.42 (m, 1H), 7.84 (br d, *J* = 8.2 Hz, 2H), 7.37 (br d, *J* = 8.3 Hz, 3H), 7.03 (br s, 1H), 6.86 (d, *J* = 8.8 Hz, 1H), 4.12 (br d, *J* = 3.3 Hz, 1H), 3.02 (br dd, *J* = 4.2, 13.8 Hz, 1H), 2.79 (br dd, *J* = 10.5, 13.3 Hz, 1H), 1.35–1.23 (m, 9H).

### 2-Amino-3-(4-(azetidine-1-carbonyl)phenyl)propanamide (**30**)

To a solution of 4-(3-amino-2-((*tert*-butoxycarbonyl)amino)-3-oxopropyl)benzoic acid (5.5 g, 17.84 mmol, 1 equiv) and azetidine (2.00 g, 21.41 mmol, 2.37 mL, 1.2 equiv, HCl) in DCM (60 mL) were added HATU (8.14 g, 21.41 mmol, 1.2 equiv) and DIPEA (6.92 g, 53.51 mmol, 9.32 mL, 3 equiv) in turns at 25 °C. The mixture was stirred at 25 °C for 16 h, poured into water (100 mL), and extracted with EtOAc (100 mL × 3). The combined organic layer was washed with brine (100 mL), dried over Na_2_SO_4_, filtered, and concentrated. The residue was purified by column chromatography (SiO_2_, DCM:MeOH = 20:1 to 10:1). *tert*-Butyl (1-amino-3-(4-(azetidine-1-carbonyl)phenyl)-1-oxopropan-2-yl)carbamate (3.2 g, 9.21 mmol, 51%) was obtained as a yellow solid. To a solution of *tert*-butyl (1-amino-3-(4-(azetidine-1-carbonyl)phenyl)-1-oxopropan-2-yl)carbamate (3.2 g, 9.21 mmol, 1 equiv) in DCM (32 mL) was added TFA (12.32 g, 108.05 mmol, 8 mL, 11.73 equiv). The mixture was stirred at 25 °C for 1 h. The mixture was poured into NaHCO_3_ (40 mL) and extracted with EtOAc (40 mL × 3). The combined organic layer was washed with brine (50 mL), dried over Na_2_SO_4_, filtered, and concentrated. The crude product was purified by reversed-phase HPLC (0.1% NH_3_·H_2_O). 2-Amino-3-(4-(azetidine-1-carbonyl)phenyl)propanamide (1.05 g, 4.25 mmol, 46%) was obtained as a yellow solid. ^1^H NMR (400 MHz, DMSO-*d*_6_): δ 7.53 (d, *J* = 8.1 Hz, 2H), 7.36 (br s, 1H), 7.29 (d, *J* = 8.1 Hz, 2H), 6.98 (br s, 1H), 4.29 (br s, 2H), 4.03 (br t, *J* = 6.8 Hz, 2H), 3.40–3.37 (m, 1H), 2.95 (br dd, *J* = 5.0, 13.3 Hz, 1H), 2.66 (br dd, *J* = 8.3, 13.4 Hz, 1H), 2.25 (br t, *J* = 7.6 Hz, 2H).

### *N*-(1-Amino-3-(4-(azetidine-1-carbonyl)phenyl)-1-oxopropan-2-yl)-3-(3,4-dimethoxyphenyl)-1,2,4-oxadiazole-5-carboxamide (**31**)

To a solution of 2-amino-3-(4-(azetidine-1-carbonyl)phenyl)propanamide (900 mg, 3.64 mmol, 1 equiv) and ethyl 3-(3,4-dimethoxyphenyl)-1,2,4-oxadiazole-5-carboxylate (911 mg, 3.28 mmol, 0.9 equiv) in MeOH (10 mL) was added Et_3_N (1.10 g, 10.92 mmol, 1.52 mL, 3 equiv). The mixture was stirred at 60 °C for 2 h, poured into H_2_O (15 mL), and extracted with EtOAc (20 mL × 3). The combined organic layer was washed with brine (20 mL), dried over Na_2_SO_4_, filtered, and concentrated. The residue was purified by prep-HPLC (water 0.225% FA–ACN; B%: 21–51%, 10 min) and lyophilized. *N*-(1-Amino-3-(4-(azetidine-1-carbonyl)phenyl)-1-oxopropan-2-yl)-3-(3,4-dimethoxyphenyl)-1,2,4-oxadiazole-5-carboxamide (70 mg, 145 μmol, 4%) was obtained as a yellow solid. ^1^H NMR (400 MHz, DMSO-*d*_6_): δ 9.47 (d, *J* = 8.4 Hz, 1H), 7.71 (s, 1H), 7.65 (dd, *J* = 2.0, 8.4 Hz, 1H), 7.52 (dd, *J* = 3.1, 5.1 Hz, 3H), 7.37 (d, *J* = 8.3 Hz, 2H), 7.30 (s, 1H), 7.17 (d, *J* = 8.6 Hz, 1H), 4.77–4.58 (m, 1H), 4.25 (br t, *J* = 7.4 Hz, 2H), 4.00 (br t, *J* = 7.5 Hz, 2H), 3.85 (d, *J* = 0.7 Hz, 6H), 3.25–3.21 (m, 1H), 3.15–3.06 (m, 1H), 2.22 (br t, *J* = 7.8 Hz, 2H).

### *N*-(2-(4-(Azetidine-1-carbonyl)phenyl)-1-cyanoethyl)-3-(3,4-dimethoxyphenyl)-1,2,4-oxadiazole-5-carboxamide (**33**)

To a solution of *N*-(1-amino-3-(4-(azetidine-1-carbonyl)phenyl)-1-oxopropan-2-yl)-3-(3,4-dimethoxyphenyl)-1,2,4-oxadiazole-5-carboxamide (70 mg, 145 μmol, 1 equiv) in THF (1 mL) were added triethylamine (44 mg, 437 μmol, 60 μL, 3 equiv) and TFAA (61 mg, 291 μmol, 40 μL, 2 equiv) in turns below 0 °C. The mixture was stirred at 25 °C under N_2_ for 0.5 h, poured into NaHCO_3_ (5 mL), and extracted with EtOAc (10 mL × 3). The combined organic layer was washed with brine (10 mL), dried over Na_2_SO_4_, filtered, and concentrated. The residue was purified by prep-TLC (SiO_2_, PE:EtOAc = 0:1) to give *N*-(2-(4-(azetidine-1-carbonyl)phenyl)-1-cyanoethyl)-3-(3,4-dimethoxyphenyl)-1,2,4-oxadiazole-5-carboxamide (17 mg, 36 μmol, 25%) as a pink solid. ^1^H NMR (500 MHz, DMSO): δ 10.35–10.32 (m, 1H), 7.68–7.65 (m, 1H), 7.60–7.53 (m, 3H), 7.45- 7.42 (m, 2H), 7.20–7.17 (m, 1H), 5.30–5.25 (m, 1H), 4.29–4.23 (m, 2H), 4.05–3.99 (m, 2H), 3.88–3.83 (m, 2H), 3.37–3.27 (m, 6H), 2.29–2.20 (m, 2H). HRMS (ESI): calcd for [M+H]^+^ C_24_H_24_N_5_O_5_, 462.1772, found 462.1790.

### Lithium 3-(3,4-Dimethoxyphenyl)-1,2,4-oxadiazole-5-carboxylic Acid (**35**)

To a solution of ethyl 3-(3,4-dimethoxyphenyl)-1,2,4-oxadiazole-5-carboxylate (500 mg, 1.80 mmol, 1.00 equiv) in MeOH (10.0 mL) was added a mixture of LiOH·H_2_O (226 mg, 5.39 mmol, 3.00 equiv) in water (3.00 mL) at 0 °C. The mixture was stirred at 50 °C for 2 h and concentrated *in vacuo*. Lithium 3-(3,4-dimethoxyphenyl)-1,2,4-oxadiazole-5-carboxylic acid (460 mg, crude) was obtained as a white solid.^1^H NMR (400 MHz, DMSO-*d*_6_): δ 7.60 (dd, *J* = 1.2, 8.4 Hz, 1H), 7.50 (d, *J* = 1.2 Hz, 1H), 7.12 (d, *J* = 8.4 Hz, 1H), 3.83 (d, *J* = 4.8 Hz, 6H).

### *N*-(1-Cyano-2-(4-(3-methoxyazetidine-1-carbonyl)phenyl)ethyl)-3-(3,4-dimethoxyphenyl)-1,2,4-oxadiazole-5-carboxamide (**34**)

To a solution of 4-(3-amino-2-((*tert*-butoxycarbonyl) amino)-3-oxopropyl) benzoic acid (500 mg, 1.39 mmol, 1.00 equiv), 3-methoxyazetidine (207 mg, 1.67 mmol, 1.20 equiv, HCl), and HATU (795 mg, 2.09 mmol, 1.50 equiv) in DMF (6 mL) was added DIPEA (541 mg, 4.18 mmol, 729 μL, 3.00 equiv). The mixture was stirred at 20 °C for 5 h, poured into water (8 mL), and extracted with EtOAc (10 mL × 3). The combined organic layer was washed with brine (15 mL), dried over Na_2_SO_4_, filtered, and concentrated. The residue was purified by column chromatography (SiO_2_, PE:EtOAc = 3:1 to 0:1). Impure *tert*-butyl(1-amino-3-(4-(3-methoxyazetidine-1-carbonyl)phenyl)-1-oxopropan-2-yl)carbamate (420 mg, 1.00 mmol, 71% yield) was obtained as a white solid. To a solution of *tert*-butyl (1-amino-3-(4-(3-methoxyazetidine-1-carbonyl)phenyl)-1-oxopropan-2-yl)carbamate (200 mg, 530 μmol, 1.00 equiv) in DCM (2 mL) was added TFA (308 mg, 2.70 mmol, 0.20 mL, 5.10 equiv). The mixture was stirred at 20 °C for 1 h and concentrated *in vacuo*. The crude product was used for next step directly. 2-Amino-3-(4-(3-methoxyazetidine-1-carbonyl)phenyl)propanamide (230 mg, crude, TFA, 117%) was obtained as yellow oil. To a solution of 2-amino-3-(4-(3-methoxyazetidine-1-carbonyl)phenyl)propanamide (100 mg, 255 μmol, 1.00 equiv, TFA) in DMF (1 mL) were added HATU (146 mg, 383 μmol, 1.50 equiv) and lithium 3-(3,4-dimethoxyphenyl)-1,2,4-oxadiazole-5-carboxylic acid (64 mg, 255 μmol, 1.00 equiv) at 0 °C. Then DIPEA (99.1 mg, 767 μmol, 134 μL, 3.00 equiv) was added to the mixture at 0 °C and stirred at 20 °C for 12 h. The mixture was poured into water (10 mL) and extracted with EtOAc (10 mL × 3). The combined organic layer was washed with brine (20 mL), dried over Na_2_SO_4_, filtered, and concentrated. The residue was purified by prep-HPLC (column: Shim-pack C18 150 × 25 × 10 μm; mobile phase: [water (0.225%FA)–ACN]; B%: 20–50%, 10 min) and lyophilized. *N*-(1-Amino-3-(4-(3-methoxyazetidine-1-carbonyl)phenyl)-1-oxopropan-2-yl)-3-(3,4-dimethoxyphenyl)-1,2,4-oxadiazole-5-carboxamide impure (35 mg, 68 μmol, 13%) was obtained as a white solid. To a solution of *N*-(1-amino-3-(4-(3-methoxyazetidine-1-carbonyl)phenyl)-1-oxopropan-2-yl)-3-(3,4-dimethoxyphenyl)-1,2,4-oxadiazole-5-carboxamide (35 mg, 68 μmol, 1.00 equiv) in THF (0.20 mL) were added triethylamine (20 mg, 206 μmol, 28 μL, 3.00 equiv) and TFAA (28 mg, 138 μmol, 19 μL, 2.00 equiv) at 0 °C under N_2_. The mixture was stirred at 20 °C for 0.5 h, poured into water (3 mL), and extracted with EtOAc (5 mL × 3). The combined organic layer was washed with brine (10 mL), dried over Na_2_SO_4_, filtered, and concentrated. The residue was purified by prep-HPLC (column: Waters Xbridge 150× 25 mm × 5 μm; mobile phase: [water (10 mM NH_4_HCO_3_)–ACN]; B%: 20–50%, 10 min). *N*-(1-Cyano-2-(4-(3-methoxyazetidine-1-carbonyl)phenyl)ethyl)-3-(3,4-dimethoxyphenyl)-1,2,4-oxadiazole-5-carboxamide (5 mg, 11 μmol, 16%) was obtained as a yellow solid. ^1^H NMR (500 MHz, DMSO): δ 10.00 (br s, 1H), 7.69–7.66 (m, 1H), 7.62–7.53 (m, 3H), 7.46–7.43 (m, 2H), 7.21–7.18 (m, 1H), 5.29 (t, *J* = 7.9 Hz, 1H), 4.44–4.39 (m, 1H), 4.26–4.17 (m, 2H), 4.13–4.07 (m, 1H), 3.90–3.78 (m, 7H), 3.37–3.31 (m, 2H), 3.21 (s, 3H). HRMS (ESI): calcd for [M+H]^+^ C_25_H_26_N_5_O_6_, 492.1878, found 492.1882.

### *N*-(1-Amino-1-oxo-3-phenylpropan-2-yl)-3-(3,4-dimethoxyphenyl)-1,2,4-oxadiazole-5-carboxamide (**37**)

To a solution of ethyl 3-(3,4-dimethoxyphenyl)-1,2,4-oxadiazole-5-carboxylate (2.50 g, 8.97 mmol, 0.9 equiv) in MeOH (25 mL) were added 2-amino-3-phenylpropanamide (2.00 g, 9.97 mmol, 1 equiv) and triethylamine (4.03 g, 39.87 mmol, 5.55 mL, 4 equiv). The mixture was stirred at 60 °C for 16 h, poured into water (40 mL), and extracted with EtOAc (50 mL × 3). The combined organic layers were washed with brine (50 mL), dried over Na_2_SO_4_, filtered, and concentrated. The residue was washed with 20 mL (PE:EtOAc = 3:1) and filtered to give *N*-(1-amino-1-oxo-3-phenylpropan-2-yl)-3-(3,4-dimethoxyphenyl)-1,2,4-oxadiazole-5-carboxamide (1 g, 2.52 mmol, 25%) as a gray solid. ^1^H NMR (400 MHz, DMSO-*d*_6_): δ 9.42 (br d, *J* = 6.7 Hz, 1H), 7.61–7.73 (m, 2H), 7.52 (d, *J* = 2.0 Hz, 1H), 7.24–7.31 (m, 5H), 7.15–7.21 (m, 3H), 4.65 (br s, 1H), 3.85 (d, *J* = 1.6 Hz, 6H), 3.16–3.25 (m, 1H), 3.06 (dd, *J* = 13.9, 10.2 Hz, 1H).

### *N*-(1-Cyano-2-phenylethyl)-3-(3,4-dimethoxyphenyl)-1,2,4-oxadiazole-5-carboxamide (**38**)

To a solution of *N*-(1-amino-1-oxo-3-phenylpropan-2-yl)-3-(3,4-dimethoxyphenyl)-1,2,4-oxadiazole-5-carboxamide (100 mg, 252 μmol, 1 equiv) in THF (2 mL) were added triethylamine (76 mg, 756 μmol, 105 μL, 3 equiv) and TFAA (105 mg, 504 μmol, 70 μL, 2 equiv) at 0 °C. The mixture was stirred at 25 °C for 0.5 h, poured into saturated NaHCO_3_ aqueous solution (5 mL), and extracted with EtOAc (10 mL × 3). The combined organic layer was washed with brine (20 mL), dried over Na_2_SO_4_, filtered, and concentrated. The residue was purified by prep-TLC (SiO_2_, PE:EtOAc = 3:1) to give *N*-(1-cyano-2-phenylethyl)-3-(3,4-dimethoxyphenyl)-1,2,4-oxadiazole-5-carboxamide (47 mg, 122 μmol, 48%) as a white solid. ^1^H NMR (400 MHz, DMSO-*d*_6_): δ 10.35 (br s, 1H), 7.67 (dd, *J* = 8.4, 2.0 Hz, 1H), 7.53 (d, *J* = 1.8 Hz, 1H), 7.30–7.40 (m, 4H), 7.23–7.29 (m, 1H), 7.18 (d, *J* = 8.4 Hz, 1H), 5.22 (t, *J* = 8.0 Hz, 1H), 3.86 (s, 6H), 3.27 (br d, *J* = 8.1 Hz, 2H). HRMS (ESI): calcd for [M+H]^+^ C_20_H_19_N_4_O_4_, 379.1401, found 379.1391.

### (4-(Chloromethyl)phenyl)(2-oxa-6-azaspiro[3.3]heptan-6-yl)methanone (**40**)

4-(Chloromethyl)benzoyl chloride (3.32 g,17.6 mmol) and DMAP (10 mg, 0.08 mmol) were dissolved in DCM (30 mL) under N_2_ and cooled in an ice bath. Triethylamine (2.45 mL,17.6 mmol) was added dropwise, followed by 2-oxa-6-azaspiro[3.3]heptane (1.74 g, 17.6 mmol), and the reaction mixture was stirred with cooling for 1 h, then a further hour at room temperature. The mixture was treated with water (30 mL) and extracted with DCM (3 × 30 mL). The combined organics were dried (MgSO_4_), filtered, evaporated, and purified on silica, eluting with 100:0 to 99:1 DCM/MeOH. (4-(Chloromethyl)phenyl)(2-oxa-6-azaspiro[3.3]heptan-6-yl)methanone was obtained as a white solid (3.53 g, 79%). ^1^H NMR (500 MHz, CDCl_3_): δ 7.66–7.63 (m, 2H), 7.48–7.45 (m, 2H), 4.88–4.79 (m, 4H), 4.63 (s, 2H), 4.45–4.39 (m, 4H). LC-MS: *m*/*z* 252 [M+H]^+^.

### 3-(4-(2-Oxa-6-azaspiro[3.3]heptane-6-carbonyl)phenyl)-2-((diphenylmethylene)amino)propanenitrile (**41**)

2-(Benzhydrylideneamino)acetonitrile (3.06 g, 13.9 mmol) and [4-(chloromethyl)phenyl]-(2-oxa-6-azaspiro[3.3]heptan-6-yl)methanone (3.56 g, 14.1 mmol) were dissolved in DCM (30 mL). Sodium hydroxide (11 M solution, 16 mL, 176 mmol) was added dropwise, followed by benzyltriethylammonium chloride (317 mg,1.39 mmol). The reaction mixture was stirred vigorously for 1 h, diluted with water (30 mL), and extracted with DCM (3 × 50 mL). The combined organics were dried (MgSO_4_), filtered, evaporated, and purified on silica, eluting with 100:0 to 0:100 heptane/EtOAc to give 3-(4-(2-oxa-6-azaspiro[3.3]heptane-6-carbonyl)phenyl)-2-((diphenylmethylene)amino)propanenitrile (5.21 g, 86%) as a yellow foam. ^1^H NMR (500 MHz, CDCl_3_): δ 7.64–7.53 (m, 4H), 7.50–7.45 (m, 4H), 7.38 (t, *J* = 7.6 Hz, 2H), 7.21–7.18 (m, 2H), 6.91 (d, *J* = 6.6 Hz, 2H), 4.82 (s, 4H), 4.46–4.33 (m, 5H), 3.33–3.23 (m, 2H).

### 3-(4-(2-Oxa-6-azaspiro[3.3]heptane-6-carbonyl)phenyl)-2-aminopropanenitrile (**42**)

3-(4-(2-Oxa-6-azaspiro[3.3]heptane-6-carbonyl)phenyl)-2-((diphenylmethylene)amino)propanenitrile (5.21 g, 12.0 mmol) was dissolved in THF (52 mL), and hydrogen chloride (1M, 12.6 mL, 12.6 mmol) was added dropwise. After stirring for 1 h, the THF was removed *in vacuo*, and the residue was treated with water (50 mL) and extracted with DCM (3 × 50 mL). The combined organics were dried (MgSO_4_), filtered, and evaporated. The residue was triturated with heptane, and the resulting white solid was filtered and dried *in vacuo* over the weekend to give impure 3-(4-(2-oxa-6-azaspiro[3.3]heptane-6-carbonyl)phenyl)-2-aminopropanenitrile (1.57 g, 48%). ^1^H NMR (500 MHz, CDCl_3_): δ 7.66–7.63 (m, 2H), 7.40–7.37 (m, 2H), 4.84 (s, 4H), 4.47–4.45 (m, 4H), 4.39 (s, 4H), 4.02–3.92 (m, 1H), 3.11–3.07 (m, 2H). LC-MS: *m*/*z* 272 [M+H]^+^.

### Sodium 3-(3,4-Dimethoxyphenyl)-1,2,4-oxadiazole-5-carboxylate (**43**)

Ethyl 3-(3,4-dimethoxyphenyl)-1,2,4-oxadiazole-5-carboxylate (10.76 g, 38.7 mmol) was suspended in ethanol (220 mL), and sodium hydroxide (1 N, 61 mL, 61 mmol) was added dropwise (exotherm to 28 °C). The reaction mixture was stirred at room temperature for 105 min and then evaporated, and the residual white solid was azeotroped with toluene (×3) and then dried overnight *in vacuo* at 50 °C to give sodium 3-(3,4-dimethoxyphenyl)-1,2,4-oxadiazole-5-carboxylate (11.4 g, 100%). ^1^H NMR (500 MHz, DMSO): δ 7.60 (dd, *J* = 2.0, 8.4 Hz, 1H), 7.51 (d, *J* = 2.0 Hz, 1H), 7.14–7.11 (m, 1H), 3.86–3.84 (m, 6H).

### *N*-(2-(4-(2-Oxa-6-azaspiro[3.3]heptane-6-carbonyl)phenyl)-1-cyanoethyl)-3-(3,4-dimethoxyphenyl)-1,2,4-oxadiazole-5-carboxamide (**44**)

Sodium 3-(3,4-dimethoxyphenyl)-1,2,4-oxadiazole-5-carboxylate (1.69 g, 6.21 mmol), 3-(4-(2-oxa-6-azaspiro[3.3]heptane-6-carbonyl)phenyl)-2-aminopropanenitrile (1.57 g, 5.79 mmol), and HATU (4.4 g, 11.6 mmol) were partially dissolved in DMF (30 mL) under N_2_, and DIPEA (2.02 mL,14.8 mmol) was added dropwise. The mixture was stirred at room temperature for 75 min. The bulk of the DMF was removed *in vacuo*, and the residue was treated with water (75 mL) and extracted with EtOAc (3 × 200 mL). The combined organics were washed (brine), dried (Na_2_SO_4_), filtered, and evaporated. The residue was stirred with warm EtOAc and loaded onto a pad of silica. The pad was washed with EtOAc (3 column volumes) then 9:1 EtOAc/acetone to elute the product. The filtrate was evaporated, and the residue was triturated with EtOAc, filtered, and purified on silica, eluting with 100:0 to 0:100 heptane/EtOAc. The product-containing cleanest fractions were combined and evaporated to a give a white foam and dried over the weekend at 50 °C. The foam was dissolved in DCM, evaporated to a white foam, and then dried at 50 °C under high vacuum for 2 h. NMR showed a trace of EtOAc and some DCM. The foam was redissolved in ACN and evaporated then dried under high vacuum at 50 °C to give *N*-(2-(4-(2-oxa-6-azaspiro[3.3]heptane-6-carbonyl)phenyl)-1-cyanoethyl)-3-(3,4-dimethoxyphenyl)-1,2,4-oxadiazole-5-carboxamide (410 mg, 13%). ^1^H NMR (500 MHz, DMSO): δ 10.35 (d, *J* = 7.9 Hz, 1H), 7.69–7.67 (m, 1H), 7.60–7.53 (m, 3H), 7.46–7.43 (m, 2H), 7.21–7.18 (m, 1H), 5.29 (q, *J* = 7.9 Hz, 1H), 4.66 (d, *J* = 4.3 Hz, 4H), 4.47–4.40 (m, 2H), 4.23–4.17 (m, 2H), 3.87–3.86 (m, 6H), 3.37–3.30 (m, 2H). ^13^C NMR (125 MHz, DMSO): δ 168.9, 168.5, 168.4, 153.3, 152.4, 149.6, 138.7, 132.5, 129.8, 128.3, 121.3, 118.4, 118.0, 112.5, 110.2, 80.0, 62.3, 58.2, 56.2, 56.1, 42.2, 38.2, 37.2. HRMS (ESI): calcd for [M+H]^+^ C_26_H_26_N_5_O_6_, 504.1883, found 504.1865.

### Ethyl 3-(3-Hydroxy-4-methoxyphenyl)-1,2,4-oxadiazole-5-carboxylate (**47**)

To a solution of (*Z*)-*N*′,3-dihydroxy-4-methoxybenzimidamide (2.00 g, 11.0 mmol, 1.00 equiv) and DIPEA (2.13 g, 16.5 mmol, 2.87 mL, 1.50 equiv) in THF (30.0 mL) was added ethyl 2-chloro-2-oxoacetate (1.50 g, 11.0 mmol, 1.23 mL, 1.00 equiv) at 0 °C. The mixture was stirred at 80 °C for 2 h and then concentrated *in vacuo*. The crude product was purified by column chromatography (SiO_2_, PE:EtOAc = 10:1 to 2:1). The eluant was concentrated *in vacuo* to give 2.38 g of yellow solid. Impure ethyl 3-(3-hydroxy-4-methoxyphenyl)-1,2,4-oxadiazole-5-carboxylate (2.38 g, 6.81 mmol, 62% yield, 76% purity) was obtained as a yellow solid. ^1^H NMR (400 MHz, CDCl_3_): δ 7.75–7.64 (m, 2H), 6.99–6.91 (m, 1H), 5.77 (br s, 1H), 4.57 (q, *J* = 7.2 Hz, 2H), 3.97 (s, 3H), 1.49 (t, *J* = 7.2 Hz, 3H). LC-MS: *m*/*z* 265 [M+H]^+^.

### Ethyl 3-(3-((1-Hydroxycyclopropyl)methoxy)-4-methoxyphenyl)-1,2,4-oxadiazole-5-carboxylate (**48**)

To a solution of ethyl 3-(3-hydroxy-4-methoxy-phenyl)-1,2,4-oxadiazole-5-carboxylate (1.2 g, 4.54 mmol, 1 equiv) and (1-tetrahydropyran-2-yloxycyclopropyl)methanol (860 mg, 5.00 mmol, 1.1 equiv) in THF (12 mL) were added PPh_3_ (1.43 g, 5.45 mmol, 1.2 equiv) and DEAD (949 mg, 5.45 mmol, 990 μL, 1.2 equiv) at 0 °C under N_2_. The mixture was stirred at 25 °C for 2 h and then concentrated. The crude product was purified by prep-HPLC (column: Phenomenex Luna C18 150 × 40 mm × 15 μm; mobile phase: [water (0.225%FA)–ACN];B%: 38–68%, 9 min). The eluent was concentrated and lyophilized. Impure ethyl 3-[3-[(1-hydroxycyclopropyl)methoxy]-4-methoxy-phenyl]-1,2,4-oxadiazole-5-carboxylate was obtained (950 mg, 1.28 mmol, 28% yield, 45.2% purity). LC-MS: *m*/*z* 335 [M+H]^+^.

### [4-(Chloromethyl)phenyl]-(3- methoxyazetidin-1-yl)methanone (**49**)

To a solution of 4-(chloromethyl)benzoyl chloride (3.00 g, 15.9 mmol, 1.00 equiv) and 3-methoxyazetidine (1.96 g, 15.9 mmol, 1.00 equiv, HCl) in DCM (40.0 mL) was added Et_3_N (4.01 g, 39.7 mmol, 5.52 mL, 2.50 equiv) at 0 °C. The mixture was stirred at 0 °C for 2 h, diluted with DCM (100 mL), washed with sat. NH_4_Cl (30 mL × 2) and brine (20 mL), dried over Na_2_SO_4_, filtered, and concentrated *in vacuo*. [4-(Chloromethyl)phenyl]-(3- methoxyazetidin-1-yl)methanone (3.8 g, 15.85 mmol, 99%) was obtained as a colorless oil. ^1^H NMR (400 MHz, CDCl_3_): δ 7.66–7.58 (m, 2H), 7.44 (d, *J* = 8.2 Hz, 2H), 4.60 (s, 2H), 4.42–4.34 (m, 2H), 4.29–4.22 (m, 1H), 4.17 (d, *J* = 6.4 Hz, 1H), 4.08 (d, *J* = 9.2 Hz, 1H), 3.32 (s, 3H).

### *N*-[1-Cyano-2-[4-(3-methoxyazetidine-1-carbonyl)phenyl]ethyl]-3-[3-[(1-hydroxycyclopropyl)methoxy]-4-methoxyphenyl]-1,2,4-oxadiazole-5-carboxamide (**45**)

To a solution of 2-(benzhydrylideneamino)acetonitrile (3.67 g, 16.6 mmol, 1.05 equiv) in THF (40.0 mL) was added NaOH (666 mg, 16.6 mmol, 1.05 equiv) at 20 °C. The mixture was stirred at 20 °C for 30 min. A solution of [4-(chloromethyl)phenyl]-(3-methoxyazetidin-1-yl)methanone (3.80 g, 15.8 mmol, 1.00 equiv) in THF (10.0 mL) was added to the mixture at 0 °C. The mixture was stirred at 20 °C for 2 h and then filtered. The filter cake was washed with EtOAc (10.0 mL × 3). The combined filtrate was concentrated *in vacuo* to give a residue. The residue was purified by silica gel chromatography (PE:EtOAc = 5:1–0:1). Impure 2-(benzhydrylideneamino)-3-[4-(3-methoxyazetidine-1-carbonyl)phenyl]propanenitrile (5.80 g, 13.7 mmol, 86%) was obtained as yellow oil. To a solution of 2-(benzhydrylideneamino)-3-[4-(3-methoxyazetidine-1-carbonyl)phenyl]propanenitrile (3.00 g, 7.08 mmol, 1.00 equiv) in dioxane (30.0 mL) was added HCl (1 M, 7.79 mL, 1.10 equiv) (1 M in water) at 20 °C. The mixture was stirred at 20 °C for 3 h, poured into water (100 mL), and then extracted with EtOAc (100 mL × 2). The aqueous phase was basified with sat. NaHCO_3_ adjusted to pH 12 and then extracted with DCM:MeOH = 10:1 (100 mL × 4). The combined organic layers were dried over Na_2_SO_4_, filtered, and concentrated *in vacuo*. Impure 2-amino-3-[4-(3-methoxyazetidine-1-carbonyl) phenyl]propanenitrile (1.4 g, 5.40 mmol, 76%) was obtained as a yellow oil. To a solution of 2-amino-3-[4-(3-methoxyazetidine-1-carbonyl)phenyl]propanenitrile (500 mg, 1.93 mmol, 1.00 equiv) and ethyl 3-[3-[(1-hydroxycyclopropyl)methoxy]-4-methoxy-phenyl]-1,2,4-oxadiazole-5-carboxylate (773 mg, 2.31 mmol, 1.20 equiv) in MeOH (10.0 mL) was added Et_3_N (390 mg, 3.86 mmol, 537 μL, 2.00 equiv) at 20 °C. The mixture was stirred at 40 °C for 16 h and then concentrated *in vacuo* to give a residue. The residue was purified by Prep-HPLC (water (0.225%FA)–ACN; B%: 30–60%, 10 min). *N*-[1-Cyano-2-[4-(3-methoxyazetidine-1-carbonyl)phenyl]ethyl]-3-[3-[(1-hydroxycyclopropyl)methoxy]-4-methoxy-phenyl]-1,2,4-oxadiazole-5-carboxamide (32 mg, 58 μmol, 3.04%) was obtained as a white solid. ^1^H NMR (500 MHz, DMSO): δ 10.38–10.32 (m, 1H), 7.67 (dd, *J* = 1.9, 8.5 Hz, 1H), 7.61–7.57 (m, 3H), 7.45–7.42 (m, 2H), 7.21–7.18 (m, 1H), 5.55 (s, 1H), 5.28 (t, *J* = 7.9 Hz, 1H), 4.43–4.36 (m, 1H), 4.26–4.18 (m, 2H), 4.13–4.02 (m, 3H), 3.90–3.78 (m, 4H), 3.37–3.31 (m, 2H), 3.25 (s, 3H), 0.76–0.68 (m, 4H). LC-MS: *m*/*z* 548 [M+H]^+^.

### 3-(3,4-Dimethoxyphenyl)-*N*-[2-(3,4-dimethoxyphenyl)ethyl]-*N*-methyl-1,2,4-oxadiazole-5-carboxamide (**51**)

To a solution of 3-(3,4-dimethoxyphenyl)-*N*-[2-(3,4-dimethoxyphenyl)ethyl]-1,2,4-oxadiazole-5-carboxamide (16 mg, 0.04 mmol) in DMF (5 mL) was added 60% sodium hydride (1.7 mg, 0.04 mmol), and the reaction mixture was stirred at rt for 10 min. Methyl iodide (3 μL, 0.04 mmol) was added, and the reaction mixture was stirred at rt for 16 h, concentrated *in vacuo*, dissolved in EtOAc (10 mL), washed with water (5 × 10 mL), passed through a hydrophobic frit, and concentrated *in vacuo*. Purification by prep-HPLC afforded 3-(3,4-dimethoxyphenyl)-*N*-[2-(3,4-dimethoxyphenyl)ethyl]-*N*-methyl-1,2,4-oxadiazole-5-carboxamide (11 mg, 63%) as a colorless oil. ^1^H NMR (500 MHz, DMSO-*d*_6_): δ 7.65–7.61 (m, 1H), 7.51–7.49 (m, 1H), 7.16 (d, *J* = 8.4 Hz, 1H), 6.89–6.62 (m, 3H), 3.86–3.81 (m, 7H), 3.78 (s, 1H), 3.74–3.67 (m, 4H), 3.58 (s, 2H), 3.15–3.10 (m, 3H), 2.90–2.85 (m, 2H). HRMS (ESI): calcd for [M+H]^+^ C_22_H_26_N_3_O_6_, 428.1822, found 428.1812.

### *N*-[[3-(3,4-Dimethoxyphenyl)-1,2,4-oxadiazol-5-yl]methyl]-2-phenyl-ethanamine (**53**)

To a solution of 5-(chloromethyl)-3-(3,4-dimethoxyphenyl)-1,2,4-oxadiazole (63 mg, 0.23 mmol) and 2-phenylethanamine (42 mg, 0.35 mmol) in DCM (5 mL) was added triethylamine (136 μL, 0.97 mmol). The reaction mixture was stirred at 40 °C for 16 h, diluted with DCM (10 mL), washed with water (3 × 10 mL), passed through a hydrophobic frit, and concentrated *in vacuo*. Purification by prep-HPLC afforded *N*-[[3-(3,4-dimethoxyphenyl)-1,2,4-oxadiazol-5-yl]methyl]-2-phenyl-ethanamine (43 mg, 31%) as a yellow oil. ^1^H NMR (500 MHz, DMSO-*d*_6_): δ 7.59 (dd, *J* = 8.4, 2.0 Hz, 1H), 7.48 (d, *J* = 2.0 Hz, 1H), 7.29–7.26 (m, 2H), 7.23–7.21 (m, 2H), 7.19–7.16 (m, 2H), 7.13 (d, *J* = 8.5 Hz, 1H), 4.07 (d, *J* = 6.3 Hz, 2H), 3.83 (s, 6H), 2.86–2.82 (m, 2H), 2.75–2.72 (m, 2H). LC-MS: *m*/*z* 340 [M+H]^+^.

### *N*-(4-(Azetidine-1-carbonyl)phenethyl)-3-(3-fluoro-4-methoxyphenyl)-1,2,4-oxadiazole-5-carboxamide (**61**)

To a solution of (*Z*)-3-fluoro-*N*′-hydroxy-4-methoxybenzimidamide (220 mg, 1.19 mmol, 1 equiv) and DIPEA (463 mg, 3.58 mmol, 624 μL, 3 equiv) in THF (3 mL) was added ethyl 2-chloro-2-oxo-acetate (163 mg, 1.19 mmol, 133 μL, 1 equiv) at 0 °C. The mixture was stirred at 25 °C for 0.5 h and then at 80 °C for 2 h, poured into water (5 mL), and extracted with EtOAc (10 mL × 3). The combined organic layer was washed with brine (10 mL), dried over Na_2_SO_4_, filtered, and concentrated. Ethyl 3-(3-fluoro-4-methoxyphenyl)-1,2,4-oxadiazole-5-carboxylate (200 mg, crude, 63%) was obtained as brown solid. To a solution of ethyl 3-(3-fluoro-4-methoxyphenyl)-1,2,4-oxadiazole-5-carboxylate (100 mg, 375 μmol, 1 equiv) in MeOH (2 mL) were added [4-(2-aminoethyl)phenyl]-(azetidin-1-yl)methanone (92 mg, 450 μmol, 1.2 equiv) and triethylamine (114 mg, 1.13 mmol, 156 μL, 3 equiv). The mixture was stirred at 60 °C for 16 h, poured into water (5 mL), and extracted with EtOAc (10 mL × 3). The combined organic layer was washed with brine (10 mL), dried over Na_2_SO_4_, filtered, and concentrated. The crude product was washed with MeOH (10 mL) and filtered to give *N*-(4-(azetidine-1-carbonyl)phenethyl)-3-(3-fluoro-4-methoxyphenyl)-1,2,4-oxadiazole-5-carboxamide (34 mg, 80 μmol, 21%) as a gray solid. ^1^H NMR (400 MHz, DMSO): δ 9.54 (br s, 1H), 7.91–7.76 (m, 2H), 7.56 (br d, *J* = 6.7 Hz, 2H), 7.41 (t, *J* = 8.4 Hz, 1H), 7.33 (br d, *J* = 7.0 Hz, 2H), 4.28 (br s, 2H), 4.02 (br s, 2H), 3.94 (s, 3H), 3.55 (q, *J* = 6.5 Hz, 2H), 2.93 (br t, *J* = 7.3 Hz, 2H), 2.27–2.20 (m, 2H). LC-MS: *m*/*z* 425 [M+H]^+^.

### *N*-(4-(Azetidine-1-carbonyl)phenethyl)-3-(4-fluoro-3-methoxyphenyl)-1,2,4-oxadiazole-5-carboxamide (**62**)

To a solution of (*Z*)-4-fluoro-*N*′-hydroxy-3-methoxybenzimidamide (230 mg, 1.25 mmol, 1 equiv) and DIPEA (484 mg, 3.75 mmol, 652 μL, 3 equiv) in THF (3 mL) was added ethyl 2-chloro-2-oxo-acetate (170 mg, 1.25 mmol, 139 μL, 1 equiv) at 0 °C. The mixture was stirred at 25 °C for 0.5 h and then stirred at 80 °C for 2 h, poured into water (5 mL), and extracted with EtOAc (10 mL × 3). The combined organic layer was washed with brine (10 mL), dried over Na_2_SO_4_, filtered, and concentrated. Ethyl 3-(4-fluoro-3-methoxyphenyl)-1,2,4-oxadiazole-5-carboxylate (200 mg, crude, 63%) was obtained as a brown oil. To a solution of ethyl 3-(4-fluoro-3-methoxyphenyl)-1,2,4-oxadiazole-5-carboxylate (100 mg, 375 μmol, 1 equiv) and [4-(2-aminoethyl)phenyl]-(azetidin-1-yl)methanone (92 mg, 450 μmol, 1.2 equiv) in MeOH (2 mL) was added triethylamine (114 mg, 1.13 mmol, 156 μL, 3 equiv). The mixture was stirred at 60 °C for 16 h, poured into water (5 mL), and extracted with EtOAc (10 mL × 3). The combined organic layer was washed with brine (10 mL), dried over Na_2_SO_4_, filtered, and concentrated. The residue was purified by prep-TLC (SiO_2_, PE:EtOAc = 3:1). *N*-(4-(azetidine-1-carbonyl)phenethyl)-3-(4-fluoro-3-methoxyphenyl)-1,2,4-oxadiazole-5-carboxamide (11 mg, 27 μmol, 7%) was obtained as a white solid. ^1^H NMR (400 MHz, DMSO): δ 9.58 (br t, *J* = 5.7 Hz, 1H), 7.73 (dd, *J* = 8.1, 1.7 Hz, 1H), 7.66 (ddd, *J* = 8.3, 4.2, 1.9 Hz, 1H), 7.56 (d, *J* = 8.1 Hz, 2H), 7.46 (dd, *J* = 11.2, 8.4 Hz, 1H), 7.33 (d, *J* = 8.1 Hz, 2H), 4.27 (br d, *J* = 7.1 Hz, 2H), 4.02 (br t, *J* = 7.4 Hz, 2H), 3.95 (s, 3H), 3.56 (q, *J* = 6.8 Hz, 2H), 2.94 (t, *J* = 7.4 Hz, 2H), 2.24 (quin, *J* = 7.7 Hz, 2H). LC-MS: *m*/*z* 425 [M+H]^+^.

### *N*-(4-(Azetidine-1-carbonyl)phenethyl)-3-(1*H*-benzo[*d*]imidazol-6-yl)-1,2,4-oxadiazole-5-carboxamide (**63**)

To a mixture of (*Z*)-*N*′-hydroxy-1*H*-benzo[*d*]imidazole-6-carboximidamide (300 mg, 1.70 mmol, 1 equiv) and DIPEA (440 mg, 3.41 mmol, 593 μL, 2 equiv) in THF (3 mL) was dropwise added ethyl 2-chloro-2-oxo-acetate (279 mg, 2.04 mmol, 228 μL, 1.2 equiv) at 0 °C, then the solution was stirred at 25 °C for 0.5 h, then at 80 °C for 2 h. The mixture was concentrated to get the residue. The residue was poured into water (30 mL). The aqueous phase was extracted with ethyl acetate (10 mL × 3). The combined organic phase was washed with brine (5 mL), dried with anhydrous Na_2_SO_4_, filtered, and concentrated *in vacuo*. The crude product was used to the next step without purification (90 mg 368 μmol, 21%). To a solution of the residue (90 mg, 368 μmol, 1 equiv) and [4-(2-aminoethyl)phenyl](azetidin-1-yl) (90 mg, 442 μmol, 1.2 equiv) in MeOH (1 mL) was added triethylamine (111 mg, 1.11 mmol, 153 μL, 3 equiv), and the reaction was stirred at 60 °C for 12 h. The mixture was cooled to rt, and the precipitate was collected, washed with MeOH (5 mL), and concentrated to afford *N*-(4-(azetidine-1-carbonyl)phenethyl)-3-(1*H*-benzo[*d*]imidazol-6-yl)-1,2,4-oxadiazole-5-carboxamide (90 mg, 216 μmol, 58%) was obtained as a purple solid. ^1^H NMR (400 MHz, DMSO): δ 12.87 (br s, 1H), 9.59 (br t, *J* = 5.1 Hz, 1H), 8.40 (s, 1H), 8.30 (s, 1H), 7.92 (dd, *J* = 1.2, 8.4 Hz, 1H), 7.79 (br d, *J* = 8.2 Hz, 1H), 7.57 (d, *J* = 8.2 Hz, 2H), 7.35 (d, *J* = 8.2 Hz, 2H), 4.29 (br t, *J* = 6.8 Hz, 2H), 4.03 (br t, *J* = 7.0 Hz, 2H), 3.58 (q, *J* = 6.8 Hz, 2H), 2.96 (br t, *J* = 7.3 Hz, 2H), 2.25 (q, *J* = 7.7 Hz, 2H). LC-MS: *m*/*z* 417 [M+H]^+^.

### *N*-(4-(Azetidine-1-carbonyl)phenethyl)-3-(benzofuran-6-yl)-1,2,4-oxadiazole-5-carboxamide (**64**)

To a mixture of *N*′-hydroxybenzofuran-6-carboximidamide (170 mg, 964 μmol, 1 equiv) and DIPEA (249 mg, 1.93 mmol, 336 μL, 2 equiv) in THF (3 mL) was dropwise added ethyl 2-chloro-2-oxo-acetate (158 mg, 1.16 mmol, 129 μL, 1.2 equiv) at 0 °C. The solution was stirred at 25 °C for 0.5 h and then at 80 °C for 2 h. The mixture was concentrated to get the residue, which was poured into water (30 mL). The aqueous phase was extracted with ethyl acetate (10 mL × 3). The combined organic phase was washed with brine (5 mL), dried with anhydrous Na_2_SO_4_, filtered, and concentrated *in vacuo*. The residue was purified by prep-TLC (SiO_2_, PE:EtOAc = 10:1). Ethyl 3-(benzofuran-6-yl)-1,2,4-oxadiazole-5-carboxylate (100 mg, crude, 40%) was obtained as yellow solid. To a solution of [4-(2-aminoethyl)phenyl]-(azetidin-1-yl)methanone (88 mg, 432 μmol, 16 μL, 1.12 equiv) and ethyl 3-(benzofuran-6-yl)-1,2,4-oxadiazole-5-carboxylate (100 mg, 387 μmol, 1 equiv) in MeOH (1 mL) was added triethylamine (109 mg, 1.08 mmol, 150 μL, 2.79 equiv), and the resulting mixture was stirred at 60 °C for 12 h. The mixture was concentrated to get the residue. The residue was purified by prep-HPLC (column: Waters Xbridge 150 × 25 5 μm; mobile phase: [water (0.05% ammonia hydroxide v/v)–ACN]; B%: 32–62%, 10 min). *N*-(4-(Azetidine-1-carbonyl)phenethyl)-3-(benzofuran-6-yl)-1,2,4-oxadiazole-5-carboxamide (17 mg, 40 μmol, 4%) was obtained as a white solid. ^1^H NMR (400 MHz, MeOD): δ 8.26 (s, 1H), 8.02 (dd, *J* = 1.3, 8.2 Hz, 1H), 7.93 (d, *J* = 2.1 Hz, 1H), 7.79 (d, *J* = 8.1 Hz,1H), 7.59 (d, *J* = 8.2 Hz, 2H), 7.40 (d, *J* = 8.2 Hz, 2H), 6.96 (dd, *J* = 0.9, 2.1 Hz, 1H), 4.37 (br t, *J* = 7.6 Hz, 2H), 4.19 (br t, *J* = 7.8 Hz, 2H), 3.70 (t, *J* = 7.3 Hz, 2H), 3.03 (t, *J* = 7.3 Hz, 2H), 2.36 (quin, *J* = 7.8 Hz, 2H). HRMS (ESI): calcd for [M+H]^+^ C_23_H_21_N_4_O_4_, 417.1563, found 417.1578.

### *N*-(4-(Azetidine-1-carbonyl)phenethyl)-3-(1*H*-indol-6-yl)-1,2,4-oxadiazole-5-carboxamide (**65**)

To a solution of (*Z*)-*N*′-hydroxy-1*H*-indole-6-carboximidamide (200 mg, 1.14 mmol, 1 equiv) and DIPEA (442 mg, 3.42 mmol, 596 μL, 3 equiv) in THF (3 mL) was added ethyl 2-chloro-2-oxo-acetate (155 mg, 1.14 mmol, 127 μL, 1 equiv) at 0 °C. The mixture was stirred at 25 °C for 0.5 h and then at 80 °C for 2 h, poured into water (5 mL), and extracted with EtOAc (10 mL × 3). The combined organic layer was washed with brine (15 mL), dried over Na_2_SO_4_, filtered, and concentrated. Ethyl 3-(1*H*-indol-6-yl)-1,2,4-oxadiazole-5-carboxylate (200 mg, crude, 68%) was obtained as a white solid. To a solution of ethyl 3-(1*H*-indol-6-yl)-1,2,4-oxadiazole-5-carboxylate (100 mg, 388 μmol, 1 equiv) and [4-(2-aminoethyl)phenyl]-(azetidin-1-yl)methanone (95 mg, 466 μmol, 1.2 equiv) in MeOH (3 mL) was added triethylamine (118 mg, 1.17 mmol, 162 μL, 3 equiv). The mixture was stirred at 60 °C for 16 h. The mixture was poured into water (5 mL) and extracted with EtOAc (10 mL × 3). The combined organic layer was washed with brine (15 mL), dried over Na_2_SO_4_, filtered, and concentrated. The residue was purified by prep-HPLC (column: Waters Xbridge 150 × 25 × 5 μm; mobile phase: [water (0.05% ammonia hydroxide v/v)–ACN]; B%: 25–55%, 10 min) and lyophilized to give *N*-(4-(azetidine-1-carbonyl)phenethyl)-3-(1*H*-indol-6-yl)-1,2,4-oxadiazole-5-carboxamide (22 mg, 52 μmol, 9%) as yellow solid. ^1^H NMR (400 MHz, DMSO-*d*_6_): δ = 11.52 (br s, 1H), 9.55 (br t, *J* = 5.9 Hz, 1H), 8.13 (s, 1H), 7.65–7.80 (m, 2H), 7.46–7.63 (m, 3H), 7.34 (d, *J* = 8.2 Hz, 2H), 6.55 (br s, 1H), 4.21–4.38(m, 2H), 4.02 (br s, 2H), 3.51–3.63 (m, 2H), 2.95 (br t, *J* = 7.2 Hz, 2H), 2.20–2.27 (m, 2H). HRMS (ESI): calcd for [M+H]^+^ C_23_H_22_N_5_O_3_, 416.1717, found 416.1728.

### *N*-(4-(Azetidine-1-carbonyl)phenethyl)-3-(1*H*-indazol-6-yl)-1,2,4-oxadiazole-5-carboxamide (**66**)

To a solution of (*Z*)-*N*′-hydroxy-1*H*-indazole-6-carboximidamide (200 mg, 1.14 mmol, 1 equiv) and DIPEA (440 mg, 3.41 mmol, 593 μL, 3 equiv) in THF (3 mL) was added ethyl 2-chloro-2-oxo-acetate (155 mg, 1.14 mmol, 127 μL, 1 equiv) at 0 °C. The mixture was stirred at 25 °C for 0.5 h and then at 80 °C for 2 h, poured into water (8 mL), and extracted with EtOAc (15 mL × 3). The combined organic layer was washed with brine (20 mL), dried over Na_2_SO_4_, filtered, and concentrated. Ethyl 3-(1*H*-indazol-6-yl)-1,2,4-oxadiazole-5-carboxylate (200 mg, crude, 67%) was obtained as a brown solid. To a solution of ethyl 3-(1*H*-indazol-6-yl)-1,2,4-oxadiazole-5-carboxylate (100 mg, 387 μmol, 1 equiv) and [4-(2-aminoethyl)phenyl]-(azetidin-1-yl)methanone (94 mg, 464 μmol, 1.2 equiv) in MeOH (3 mL) was added triethylamine (117 mg, 1.16 mmol, 161 μL, 3 equiv). The mixture was stirred at 60 °C for 16 h. The mixture was poured into water (5 mL) and extracted with EtOAc (10 mL × 3). The combined organic layer was washed with brine (10 mL), dried over Na_2_SO_4_, filtered, and concentrated. The residue was purified by prep-HPLC (column: Waters Xbridge 150 × 25 × 5 μm; mobile phase: [water (0.05% ammonia hydroxide v/v)–ACN];B%: 20–50%, 10 min) and lyophilized to give *N*-(4-(azetidine-1-carbonyl)phenethyl)-3-(1*H*-indazol-6-yl)-1,2,4-oxadiazole-5-carboxamide (13 mg, 30 μmol, 5%) as a white solid. ^1^H NMR (400 MHz, DMSO-*d*_6_): δ 13.48 (s, 1H), 9.47–9.72 (m, 1H), 8.23 (d, *J* = 11.4 Hz,2H), 7.98 (d, *J* = 8.3 Hz, 1H), 7.78 (d, *J* = 9.6 Hz, 1H), 7.56 (d, *J* = 8.2 Hz, 2H), 7.34 (d, *J* = 8.1 Hz, 2H), 4.28 (brs, 2H), 3.98–4.08 (m, 2H), 3.54–3.65 (m, 2H), 2.95 (br t, *J* = 7.1 Hz, 2H), 2.21–2.27 (m, 2H). HRMS (ESI): calcd for [M+H]^+^ C_22_H_21_N_6_O_3_, 417.1670, found 417.1670.

### *N*-(4-(Azetidine-1-carbonyl)phenethyl)-3-(3-methoxyphenyl)-1,2,4-oxadiazole-5-carboxamide (**67**)

To a solution of (*Z*)-*N*′-hydroxy-3-methoxybenzimidamide (180 mg, 1.08 mmol, 1 equiv) and DIPEA (419 mg, 3.25 mmol, 566 μL, 3 equiv) in THF (3 mL) was added ethyl 2-chloro-2-oxo-acetate (147 mg, 1.08 mmol, 121 μL, 1 equiv) at 0 °C. The mixture was stirred at 25 °C for 0.5 h and then at 80 °C for 2 h, poured into water (5 mL), and extracted with EtOAc (10 × 3). The combined organic layer was washed with brine (10 mL), dried over Na_2_SO_4_, filtered, and concentrated. Ethyl 3-(3-methoxyphenyl)-1,2,4-oxadiazole-5-carboxylate (150 mg, 604 μmol, crude, 55%) was obtained as a brown oil. To a solution of ethyl 3-(3-methoxyphenyl)-1,2,4-oxadiazole-5-carboxylate (150 mg, 604 μmol, 1 equiv) and [4-(2-aminoethyl)phenyl]-(azetidin-1-yl)methanone (148 mg, 725 μmol, 1.2 equiv) in MeOH (2 mL) was added triethylamine (183 mg, 1.81 mmol, 252 μL, 3 equiv). The mixture was stirred at 60 °C for 16 h. The mixture was poured into water (5 mL) and extracted with EtOAc (10 mL × 3). The combined organic layer was washed with brine (10 mL), dried over Na_2_SO_4_, filtered, and concentrated. The residue was purified by prep-HPLC (column: Waters Xbridge 150 × 25 × 5 μm; mobile phase: [water (0.05% ammonia hydroxide v/v)–ACN]; B%: 30–60%, 10 min) and lyophilized to give *N*-(4-(azetidine-1-carbonyl)phenethyl)-3-(3-methoxyphenyl)-1,2,4-oxadiazole-5-carboxamide (12 mg, 28 μmol, 2%) was obtained as a yellow solid.^1^H NMR (400 MHz, DMSO-*d*_6_): δ 9.45–9.70(m, 1H), 7.64 (d, *J* = 8.0 Hz, 1H), 7.47–7.60 (m, 4H), 7.33 (d, *J* = 8.2 Hz, 2H), 7.21 (dd, *J* = 8.2, 1.8 Hz, 1H), 4.28 (br s, 2H), 4.03 (br s, 2H), 3.85 (s, 3H), 3.52–3.64 (m, 2H), 2.94 (t, *J* = 7.4 Hz, 2H), 2.22–2.27 (m, 2H). HRMS (ESI): calcd for [M+H]^+^ C_22_H_23_N_4_O_4_, 407.1730, found 407.1719.

### (4-(2-Aminoethyl)phenyl)(azetidin-1-yl)methanone (**69**)

To a solution of 4-[2-(*tert*-butoxycarbonylamino)ethyl]benzoic acid (3.2 g, 12.06 mmol, 1 equiv) and azetidine (1.58 g, 16.89 mmol, 1.87 mL, HCl) in DCM (30 mL) were added DIPEA (4.68 g, 36.18 mmol, 6.30 mL) and HATU (5.50 g, 14.47 mmol). The mixture was stirred at 25 °C for 4 h, poured into water (20 mL), and extracted with EtOAc (30 mL × 3). The combined organic layer was washed with brine (40 mL), dried over Na_2_SO_4_, filtered, and concentrated. The residue was purified by column chromatography (SiO_2_, PE:EtOAc = 5:1 to 2:1). Compound *tert*-butyl *N*-[2-[4-(azetidine-1-carbonyl) phenyl]ethyl]carbamate (2.2 g, 7.23 mmol, 59%) was obtained as yellow oil. To a solution of *tert*-butyl *N*-[2-[4-(azetidine-1-carbonyl)phenyl]ethyl]carbamate (2.2 g, 7.23 mmol) in DCM (20 mL) was added TFA (7.70 g, 67.53 mmol, 5 mL). The mixture was stirred at 25 °C for 0.5 h, poured into NaHCO_3_ (70 mL), and extracted with EtOAc (100 mL × 3). The combined organic layer was washed with brine (10 mL), dried over Na_2_SO_4_, filtered, and concentrated. The crude product was purified by reversed-phase HPLC (0.1% NH_3_·H_2_O). (4-(2-Aminoethyl)phenyl)(azetidin-1-yl)methanone (1.2 g, 5.87 mmol, 81%) was obtained as a brown oil. LC-MS: *m*/*z* 205 [M+H]^+^.

### 3-(Cyclopropylmethoxy)-4-methoxybenzonitrile (**71**)

To a solution of 3-hydroxy-4-methoxy-benzonitrile (2.00 g, 13.4 mmol, 1.00 equiv) and bromomethylcyclopropane (2.72 g, 20.1 mmol, 1.93 mL, 1.50 equiv) in DMF (20 mL) was added K_2_CO_3_ (3.71 g, 26.8 mmol, 2.00 equiv). The mixture was stirred at 40 °C for 12 h, poured into water (30 mL), and extracted with EtOAc (20 mL × 3). The combined organic layer was washed with brine (40 mL), dried over Na_2_SO_4_, filtered, and concentrated. The crude product was used to the next step directly. 3-(cyclopropylmethoxy)-4-methoxy-benzonitrile (2.30 g, 11.3 mmol, 84%, crude) was obtained as yellow solid. ^1^H NMR (400 MHz, DMSO-*d*_6_): δ 7.40 (dd, *J* = 2.0, 8.4 Hz, 1H), 7.32 (d, *J* = 2.0 Hz, 1H), 7.11 (d, *J* = 8.4 Hz, 1H), 3.89–3.80 (m, 5H), 1.30–1.12 (m, 1H), 0.67–0.50 (m, 2H), 0.37–0.21 (m, 2H). LC-MS: *m*/*z* 204 [M+H]^+^.

### (*Z*)-3-(Cyclopropylmethoxy)-*N*′-hydroxy-4-methoxybenzimidamide (**74**)

To a solution of 3-(cyclopropylmethoxy)-4-methoxybenzonitrile (1.00 g, 4.92 mmol, 1.00 equiv) and hydroxylamine (513 mg, 7.38 mmol, 1.50 equiv, HCl) in EtOH (15 mL) was added DIPEA (1.27 g, 9.84 mmol, 1.71 mL, 2.00 equiv). The mixture was stirred at 80 °C for 12 h, poured into H_2_O (20 mL), and extracted with EtOAc (20 mL × 3). The combined organic layer was washed with brine (20 mL), dried over Na_2_SO_4_, filtered, and concentrated. (*Z*)-3-(Cyclopropylmethoxy)-*N*′-hydroxy-4-methoxybenzimidamide (800 mg, 3.39 mmol, 69%) was obtained as a white solid. The crude product was used to the next step directly. LC-MS: *m*/*z* 237 [M+H]^+^.

### 3-[3-(Cyclopropylmethoxy)-4-methoxyphenyl]-*N*-[2- [4-(3-methoxyazetidine-1-carbonyl) phenyl]ethyl]-1,2,4-oxadiazole-5-carboxamide (**77**)

To a solution of (*Z*)-3-(cyclopropylmethoxy)-*N*′-hydroxy-4-methoxybenzimidamide (500 mg, 2.12 mmol, 1.00 equiv) and DIPEA (821 mg, 6.35 mmol, 1.11 mL, 3.00 equiv) in THF (6 mL) was added ethyl 2-chloro-2-oxo-acetate (289 mg, 2.12 mmol, 237 μL, 1.00 equiv) at 0 °C. The mixture was stirred at 20 °C for 0.5 h and then at 60 °C for 2 h, poured into water (10 mL), and extracted with EtOAc (10 mL × 3). The combined organic layer was washed with brine (20 mL), dried over Na_2_SO_4_, filtered, and concentrated. Ethyl 3-[3-(cyclopropylmethoxy)-4-methoxy-phenyl]-1,2,4-oxadiazole-5-carboxylate (500 mg, crude, 77%) was obtained as colorless oil. To a solution of ethyl 3-[3-(cyclopropylmethoxy)-4-methoxy-phenyl]-1,2,4-oxadiazole-5-carboxylate (400 mg, 1.26 mmol, 1.00 equiv) and [4-(2-aminoethyl)phenyl]-(3- methoxyazetidin-1-yl)methanone (340 mg, 1.26 mmol, 1.00 equiv, HCl) in MeOH (4 mL) was added triethylamine (381 mg, 3.77 mmol, 525 μL, 3.00 equiv). The mixture was stirred at 60 °C for 2 h, poured into H_2_O (10 mL), and extracted with EtOAc (30 mL × 3). The combined organic layer was washed with brine (20 mL), dried over Na_2_SO_4_, filtered, and concentrated. The residue was purified by prep-HPLC (column: Waters Xbridge C18 150 × 50 mm × 10 μm; mobile phase: [water (10 mM NH_4_HCO_3_)–ACN]; B%: 30–60%, 11.5 min) and lyophilized. 3-[3-(cyclopropylmethoxy)-4-methoxy-phenyl]-*N*-[2-[4-(3-methoxyazetidine-1-carbonyl)phenyl]ethyl]-1,2,4-oxadiazole-5-carboxamide (78 mg, 154 μmol, 9%) was obtained as a white solid. ^1^H NMR (400 MHz, CDCl_3_): δ 7.67 (dd, *J* = 2.0, 8.4 Hz, 1H), 7.64–7.58 (m, 2H), 7.56 (d, *J* = 2.0 Hz, 1H), 7.31 (d, *J* = 8.0 Hz, 2H), 7.18 (t, *J* = 6.0 Hz, 1H), 6.97 (d, *J* = 8.4 Hz, 1H), 4.39 (s, 2H), 4.25 (tt, *J* = 4.0, 6.4 Hz, 1H), 4.21–4.02 (m, 2H), 3.95 (s, 3H), 3.95 (s, 1H), 3.93 (s, 1H), 3.78 (q, *J* = 6.8 Hz, 2H), 3.32 (s, 3H), 3.03 (t, *J* = 7.2 Hz, 2H), 1.45–1.31 (m, 1H), 0.75–0.61 (m, 2H), 0.46–0.32 (m, 2H). HRMS (ESI): calcd for [M+H]^+^ C_27_H_31_N_4_O_6_, 507.2238, found 507.2238.

### 3-Cyclobutoxy-4-methoxybenzonitrile (**72**)

To a solution of 3-cyclobutoxy-4-methoxybenzonitrile (2.00 g, 13.4 mmol, 1.00 equiv) and bromocyclobutane (2.35 g, 17.4 mmol, 1.65 mL, 1.30 equiv) in DMF (16 mL) was added K_2_CO_3_ (3.71 g, 26.8 mmol, 2.00 equiv) at 20 °C. The mixture was stirred at 40 °C for 16 h and then at 60 °C for 3 h, poured into water (100 mL), and then extracted with EtOAc (50 mL × 3). The combined organic layers were dried over Na_2_SO_4_, filtered, and concentrated *in vacuo* to give a residue. The residue was purified by silica gel chromatography (PE:EtOAc = 100:1–10:1). 3-Cyclobutoxy-4-methoxybenzonitrile (1.80 g, 8.86 mmol, 66%) was obtained as a white solid. ^1^H NMR (400 MHz, CDCl_3_): δ 7.27 (s, 1H), 6.95–6.86 (m, 2H), 4.71–4.58 (m, 1H), 3.92 (s, 3H), 2.56–2.42 (m, 2H), 2.34–2.18 (m, 2H), 1.96–1.83 (m, 1H), 1.80–1.66 (m, 1H). LC-MS: *m*/*z* 204 [M+H]^+^.

### (*Z*)-3-Cyclobutoxy-*N′*-hydroxy-4-methoxybenzimidamide (**75**)

To a solution of 3-(cyclobutoxy)-4-methoxybenzonitrile (1.80 g, 8.86 mmol, 1.00 equiv) and hydroxylamine (923 mg, 13.3 mmol, 1.50 equiv, HCl) in EtOH (20 mL) was added DIPEA (3.43 g, 26.6 mmol, 4.63 mL, 3.00 equiv). The mixture was stirred at 80 °C for 12 h, poured into H_2_O (10 mL), and extracted with EtOAc (30 mL × 3). The combined organic layer was washed with brine (20 mL), dried over Na_2_SO_4_, filtered, and concentrated. The crude product was washed with EtOH (5 mL) and filtered. (*Z*)-3-Cyclobutoxy-*N*′-hydroxy-4-methoxybenzimidamide (1.00 g, 4.23 mmol, 48%) was obtained as a white solid. ^1^H NMR (400 MHz, DMSO-*d*_6_): δ 9.46 (s, 1H), 7.20 (dd, *J* = 2.0, 8.4 Hz, 1H), 7.10 (d, *J* = 2.0 Hz, 1H), 6.93 (d, *J* = 8.4 Hz, 1H), 5.70 (s, 2H), 4.70–4.55 (m, 1H), 3.76 (s, 3H), 2.44–2.30 (m, 2H), 2.15–1.94 (m, 2H), 1.84–1.55 (m, 2H). LC-MS: *m*/*z* 237 [M+H]^+^.

### 3-[3-(Cyclobutoxy)-4-methoxy-phenyl]-*N*-[2-[4-(3-methoxyazetidine-1-carbonyl)phenyl]ethyl]-1,2,4-oxadiazole-5-carboxamide (**78**)

To a solution of (*Z*)-3-cyclobutoxy-*N*′-hydroxy-4-methoxybenzimidamide (500 mg, 2.12 mmol, 1.00 equiv) and DIPEA (547 mg, 4.23 mmol, 737 μL, 2.00 equiv) in THF (6 mL) was added ethyl 2-chloro-2-oxo-acetate (289 mg, 2.12 mmol, 237 μL, 1.00 equiv) at 0 °C. The mixture was stirred at 20 °C for 0.5 h and then at 60 °C for 2 h, poured into H_2_O (10 mL), and extracted with EtOAc (10 mL × 3). The combined organic layer was washed with brine (20 mL), dried over Na_2_SO_4_, filtered, and concentrated. Ethyl 3-(3-cyclobutoxy-4-methoxyphenyl)-1,2,4-oxadiazole-5-carboxylate (400 mg, crude, 59%) was obtained as yellow oil. To a solution of ethyl 3-[3-(cyclobutoxy)-4-methoxyphenyl]-1,2,4-oxadiazole-5-carboxylate (400 mg, 1.26 mmol, 1.00 equiv) and [4-(2-aminoethyl)phenyl]-(3-methoxyazetidin-1-yl)methanone (374 mg, 1.38 mmol, 1.10 equiv, HCl) in MeOH (4 mL) was added triethylamine (381 mg, 3.77 mmol, 525 μL, 3.00 equiv). The mixture was stirred at 60 °C for 2 h, poured into H_2_O (6 mL), and extracted with EtOAc (10 mL × 3). The combined organic layer was washed with brine (20 mL), dried over Na_2_SO_4_, filtered, and concentrated. The residue was purified by prep-HPLC (column: Waters Xbridge C18 150 × 50 mm × 10 μm; mobile phase: [water (10 mMNH_4_HCO_3_)–ACN]; B%: 30–60%, 11.5 min) and lyophilized. 3-[3-(cyclobutoxy)-4-methoxy-phenyl]-*N*-[2-[4-(3-methoxyazetidine-1-carbonyl)phenyl]ethyl]-1,2,4-oxadiazole-5-carboxamide (115 mg, 226 μmol, 18%) was obtained as a white solid. ^1^H NMR (400 MHz, CDCl_3_): δ 7.72–7.57 (m, 3H), 7.42 (d, *J* = 1.6 Hz, 1H), 7.31 (d, *J* = 8.0 Hz, 2H), 7.17 (t, *J* = 6.0 Hz, 1H), 6.96 (d, *J* = 8.4 Hz, 1H), 4.77 (t, *J* = 7.2 Hz, 1H), 4.39 (d, *J* = 5.6 Hz, 2H), 4.30–4.22 (m, 1H), 4.21–4.03 (m, 2H), 3.95 (s, 3H), 3.79 (q, *J* = 6.8 Hz, 2H), 3.32 (s, 3H), 3.03 (t, *J* = 7.2 Hz, 2H), 2.61–2.47 (m, 2H), 2.40–2.19 (m, 2H), 1.90–1.70 (m, 1H), 1.81–1.66 (m, 1H). HRMS (ESI): calcd for [M+H]^+^ C_27_H_31_N_4_O_6_, 507.2238, found 507.2246.

### 3-Cyclopropoxy-4-methoxybenzonitrile (**73**)

To a solution of 3-hydroxy-4-methoxybenzonitrile (2.00 g, 13.4 mmol, 1.00 equiv) and bromocyclopropane (4.87 g, 40.2 mmol, 3.22 mL, 3.00 equiv) in dry DMF (30 mL) were added Cs_2_CO_3_ (17.5 g, 53.6 mmol, 4.00 equiv) and KI (333 mg, 2.01 mmol, 0.15 equiv). The mixture was stirred at 140 °C for 12 h, poured into H_2_O (30 mL), and extracted with EtOAc (20 mL × 3). The combined organic layer was washed with brine (40 mL), dried over Na_2_SO_4_, filtered, and concentrated. The residue was purified by column chromatography (SiO_2_, PE:EtOAc = 20:1 to 10:1 to 5:1). 3-Cyclopropoxy-4-methoxybenzonitrile (600 mg, 3.17 mmol, 23%) was obtained as a white solid. ^1^H NMR (400 MHz, CDCl_3_): δ 7.48 (d, *J* = 2.0 Hz, 1H), 7.30 (dd, *J* = 2.0, 8.4 Hz, 1H), 6.89 (d, *J* = 8.4 Hz, 1H), 3.91 (s, 3H), 3.80–3.71 (m, 1H), 0.92–0.81 (m, 4H). LC-MS: *m*/*z* 190 [M+H]^+^.

### (*Z*)-3-Cyclopropoxy-*N*′-hydroxy-4- methoxybenzimidamide (76)

To a solution of 3-cyclopropoxy-4-methoxybenzonitrile (500 mg, 2.64 mmol, 1.00 equiv) and hydroxylamine (220 mg, 3.17 mmol, 1.20 equiv, HCl) in EtOH (6 mL) was added DIPEA (1.02 g, 7.93 mmol, 1.38 mL, 3.00 equiv). The mixture was stirred at 70 °C for 12 h, poured into H_2_O (10 mL), and extracted with EtOAc (10 mL × 3). The combined organic layer was washed with brine (20 mL), dried over Na_2_SO_4_, filtered, and concentrated. (*Z*)-3-cyclopropoxy-*N*′-hydroxy-4- methoxybenzimidamide (600 mg, crude, >100%) was obtained as a white solid. LC-MS: *m*/*z* 223 [M+H]^+^.

### 3-(3-Cyclopropoxy-4-methoxyphenyl)-*N*-(4-(3-methoxyazetidine-1-carbonyl)phenethyl)-1,2,4-oxadiazole-5-carboxamide (**79**)

To a solution of (*Z*)-3-cyclopropoxy-*N*′-hydroxy-4-methoxybenzimidamide (600 mg, 2.70 mmol, 1.00 equiv) and DIPEA (1.05 g, 8.10 mmol, 1.41 mL, 3.00 equiv) in THF (8 mL) was added ethyl 2-chloro-2-oxo-acetate (369 mg, 2.70 mmol, 302 μL, 1.00 equiv) at 0 °C. The mixture was stirred at 60 °C for 2 h, poured into H_2_O (10 mL), and extracted with EtOAc (10 mL × 3). The combined organic layer was washed with brine (20 mL), dried over Na_2_SO_4_, filtered, and concentrated. The residue was purified by column chromatography (SiO_2_, PE:EtOAc = 5:1 to 4:1) to afford ethyl 3-(3-cyclopropoxy-4-methoxyphenyl)-1,2,4-oxadiazole-5-carboxylate (500 mg, 1.56 mmol, 57%). To a solution of ethyl 3-(3-cyclopropoxy-4-methoxyphenyl)-1,2,4-oxadiazole-5-carboxylate (200 mg, 657 μmol, 1.00 equiv) and [4-(2-aminoethyl)phenyl]-(3-methoxyazetidin-1-yl)methanone (214 mg, 789 μmol, 1.20 equiv, HCl) in MeOH (4 mL) was added triethylamine (200 mg, 1.97 mmol, 274 μL, 3.00 equiv). The mixture was stirred at 70 °C for 2 h, poured into H_2_O (10 mL), and extracted with EtOAc (10 mL × 3). The combined organic layer was washed with brine (20 mL), dried over Na_2_SO_4_, filtered, and concentrated. The residue was purified by prep-HPLC (column: Phenomenex Synergi C18 150 × 25 × 10 μm; mobile phase: [water (0.225%FA)–ACN]; B%: 45–75%, 9 min) and lyophilized. 3-(3-Cyclopropoxy-4-methoxyphenyl)-*N*-(4-(3-methoxyazetidine-1-carbonyl) phenethyl)-1,2,4-oxadiazole-5-carboxamide (15 mg, 32 μmol, 4%) was obtained as a white solid. ^1^H NMR (400 MHz, DMSO): δ 9.53 (t, *J* = 6.0 Hz, 1H), 7.84 (d, *J* = 2.0 Hz, 1H), 7.67 (dd, *J* = 2.0, 8.4 Hz, 1H), 7.58 (d, *J* = 8.4 Hz, 2H), 7.34 (d, *J* = 8.4 Hz, 2H), 7.17 (d, *J* = 8.4 Hz, 1H), 4.41 (d, *J* = 5.2 Hz, 1H), 4.29–4.17 (m, 2H), 4.16–4.05 (m,1H), 3.93 (td, *J* = 2.8, 5.6 Hz, 1H), 3.83 (s, 4H), 3.63–3.51 (m, 2H), 3.21 (s, 3H), 2.94 (t, *J* = 7.2 Hz, 2H), 0.88–0.79 (m, 2H), 0.76–0.66 (m, 2H). LC-MS: *m*/*z* 493 [M+H]^+^.

### 3-(3-((1-Hydroxycyclopropyl)methoxy)-4-methoxyphenyl)-*N*-(4-(3-methoxyazetidine-1-carbonyl)phenethyl)-1,2,4-oxadiazole-5-carboxamide (**80**)

To a solution of ethyl 3-(3-hydroxy-4-methoxy-phenyl)-1,2,4-oxadiazole-5-carboxylate (1.2 g, 4.54 mmol, 1 equiv) and (1-tetrahydropyran-2-yloxycyclopropyl)methanol (860 mg, 5.00 mmol, 1.1 equiv) in THF (12 mL) were added PPh_3_ (1.43 g, 5.45 mmol, 1.2 equiv) and DEAD (949 mg, 5.45 mmol, 990 μL, 1.2 equiv) at 0 °C under N_2_. The mixture was stirred at 25 °C for 2 h and then concentrated. The crude product was purified by prep-HPLC (column: Phenomenex Luna C18 150 × 40 mm × 15 μm; mobile phase: [water (0.225%FA)–ACN]; B%: 38–68%, 9 min). The eluent was concentrated and lyophilized. Impure ethyl 3-[3-[(1-hydroxycyclopropyl)methoxy]-4-methoxy-phenyl]-1,2,4-oxadiazole-5-carboxylate was obtained (950 mg, 1.28 mmol, 28% yield, 45.2% purity).To a solution of (4-(2-aminoethyl)phenyl)(3-methoxyazetidin-1-yl)methanone (923 mg, 3.41 mmol, 1.20 equiv, HCl) and triethylamine (863 mg, 8.52 mmol, 1.19 mL, 3.00 equiv) in MeOH (10.0 mL) was added ethyl 3-(3-((1-hydroxycyclopropyl)methoxy)-4-methoxyphenyl)-1,2,4-oxadiazole-5-carboxylate (950 mg, 2.84 mmol, 1.00 equiv), and the reaction was stirred at 60 °C for 2 h. The mixture was concentrated to 5.00 mL and purified by prep-HPLC (column: Waters Xbridge C18 150 × 50 mm × 10 μm; mobile phase: [water (10 mM NH_4_HCO_3_)–ACN]; B%: 25–55%, 11.5 min), and the eluent was concentrated and lyophilized. 3-(3-((1-Hydroxycyclopropyl)methoxy)-4-methoxyphenyl)-*N*-(4-(3-methoxyazetidine-1-carbonyl)phenethyl)-1,2,4-oxadiazole-5-carboxamide (52 mg, 96 μmol, 3% yield) was obtained as a white solid. ^1^H NMR (500 MHz, DMSO): δ 9.52 (t, *J* = 5.6 Hz, 1H), 7.68–7.65 (m, 1H), 7.60–7.57 (m, 3H), 7.36–7.33 (m, 2H), 7.20–7.18 (m, 1H), 5.55 (s, 1H), 4.46–4.49 (m, 1H), 4.26–4.21 (m, 2H), 4.12–4.10 (m, 1H), 4.05–4.04 (m, 2H), 3.90–3.53 (m, 4H), 3.61–3.53 (m, 2H), 3.23–3.22 (m, 3H), 2.95 (t, *J* = 7.3 Hz, 2H), 0.73–0.64 (m, 4H). LC-MS: *m*/*z* 454 [M+H]^+^.

### Ethyl 3-(3-Hydroxy-4-methoxyphenyl)-1,2,4-oxadiazole-5-carboxylate (**82**)

To a solution of (*Z*)-*N*′,3-dihydroxy-4-methoxybenzimidamide (2.00 g, 11.0 mmol, 1.00 equiv) and DIPEA (2.13 g, 16.5 mmol, 2.87 mL, 1.50 equiv) in THF (30.0 mL) was added ethyl 2-chloro-2-oxoacetate (1.50 g, 11.0 mmol, 1.23 mL, 1.00 equiv) at 0 °C. The mixture was stirred at 80 °C for 2 h and then concentrated *in vacuo*. The crude product was purified by column chromatography (SiO_2_, PE:EtOAc = 10:1 to 2:1), and the eluant was concentrated *in vacuo*. Impure ethyl 3-(3-hydroxy-4-methoxyphenyl)-1,2,4-oxadiazole-5-carboxylate (2.38 g, 6.81 mmol, 62% yield, 76% purity) was obtained as a yellow solid. ^1^H NMR (400 MHz, CDCl_3_): δ 7.75–7.64 (m, 2H), 6.99–6.91 (m, 1H), 5.77 (br s, 1H), 4.57 (q, *J* = 7.2 Hz, 2H), 3.97 (s, 3H), 1.49 (t, *J* = 7.2 Hz, 3H). LC-MS: *m*/*z* 265 [M+H]^+^.

### (4-(2-Aminoethyl)phenyl)(3-methoxyazetidin-1-yl)methanone Hydrochloride (**83**)

To a solution of 4-[2-(*tert*-butoxycarbonylamino)ethyl]benzoic acid (1.75 g, 6.60 mmol, 1 equiv), 3-methoxyazetidine (896 mg, 7.26 mmol, 1.1 equiv, HCl), and HATU (3.76 g, 9.89 mmol, 1.5 equiv) in DCM (30 mL) was added DIPEA (2.56 g, 19.79 mmol, 3.45 mL, 3 equiv). The mixture was stirred at 25 °C for 16 h and then concentrated *in vacuo* to remove DCM. The residue was diluted with H_2_O (50 mL) and extracted with EtOAc (50 mL × 3). The organic layers were combined, washed with brine (50 mL), dried over Na_2_SO_4_, and concentrated *in vacuo*. The crude product was purified by column chromatography (SiO_2_, PE:EtOAc = 3:1 to 1:2), the eluent was concentrated *in vacuo*. *tert*-Butyl *N*-[2-[4-(3-methoxyazetidine-1-carbonyl)phenyl]ethyl]carbamate (2.2 g, 6.01 mmol, 91%) was obtained as a colorless oil. ^1^H NMR (400 MHz, DMSO-*d*_6_): δ 7.55 (d, *J* = 8.0 Hz, 2H), 7.26 (d, *J* = 8.0 Hz, 2H), 6.91 (br t, *J* = 5.6 Hz, 1H), 4.41 (br d, *J* = 4.8 Hz, 1H), 4.27–4.16 (m, 2H), 4.11 (br d, *J* = 8.4 Hz, 1H), 3.82 (br d, *J* = 6.0 Hz, 1H), 3.21 (s, 3H), 3.19–3.10 (m, 2H), 2.73 (t, *J* = 7.2 Hz, 2H), 1.35 (s, 9H). To a solution of *tert*-butyl *N*-[2-[4-(3-methoxyazetidine-1-carbonyl)phenyl]ethyl]carbamate (2.2 g, 6.58 mmol, 1 equiv) in dioxane (5 mL) was added HCl/dioxane (4 M, 20 mL, 12.16 equiv) at 0 °C. The mixture was stirred at 25 °C for 1 h and then concentrated *in vacuo*. (4-(2-Aminoethyl)phenyl)(3-methoxyazetidin-1-yl)methanone (2.2 g, crude, HCl, >100%) was obtained as a yellow oil. LC-MS: *m*/*z* 235 [M+H]^+^.

### 2-[2-(3,4-Dimethoxyphenyl)-1,3-dioxolan-2-yl]acetonitrile (**85**)

To a solution of 3-(3,4-dimethoxyphenyl)-3-oxo-propanenitrile (500 mg, 2.44 mmol) in toluene (10 mL) were added ethylene glycol (0.3 mL, 4.87 mmol) and 4-methylbenzenesulfonic acid hydrate (23 mg, 0.12 mmol). The reaction mixture was heated at 110 °C for 16 h, concentrated *in vacuo*, dissolved in DCM (10 mL), washed with sat. NaHCO_3_ (3 × 10 mL), passed through a hydrophobic frit, and concentrated *in vacuo*. Purification by flash column chromatography afforded 2-[2-(3,4-dimethoxyphenyl)-1,3-dioxolan-2-yl]acetonitrile (294 mg, 48%) as a yellow solid. ^1^H NMR (400 MHz, DMSO-*d*_6_): δ 7.00 (dd, *J* = 6.6, 2.1 Hz, 2H), 6.94 (dd, *J* = 6.2, 2.8 Hz, 1H), 4.10 (td, *J* = 4.9, 3.3 Hz, 2H), 3.83 (td, *J* = 4.9, 3.2 Hz, 2H), 3.76 (s, 3H), 3.75 (s, 3H), 3.27 (s, 2H).

### 5-(3,4-Dimethoxyphenyl)isoxazol-3-amine (**86**)

To a solution of hydroxylamine hydrochloride (323 mg, 4.65 mmol) in methanol (1 mL) was added 7 M NH_3_/methanol (0.8 mL, 5.82 mmol), and the reaction mixture was stirred at rt for 30 min. Quinolin-8-ol (17 mg, 0.12 mmol) was added, followed by a solution of (3,4-dimethoxyphenyl)-1,3-dioxolan-2-yl]acetonitrile (290 mg, 1.16 mmol) in methanol (1 mL). The reaction mixture was heated at 70 °C for 16 h, concentrated *in vacuo*, azeotroped with toluene (3 × 10 mL), dissolved in ethanol (5 mL), acidified to pH 1 with conc. HCl, and heated at 120 °C overnight. The reaction mixture was cooled and concentrated *in vacuo* then dissolved in DCM (10 mL) and washed with sat. NaHCO_3_ (10 mL). The aqueous layer was extracted with DCM (2 × 10 mL), and the combined organics were passed through a hydrophobic frit and concentrated *in vacuo*. Purification by flash column chromatography afforded 5-(3,4-dimethoxyphenyl)isoxazol-3-amine (79 mg, 31%) as a yellow solid. ^1^H NMR (500 MHz, DMSO-*d*_6_): δ 7.31 (dd, *J* = 8.3, 2.0 Hz, 1H), 7.3 (d, *J* = 2.0 Hz, 1H), 7.04 (d, *J* = 8.4 Hz, 1H), 6.21 (s, 1H), 5.57 (s, 2H), 3.82 (s, 3H), 3.80 (s, 3H). LC-MS: *m*/*z* 221 [M+H]^+^.

### 3-(3,4-Dimethoxyphenyl)-*N*-[5-(3,4-dimethoxyphenyl)isoxazol-3-yl]propenamide (**87**)

To a solution of 3-(3,4-dimethoxyphenyl)propanoic acid (34 mg, 0.16 mmol) in DCM (5 mL) was added thionyl chloride (59 μL, 0.82 mmol), and the reaction mixture was heated at 40 °C overnight. The reaction mixture was cooled, concentrated *in vacuo*, and azeotroped with DCM (3 × 10 mL) then dissolved in DMF (5 mL). 5-(3,4-Dimethoxyphenyl)isoxazol-3-amine (30 mg, 0.14 mmol) was added followed by triethylamine (38 μL, 0.28 mmol), and the reaction mixture was stirred at 50 °C for 16 h. The reaction mixture was concentrated *in vacuo* then diluted with EtOAc (10 mL), washed with water (3 × 10 mL), passed through a hydrophobic frit, and concentrated *in vacuo*. Purification by prep-HPLC afforded 3-(3,4-dimethoxyphenyl)-*N*-[5-(3,4-dimethoxyphenyl)isoxazol-3-yl]propenamide (12 mg, 19%) as a white solid. ^1^H NMR (500 MHz, DMSO-*d*_6_): δ 10.96 (s, 1H), 7.43 (dd, *J* = 8.3, 2.0 Hz, 1H), 7.39 (d, *J* = 2.0 Hz, 1H), 7.28 (s, 1H), 7.08 (d, *J* = 8.5 Hz, 1H), 6.86–6.84 (m, 2H), 6.74 (dd, *J* = 8.1, 1.9 Hz, 1H), 3.85 (s, 3H), 3.82 (s, 3H), 3.73 (s, 3H), 3.71 (s, 3H), 2.85 (t, *J* = 7.6 Hz, 2H), 2.69 (t, *J* = 7.6 Hz, 2H). LC-MS: *m*/*z* 413 [M+H]^+^.

### Ethyl 5-(3,4-Dimethoxyphenyl)-1,2,4-oxadiazole-3-carboxylate (**89**)

A solution of ethyl 2-(hydroxyamino)-2-iminoacetate (400 mg, 3.02 mmol) in DCM (5 mL) was cooled to 0 °C, and triethylamine (0.63 mL, 4.54 mmol) was added, followed by 3,4-dimethoxybenzoyl chloride (607 mg, 3.02 mmol) portionwise. The reaction mixture was stirred at room temperature overnight. The reaction mixture was evaporated *in vacuo*, DMF (3 mL) was added, and the reaction mixture heated at 150 °C overnight. The cooled reaction mixture was partitioned between water (20 mL) and EtOAc (20 mL). The EtOAc extract was washed with water (2 × 20 mL) and evaporated *in vacuo*. The residue was purified by silica (12 g) eluting with 0–100% EtOAc/heptane to afford ethyl 5-(3,4-dimethoxyphenyl)-1,2,4-oxadiazole-3-carboxylate (27 mg, 2.8%). ^1^H NMR (400 MHz, CDCl_3_): δ 7.88 (dd, *J* = 2.0, 8.4 Hz, 1H), 7.70 (d, *J* = 1.9 Hz, 1H), 7.04–7.01 (m, 1H), 4.57 (q, *J* = 7.2 Hz, 2H), 4.01 (d, *J* = 2.6 Hz, 6H), 1.50 (t, *J* = 2.7 Hz, 3H). LC-MS: *m*/*z* 279 [M+H]^+^.

### 5-(3,4-Dimethoxyphenyl)-*N*-phenethyl-1,2,4-oxadiazole-3-carboxamide (**90**)

To a solution of ethyl 5-(3,4-dimethoxyphenyl)-1,2,4-oxadiazole-3-carboxylate (55 mg, 0.19 mmol) and phenethylamine (21 mg, 0.17 mmol) in MeOH (5 mL) was added Et_3_N (0.068 mL, 0.49 mmol). The reaction mixture was stirred at 60 °C overnight, concentrated *in vacuo*, dissolved in EtOAc (10 mL), and washed with water (10 mL), and the aqueous phase was extracted further with EtOAc (2 × 10 mL). The EtOAc extracts were evaporated *in vacuo*. The residue was purified by mass-directed HPLC 5–95% MeCN basic to afford 5-(3,4-dimethoxyphenyl)-*N*-phenethyl-1,2,4-oxadiazole-3-carboxamide (30 mg, 40%). ^1^H NMR (500 MHz, DMSO): δ 9.09–9.04 (m, 1H), 7.78 (dd, *J* = 2.0, 8.4 Hz, 1H), 7.62 (d, *J* = 2.1 Hz, 1H), 7.34–7.22 (m, 6H), 3.90–3.89 (m, 6H), 3.56–3.50 (m, 2H), 2.91–2.87 (m, 2H). HRMS (ESI): calcd for [M+H]^+^ C_19_H_20_N_3_O_4_, 354.1454, found 354.1444.

### Methyl 4-(3,4-Dimethoxyphenyl)furan-2-carboxylate (**92**)

To a mixture of methyl 4-bromofuran-2-carboxylate (150 mg, 0.73 mmol) and (3,4-dimethoxyphenyl)boronic acid (133 mg, 0.73 mmol) in 1,4-dioxane (4 mL) and water (1 mL) was added K_3_PO_4_ (310 mg, 1.46 mmol), and the reaction mixture was degassed with nitrogen for 10 min. Pd(dtbpf)Cl_2_ (48 mg, 0.07 mmol) was added, and the reaction mixture was degassed with nitrogen for 10 min then heated at 80 °C for 16 h. The reaction mixture was cooled, filtered through Celite, and washed with EtOAc (3 × 5 mL). The combined organics were washed with water (3 × 10 mL), passed through a hydrophobic frit, and concentrated *in vacuo* to afford methyl 4-(3,4-dimethoxyphenyl)furan-2-carboxylate as an orange solid. ^1^H NMR (500 MHz, DMSO-*d*_6_): δ 8.41 (d, *J* = 0.8 Hz, 1H), 7.84 (d, *J* = 0.9 Hz, 1H), 7.28 (d, *J* = 2.0 Hz, 1H), 7.23 (dd, *J* = 8.3, 2.1 Hz, 1H), 6.97 (d, *J* = 8.3 Hz, 1H), 3.84 (s, 3H), 3.82 (s, 3H), 3.77 (s, 3H). LC-MS: *m*/*z* 263 [M+H]^+^.

### 4-(3,4-Dimethoxyphenyl)furan-2-carboxylic Acid (**93**)

To a solution of methyl 4-(3,4-dimethoxyphenyl)furan-2-carboxylate (168 mg, 0.64 mmol) in water (5 mL) and ethanol (5 mL) was added sodium hydroxide (109 mg, 2.72 mmol). The reaction mixture was heated at 80 °C for 1 h, cooled, neutralized with 2 M HCl, extracted with EtOAc (3 × 10 mL), passed through a hydrophobic frit, and concentrated *in vacuo* to afford 4-(3,4-dimethoxyphenyl)furan-2-carboxylic acid as a brown solid. ^1^H NMR (500 MHz, DMSO-*d*_6_): δ 13.07 (br s, 1H), 8.34 (s, 1H), 7.71 (s, 1H), 7.26 (s, 1H), 7.21 (d, *J* = 8.4 Hz, 1H), 6.97 (d, *J* = 8.3 Hz, 1H), 3.82 (s, 3H), 3.77 (s, 3H). LC-MS: *m*/*z* 248 [M+H]^+^.

### 4-(3,4-Dimethoxyphenyl)-*N*-(2-phenylethyl)furan-2-carboxamide (**94**)

To a solution of 4-(3,4-dimethoxyphenyl)furan-2-carboxylic (165 mg, 0.67 mmol), HATU (278 mg, 0.73 mmol), and triethylamine (232 μL, 1.66 mmol) in DMF (5 mL) was added 2-phenylethanamine (72 mg, 0.6 mmol). The reaction mixture was stirred at rt overnight for 16 h, concentrated *in vacuo*, diluted with EtOAc (20 mL), washed with water (5 × 20 mL), passed through a hydrophobic frit, and concentrated *in vacuo*. Purification by prep-HPLC afforded 4-(3,4-dimethoxyphenyl)-*N*-(2-phenylethyl)furan-2-carboxamide (32 mg, 12%) as an off-white solid. ^1^H NMR (500 MHz, DMSO-*d*_6_): δ 8.44 (t, *J* = 5.7 Hz, 1H), 8.25 (d, *J* = 0.8 Hz, 1H), 7.52 (d, *J* = 0.8 Hz, 1H), 7.31–7.28 (m, 2H), 7.25–7.16 (m, 5H), 6.97 (d, *J* = 8.4 Hz, 1H), 3.82 (s, 3H), 3.77 (s, 3H), 3.49–3.45 (m, 2H), 2.84 (t, *J* = 7.5 Hz, 2H). HRMS (ESI): calcd for [M+H]^+^ C_21_H_22_NO_4_, 352.1557, found 352.1549.

### 3-Bromo-*N*-(2-phenylethyl)benzamide (**96**)

To a solution of 3-bromobenzoic acid (100 mg, 0.5 mmol), HATU (227 mg, 0.6 mmol), and triethylamine (139 μL, 0.99 mmol) in DMF (5 mL) was added 2-phenylethanamine (69 μL, 0.55 mmol). The reaction mixture was stirred at rt for 16 h, concentrated *in vacuo*, diluted with EtOAc (10 mL), washed with water (5 × 10 mL), passed through a hydrophobic frit, and concentrated *in vacuo*. Purification by flash column chromatography afforded 3-bromo-*N*-(2-phenylethyl)benzamide (113 mg, 75%) as a white solid. ^1^H NMR (400 MHz, DMSO-*d*_6_): δ 8.69 (t, *J* = 5.5 Hz, 1H), 7.98 (t, *J* = 1.8 Hz, 1H), 7.83–7.80 (m, 1H), 7.74–7.71 (m, 1H), 7.43 (t, *J* = 7.9, 1H), 7.32–7.18 (m, 5H), 3.50–3.45 (m, 2H), 2.84 (t, *J* = 7.4 Hz, 2H). LC-MS: *m*/*z* 304/306 [M+H]^+^.

### 3-(3,4-Dimethoxyphenyl)-*N*-(2-phenylethyl)benzamide (**97**)

To a mixture of 3-bromo-*N*-(2-phenylethyl)benzamide (150 mg, 0.49 mmol) and (3,4-dimethoxyphenyl)boronic acid (90 mg, 0.5 mmol) in water (1 mL) and 1,4-dioxane (4 mL) was added K_3_PO_4_ (209 mg, 0.99 mmol), and the reaction mixture was degassed with nitrogen for 10 min. Pd(dtbpf)Cl_2_ (32 mg, 0.05 mmol) was added, and the reaction mixture was degassed with nitrogen for 10 min then heated at 80 °C for 16 h. The reaction mixture was cooled, filtered through Celite, and washed with EtOAc (3 × 5 mL). The combined organics were washed with water (3 × 10 mL), passed through a hydrophobic frit, and concentrated *in vacuo*. Purification by prep-HPLC afforded 3-(3,4-dimethoxyphenyl)-*N*-(2-phenylethyl)benzamide (20 mg, 11%) as an off-white solid. ^1^H NMR (500 MHz, DMSO-*d*_6_): δ 8.03 (t, *J* = 1.6 Hz, 1H), 7.79 (dt, *J* = 7.8, 1.4 Hz, 1H), 7.79 (dt, *J* = 7.8, 1.4 Hz, 1H), 7.75 (dt, *J* = 9.3, 1.3 Hz, 1H), 7.51 (t, *J* = 7.7 Hz, 1H), 7.32–7.24 (m, 6H), 7.22–7.20 (m, 1H), 7.08–7.06 (m, 1H), 3.86 (s, 3H), 3.81 (s, 3H), 3.54–3.50 (m, 2H), 2.87 (t, *J* = 7.5 Hz, 2H). HRMS (ESI): calcd for [M+H]^+^ C_23_H_24_NO_3_, 362.1751, found 362.1767.

### Ethyl (*Z*)-2-Amino-2-(2-(3,4-dimethoxybenzoyl)hydrazono)acetate (**99**)

A mixture of 3,4-dimethoxybenzohydrazide (900 mg, 4.59 mmol, 1 equiv) and ethyl 2-amino-2-thioxo-acetate (610 mg, 4.59 mmol, 1 equiv) was stirred at 180 °C for 1 h. To the mixture was added DMSO (10 mL), and the precipitate was washed with EtOH (5 mL) and concentrated to get the residue. The residue was used to the next step without purification. Ethyl (*Z*)-2-amino-2-(2-(3,4-dimethoxybenzoyl)hydrazono)acetate (400 mg, crude, 29%) was obtained as a white solid. ^1^H NMR (400 MHz, DMSO-*d*_6_): δ 9.94 (br s, 1H), 7.50 (br d, *J* = 6.1 Hz, 1H), 7.43–7.35 (m, 1H), 7.04 (d, *J* = 8.4 Hz, 1H), 6.76 (br s, 2H), 4.26 (q, *J* = 7.1 Hz, 2H), 3.82 6H), 1.29 (t, *J* = 7.0 Hz, 3H).

### Ethyl 3-(3,4-Dimethoxyphenyl)-1*H*-1,2,4-triazole-5-carboxylate (**100**)

To a solution of (*Z*)-2-amino-2-(2-(3,4-dimethoxybenzoyl)hydrazono)acetate (400 mg, 1.35 mmol, 1 equiv) in AcOH (3 mL) was stirred at 100 °C for 1 h. The pH of solution was adjusted to 8 by aq. NaHCO_3_, the aqueous phase was extracted with ethyl acetate (10 mL × 3). The combined organic phase was washed with brine (5 mL), dried with anhydrous Na_2_SO_4_, filtered, and concentrated *in vacuo*. The residue was purified by prep-HPLC (water 0.225%FA–ACN; B%: 13–43%, 10 min). Ethyl 3-(3,4-dimethoxyphenyl)-1*H*-1,2,4-triazole-5-carboxylate (30 mg, 106 μmol, 7%) was obtained as a white solid. LC-MS: *m*/*z* 278 [M+H]^+^.

### *N*-(4-(Azetidine-1-carbonyl)phenethyl)-3-(3,4-dimethoxyphenyl)-1*H*-1,2,4-triazole-5-carboxamide (**101**)

To a solution of ethyl 3-(3,4-dimethoxyphenyl)-1*H*-1,2,4-triazole-5-carboxylate (120 mg, 432 μmol, 1 equiv) and (4-(2-aminoethyl)phenyl)(azetidin-1-yl)methanone (106 mg, 519 μmol, 16 μL, 1.2 equiv) in MeOH (1 mL) was added triethylamine (131 mg, 1.30 mmol, 180 μL, 3 equiv). The mixture was stirred at 60 °C for 12 h and then concentrated to get the residue. The residue was purified by prep-HPLC (water 0.05% ammonia hydroxide v/v–ACN; B%: 5–29%, 10 min). *N*-(4-(Azetidine-1-carbonyl)phenethyl)-3-(3,4-dimethoxyphenyl)-1*H*-1,2,4-triazole-5-carboxamide (12 mg, 27.01 μmol, 6%) was obtained as a yellow solid. ^1^H NMR (400 MHz, MeOD): δ 7.65–7.55 (m, 4H), 7.38 (d, *J* = 8.2 Hz, 2H), 7.05 (d, *J* = 8.3 Hz, 1H), 4.35 (br t, *J* = 7.6 Hz, 2H), 4.17 (br t, *J* = 7.8 Hz, 2H), 3.90 (d, *J* = 10.0 Hz, 6H), 3.66 (t, *J* = 7.3 Hz, 2H), 2.99 (t, *J* = 7.2 Hz, 2H), 2.34 (quin, *J* = 7.8 Hz,2H). HRMS (ESI): calcd for [M+H]^+^ C_23_H_26_N_5_O_4_, 436.1979, found 436.1976.

### Ethyl 2-(2-(3,4-Dimethoxybenzoyl)hydrazinyl)-2-oxoacetate (**102**)

To a solution of 3,4-dimethoxybenzohydrazide (590 mg, 3.01 mmol, 1.00 equiv) and triethylamine (609 mg, 6.01 mmol, 837 μL, 2.00 equiv) in DCM (6.00 mL) was added ethyl 2-chloro-2-oxoacetate (411 mg, 3.01 mmol, 337 μL, 1.00 equiv) dropwise at 0 °C. The mixture was stirred at 25 °C for 16 h, concentrated *in vacuo*, and purified by column chromatography (SiO_2_, PE:EtOAc = 1:1 to 1:4), and the eluant was concentrated. Ethyl 2-(2-(3,4-dimethoxybenzoyl)hydrazinyl)-2-oxoacetate was obtained as a white solid. (570 mg, 1.92 mmol, 63%) ^1^H NMR (400 MHz, CDCl_3_): δ 9.84 (br s, 1H), 9.05 (br s, 1H), 7.49–7.33 (m, 2H), 6.90 (d, *J* = 8.4 Hz, 1H), 4.43 (q, *J* = 7.2 Hz, 2H), 3.94 (d, *J* = 6.0 Hz, 6H), 1.43 (t, *J* = 7.2 Hz, 3H).

### Ethyl 5-(3,4-Dimethoxyphenyl)- 1,3,4-oxadiazole-2-carboxylate (**103**)

To a solution of ethyl 2-(2-(3,4-dimethoxybenzoyl)hydrazinyl)-2-oxoacetate (570 mg, 1.92 mmol, 1.00 equiv) and triethylamine (195 mg, 1.92 mmol, 268 μL, 1.00 equiv) in DCM (6.00 mL) was added a solution of pTsOH (336 mg, 1.92 mmol, 1.00 equiv) in DCM (1.00 mL) at 0 °C. The mixture was stirred at 25 °C for 16 h and then concentrated to 2 mL. The crude product was purified by column chromatography (SiO_2_, PE:EtOAc = 1:0 to 2:1), and the eluant was concentrated. Ethyl 5-(3,4-dimethoxyphenyl)-1,3,4-oxadiazole-2-carboxylate was obtained as a white solid (240 mg, 862 μmol, 44%). ^1^H NMR (400 MHz, CDCl_3_): δ 7.77 (dd, *J* = 2.0, 8.4 Hz, 1H), 7.66 (d, *J* = 2.0 Hz, 1H), 7.00 (d, *J* = 8.4 Hz, 1H), 4.56 (q, *J* = 7.2 Hz, 2H), 3.99 (d, *J* = 5.6 Hz, 6H), 1.50 (t, *J* = 7.2 Hz, 3H). LC-MS: *m*/*z* 279 [M+H]^+^.

### 5-(3,4-Dimethoxyphenyl)-*N*-(4-(3-methoxyazetidine-1-carbonyl)phenethyl)-1,3,4-oxadiazole-2-carboxamide (**104**)

A solution of ethyl 5-(3,4-dimethoxyphenyl)-1,3,4-oxadiazole-2-carboxylate (240 mg, 863 μmol, 1.00 equiv), (4-(2-aminoethyl)phenyl)(3-methoxyazetidin-1-yl)methanone (242 mg, 896 μmol, 1.04 equiv, HCl), and triethylamine (262 mg, 2.59 mmol, 360 μL, 3.00 equiv) in MeOH (4.00 mL) was stirred at 60 °C for 16 h. The crude product was purified by prep-HPLC (water 0.05% ammonia hydroxide v/v)–ACN;B%: 25–45%,10 min), and the desired eluant was concentrated to remove ACN and lyophilized. 5-(3,4-Dimethoxyphenyl)-*N*-(4-(3-methoxyazetidine-1-carbonyl)phenethyl)-1,3,4-oxadiazole-2-carboxamide was obtained as a brown solid. (74 mg, 158 μmol, 18%). ^1^H NMR (400 MHz, DMSO): δ 9.43 (t, *J* = 5.8 Hz, 1H), 7.67 (dd, *J* = 2.1, 8.4 Hz, 1H), 7.60–7.53 (m, 3H), 7.36–7.32 (m, 2H), 7.22–7.19 (m, 1H), 4.42 (s, 1H), 4.23–4.19 (m, 2H), 4.13–4.05 (m, 1H), 3.89–3.80 (m, 7H), 3.56 (q, *J* = 6.8 Hz, 2H), 3.21 (s, 3H), 2.97–2.91 (m, 2H). HRMS (ESI) calcd for [M+H]^+^ C_24_H_27_N_4_O_6_, 467.1931, found 467.1935.

Compounds **50**, **106–111** were all commercial compounds.

### *Mtb* Pks13 TE Domain Expression and Purification

The plasmid construct design, protein expression, and purification were carried out as described previously.^17^

### Co-crystallization of *Mtb* Pks13 TE Domain in Complex with Ligands

The *Mtb* Pks13 TE domain crystallization conditions under vapor diffusion setting have previously been determined and optimized using 0.1 M Tris-HCl pH 8.5, 1.8–2.0 M ammonium sulfate, and 2–5% (v/v) of polypropylene glycol P-400 as an additive^17^ to allow back soaking using the benzofuran ligands. However, a direct co-crystallization platform was developed due to the crystals being very fragile and difficult to manipulate between drops. Ligands were first solubilized in 100% DMSO to a final concentration of 100 mM as stock solution. For *Mtb* Pks13 TE domain-ligand co-crystallization experiment, ligand was added to the protein solution (15–20 mg/mL) to a final concentration of 0.5–2 mM keeping the final DMSO concentration at <5%, mixed with a gentle stirring using a pipet tip, and incubated on ice for approximately 2 h. One μL of protein–ligand solution was mixed with 1 μL of reservoir solution (0.1 M Tris-HCl pH 7–8.5, 1.6–1.85 M ammonium sulfate, 0–3% polypropylene glycol P-400), and hanging drops were set at 20 °C. The co-crystals (hexagonal plate-clusters) grew within a week, reached maximum size within 2 weeks, and did not require back soaking with ligands as previously determined.^17^

### X-ray Data Collection and Processing

For X-ray diffraction data collection, the *Mtb* Pks13 TE ligand co-crystals were cryo-protected using Fomblin (Sigma) and flash frozen in liquid nitrogen. X-ray diffraction data were collected at the Diamond Light Source (DLS), Macromolecular Crystallography (MX) beamlines I04-1 and I24 at 100 K. The Mtb Pks13 TE-**50** and Mtb Pks13 TE-**14** data was integrated with XDS^30^ and scaled using Aimless^31^ as part of the Xia2 DIALS auto processing pipeline at DLS, while the Mtb Pks13 TE-**33** data was integrated and scaled using autoPROC.^32^ Data processing statistics are given in Table S1.

### Determination of *Mtb* Pks13 TE-Ligand Co-crystal Structures and Model Refinement

The co-crystal structures of *Mtb* Pks13 TE domain in complex with the ligands were solved by molecular replacement using the crystal structure of the apo form of *Mtb* Pks13 TE domain (PDB 5V3W(17)) as a search model in Phaser MR.^33^ Phenix^34^ and Refmac^35^ were used for iterative rounds of refinement with model building carried out in COOT.^36^ The figures were made using PyMOL (Schrödinger, LLC). PDB ID codes: Structure factors and atomic coordinates have been deposited with the RCSB Protein Data Bank with the codes 8Q0T, 8Q0U, and 8Q17. Files may be retrieved online at http://www.rcsb.org/pdb/home/home.do

### Pks13 Thioesterase Domain *In Vitro* Enzyme Assays

these were performed as described previously.^17^ In short, IC_50_ determinations were tested in 10-point concentration curves using a 384 well assay format with compounds predispensed using an Echo 550 (Labcyte). Final assay conditions were 50 mM Tris pH 7.0, 100 mM NaCl, 0.1 mM TCEP, 0.5 μM Pks13 TE domain. The reaction was initiated by the addition of 4-methylumbelliferyl heptanoate substrate (Sigma; 10 mM stocks in DMSO) to a final assay concentration of 25 μM. Fluorescence (Ex. 350 nm; Em. 450 nm) was measured after 180 min incubation at rt using a PheraStar2 plate reader, and data was analyzed using ActivityBase XE.

### *M. tuberculosis* H37Rv MIC Determination

All methods used for both extra- and intracellular MIC determinations have been described previously.^37^

### MIC Analyses

MIC analyses for H37Rv and pks13-TetON strains were performed as described previously.^18^

### Intrinsic Clearance (Cli), Aqueous Solubility, and Parallel Artificial Membrane Permeability (PAMPA) Experiments

*In vitro* ADME experiments were performed exactly as reported.^38−40^

### CHI LogD_pH7.4_ Measurement

Test compounds were prepared as 0.5 mM solutions in 50:50 acetonitrile:water and analyzed by reversed-phase HPLC-UV (wavelength 254 nm) using a Phenomenex Luna C18 100 Å 150 × 4.6 mm 5 μm column with a gradient of aqueous phase (50 mM ammonium acetate, pH 7.4) and mobile phase (acetonitrile) as described.^41,42^

### Murine Pharmacokinetics

Murine pharmacokinetic studies were performed exactly as reported.^40^ All regulated procedures, at the University of Dundee, on living animals was carried out under the authority of a project license issued by the Home Office under the Animals (Scientific Procedures) Act 1986, as amended in 2012 (and in compliance with EU Directive EU/2010/63). License applications have been approved by the University’s Ethical Review Committee (ERC) before submission to the Home Office. The ERC has a general remit to develop and oversee policy on all aspects of the use of animals on university premises and is a subcommittee of the University Court, its highest governing body.

## Data Availability

PDB codes for Pks13 TE with bound compounds **50**, **33**, and **14** are 8Q0T, 8Q0U, and 8Q17, respectively.

## Supporting Information Available

Table S1, X-ray data collection and refinement statistics for Pks13 TE-compound complexes; Figure S1, molecular interactions for compounds **50**, **14**, and **33**; Figure S2, Pks13 hypomorph strain growth profile for compounds **50**, **14**, **15**, and **33**; and representative *in vivo* and *in vitro* compound HPLC UV traces (PDF)
Manuscript key in *vitro* data table with PAINS alert analysis and SMILES (CSV)

## Abbreviations Used

AcOH: acetic acid
ACN, MeCN: acetonitrile
ATc: anhydrotetracycline
CHI: chromatographic hydrophobicity index
Cli: intrinsic clearance
DIPEA: *N,N*-diisopropylethylamine
EDCI: 1-(3-(dimethylamino)propyl)-3-ethylcarbodiimide hydrochloride
EtOAc: ethyl acetate
EtOH: ethanol
Et_3_N: triethylamine
HATU: (1-[bis(dimethylamino)methylene]-1*H*-1,2,3-triazolo[4,5-*b*]pyridinium-3-oxide hexafluorophosphate
HOBt: hydroxybenzotriazole
LogD: logarithm of distribution coefficient
MeOH: methanol
MeOD: deuterated methanol
mg: milligrams
mmol: millimoles
μmol: micromoles
μL: microlitres
Mtb: *Mycobacterium tuberculosis*
ND: not determined
PE: petroleum ether
Pks13: polyketide synthase 13
pTsOH: *p*-toluenesulfonic acid
T3P: propylphosphonic anhydride
TE: thioesterase
TCEP: tris(2-carboxyethyl)phosphine
