# Supplementary material for: Identification and Optimization of Novel Inhibitors of the Polyketide Synthase 13 Thioesterase Domain with Antitubercular Activity
Source: J Med Chem. 2023 Nov 10;66(22):15380–408. doi: 10.1021/acs.jmedchem.3c01514 (PMC10683028; doi:10.1021/acs.jmedchem.3c01514)
Supplement: Supplementary file 1 — jm3c01514_si_001.pdf [file jm3c01514_si_001.pdf]

Supporting Information

Identification and optimisation of novel inhibitors of the Polyketide synthase 13 thioesterase domain with antitubercular activity

Simon R. Green[‡*], Caroline Wilson[‡], Thomas C. Eadsforth[‡], Avinash S. Punekar[‡], Fabio K. Tamaki[‡], Gavin Wood[‡], Nicola Caldwell[‡], Barbara Forte[‡], Neil R. Norcross[‡], Michael Kiczun[‡], John M. Post[‡], Eva Maria Lopez-Román[ᵩ], Curtis A. Engelhart[¥], Iva Lukac[‡], Fabio Zuccotto[‡], Ola Epemolu[‡], Helena I. M. Boshoff[ᵻ], Dirk Schnappinger[¥], Chris Walpole[≠], Ian H. Gilbert[‡], Kevin D. Read[‡], Paul G. Wyatt[‡] and Beatriz Baragaña[‡*]

[‡]Drug Discovery Unit, Division of Biological Chemistry and Drug Discovery, School of Life Sciences, University of Dundee, Dundee, DD1 5EH, UK

[¥]Department of Microbiology and Immunology, Weill Cornell Medical College, New York, NY, 10065, USA

[ᵩ]Global Health Medicines R&D, GlaxoSmithKline, Severo Ochoa 2, Tres Cantos, 28760, Madrid, Spain

[ᵻ]Tuberculosis Research Section, Laboratory of Clinical Immunology and Microbiology, NIAID, NIH, 9000 Rockville Pike, Bethesda, MD, 20892, USA

≠Structural Genomics Consortium, Research Institute of the McGill University Health Centre, 1001 Boulevard Décarie, Site Glen Block E, ES1.1614, Montréal, QC H4A 3J1, Canada

***Corresponding Author Information:**
S.R.Green@dundee.ac.uk
b.baragana@dundee.ac.uk

Table of Contents

Title Page S1
Table S1: Crystal data collection and refinement statistics. S2
Supplemental Figure 1: Pks13 binding pocket overlays for compounds **50**, **14** & **33**. S3
Supplemental Figure 2: Dose response curves for compounds **50**, **14**, **15** & **33** tested against a tet-regulated Pks13 strain S3
Representative *in vivo* and *in vitro* compound HPLC UV traces: S4

Table S1: Crystal data collection and refinement statistics.

	**Pks13-TE:50**	**Pks13-TE:33**	**Pks13-TE:14**
**PDB ID**	8Q0T	8Q0U	8Q17
**Data Collection**			
Space group	$P2_12_12$	$P2_12_12$	$P2_12_12$
Cell dimensions			
a, b, c (Å)	88.3, 110.2, 57.9	87.6, 108.6, 57.5	88.4, 109.8, 57.6
α, β, γ (°)	90, 90, 90	90, 90, 90	90, 90, 90
Resolution (Å)	88.27-1.80 (1.84-1.80)	68.17 - 1.8 (1.84 - 1.8)	84.51 - 1.71 (1.77 - 1.71)
Total reflections	364265 (22465)	344236 (18163)	112784 (6923)
Unique reflections	51471 (3078)	48490 (3026)	56541 (3520)
Redundancy	7.1 (7.3)	7.1 (6.0)	11.6 (7.5)
Completeness (%)	97.1 (99.9)	93.8 (100)	92.0 (54.5)
$I/\sigma I$	12.6 (1.6)	4.1 (1.3)	12.6 (1.1)
Wilson B-factor	23.3	21.42	24.131
R_{merge}	0.096 (1.109)	0.301 (1.128)	0.112 (1.991)
R_{meas}	0.11 (1.281)	0.349 (1.352)	0.118 (2.140)
$CC_{½}$	0.999 (0.625)	0.93 (0.540)	0.995 (0.764)
**Refinement**			
Resolution (Å)	1.80	1.80	1.71
No. of reflections	48824 (3627)	45754 (3565)	55847 (3500)
No. of reflections as R_{free}	2565 (202)	2430 (195)	2724 (195)
R_{work} / R_{free} (%)	18.6 / 22.4	18.9 / 23.5	19.53 / 23.87
No. of non-hydrogen atoms			
Protein	4298	4323	4351
Ligand	60	68	118
Water	301	444	340
B-factors (Å^2)			
Average	32.106	30.259	34.15
Protein	32.05	29.69	33.40
Ligand	26.81	43.44	45.41
Water	34.09	39.17	38.29
R.M.S. deviations			
Bond lengths (Å)	0.0183	0.0179	0.0196
Bond angles (°)	1.80	1.86	1.97
Clashscore	N/A	5.14	2.98
Ramachandran Plot			
Favoured (%)	98.33	97.94	97.8
Allowed (%)	1.48	1.87	2.0
Outliers (%)	0.19	0.19	0.19

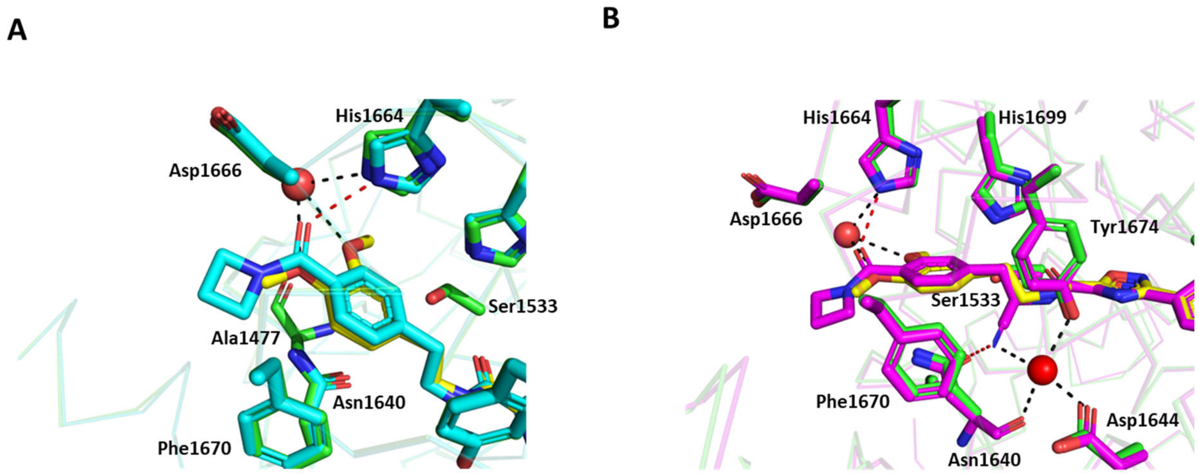

Supplemental Figure 1: Pks13 binding pocket overlays for compounds **50**, **14** & **33**.

Superimposition of the crystal structures of Pks13-**50** (PDB ID 8Q0T: yellow and green) and **14** (PDB ID 8Q17: cyan) showing the carbonyl oxygen of the azetidine making an extra direct hydrogen bond interaction (dashed red line) between the side chain of His1664, replacing a water molecule previously bound in the **50** structure (**A**). A similar binding mode is seen in the crystal structure comparison of Pks13-**33** (PDB ID 8Q0U: purple), with an additional hydrogen bond formed between the cyano group and side chain of Asn1640 (**B**).

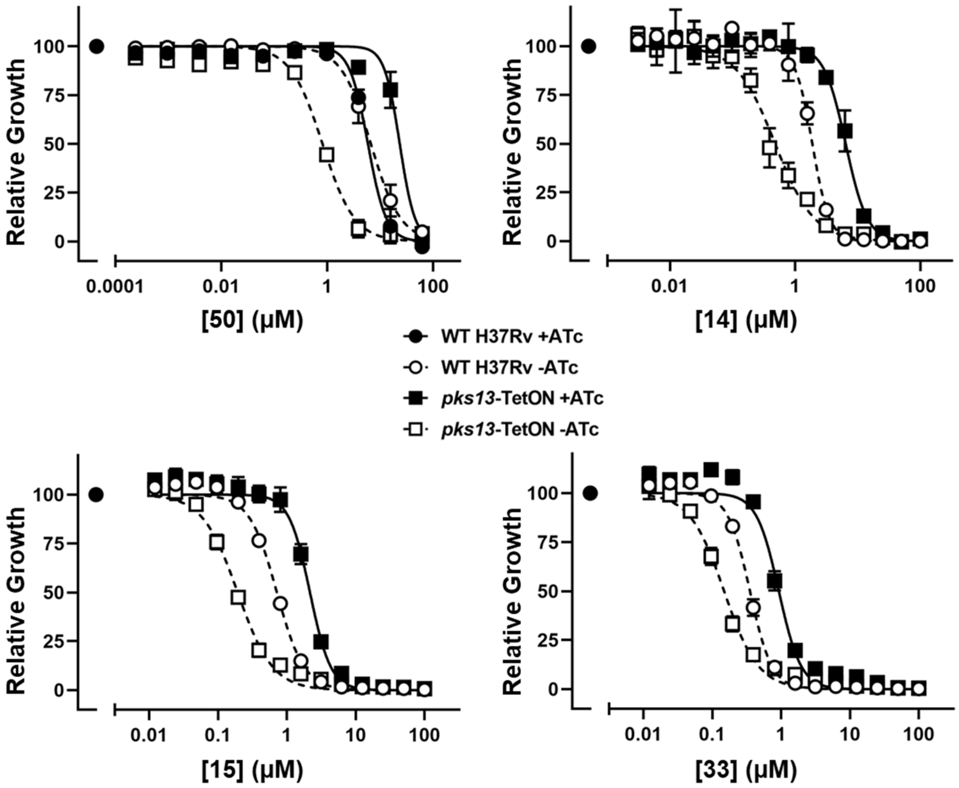

Supplemental Figure 2: Dose response curves for compounds **50**, **14**, **15** & **33** tested against a tet-regulated Pks13 strain

Growth in the presence of anhydrotetracycline (ATc) results in modest transcriptional overexpression of *pks13* while removal of ATc results in transcriptional repression of *pks13* expression. Growth in the presence of compounds is shown relative to DMSO treated samples. Data are representative of two independent experiments.

HPLC Traces

In-vivo compounds

15

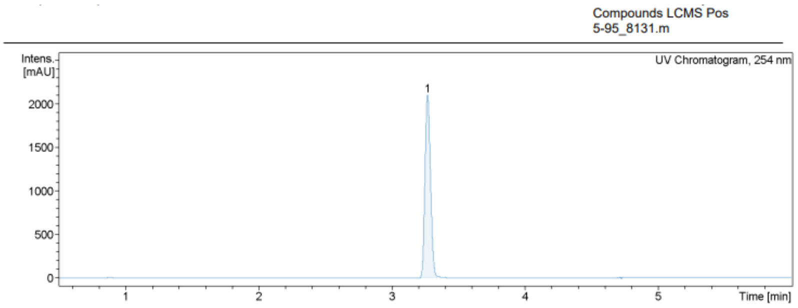

#	RT [min]	Area Frac. %	Chromatogram
1	3.3	100.00	UV Chromatogram, 254 nm
2	3.3	91.70	BPC 75.0000-1601.0000 +, Masses excluded
3	5.3	8.30	BPC 75.0000-1601.0000 +, Masses excluded

44

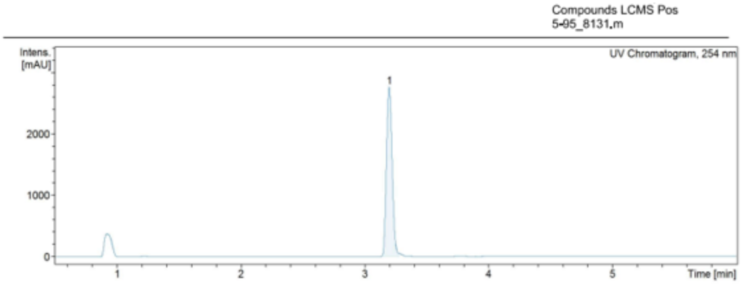

#	RT [min]	Area Frac. %	Chromatogram
1	3.2	100.00	UV Chromatogram, 254 nm
2	3.2	96.94	BPC 75.0000-1601.0000 +, Masses excluded
3	5.3	3.06	BPC 75.0000-1601.0000 +, Masses excluded

80

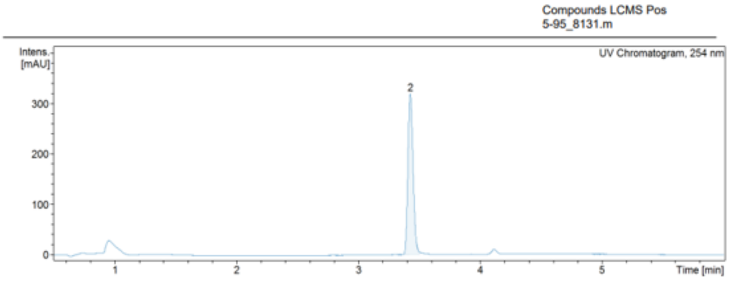

#	RT [min]	Area Frac. %	Chromatogram
1	3.0	1.67	BPC 74.0000-1600.0000 +, Masses excluded
2	3.4	100.00	UV Chromatogram, 254 nm
3	3.5	72.11	BPC 74.0000-1600.0000 +, Masses excluded
4	4.2	18.66	BPC 74.0000-1600.0000 +, Masses excluded
5	4.6	1.58	BPC 74.0000-1600.0000 +, Masses excluded
6	5.1	2.78	BPC 74.0000-1600.0000 +, Masses excluded
7	5.4	3.20	BPC 74.0000-1600.0000 +, Masses excluded

Representative In-vitro compounds

16

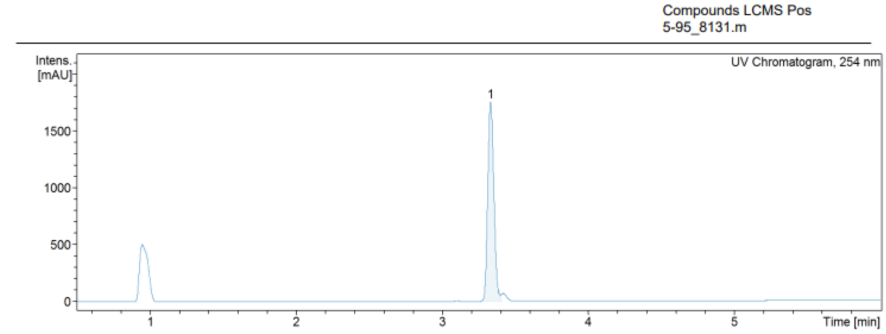

#	RT [min]	Area Frac. %	Chromatogram
1	3.3	100.00	UV Chromatogram, 254 nm
2	3.4	89.13	BPC 74.0000-1601.0000 +, Masses excluded
3	4.3	3.12	BPC 74.0000-1601.0000 +, Masses excluded
4	4.4	5.46	BPC 74.0000-1601.0000 +, Masses excluded
5	5.3	2.29	BPC 74.0000-1601.0000 +, Masses excluded

17

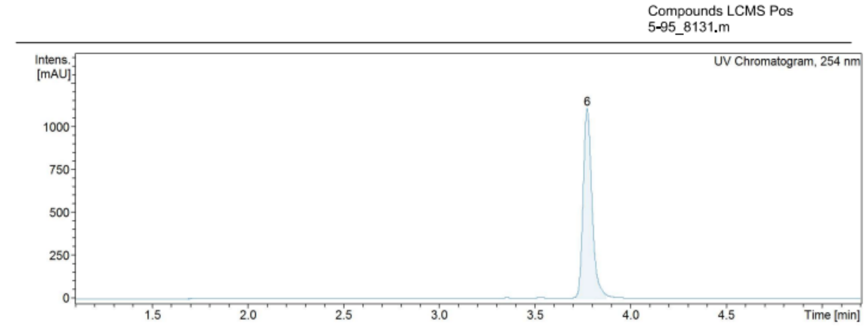

#	RT [min]	Area Frac. %	Chromatogram
1	1.1	1.90	BPC 74.0000-1201.0000 +, Masses excluded
2	1.2	4.84	BPC 74.0000-1201.0000 +, Masses excluded
3	1.3	3.43	BPC 74.0000-1201.0000 +, Masses excluded
4	1.5	7.68	BPC 74.0000-1201.0000 +, Masses excluded
5	3.4	2.90	BPC 74.0000-1201.0000 +, Masses excluded
6	3.8	100.00	UV Chromatogram, 254 nm
7	3.8	79.24	BPC 74.0000-1201.0000 +, Masses excluded

101

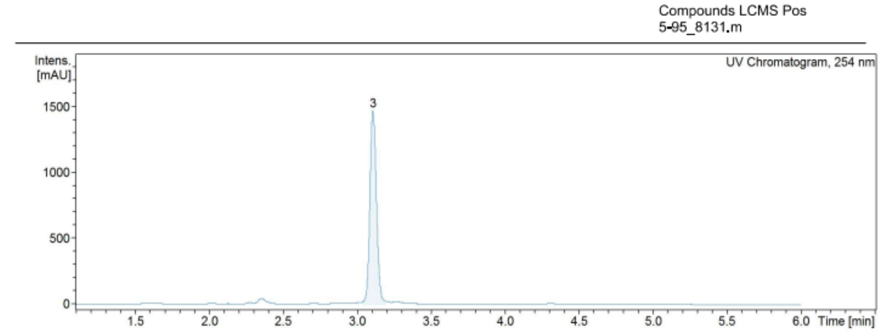

#	RT [min]	Area Frac. %	Chromatogram
1	2.4	9.25	BPC 75.0000-1201.0000 +, Masses excluded
2	2.4	3.69	BPC 75.0000-1201.0000 +, Masses excluded
3	3.1	100.00	UV Chromatogram, 254 nm
4	3.1	87.06	BPC 75.0000-1201.0000 +, Masses excluded

61

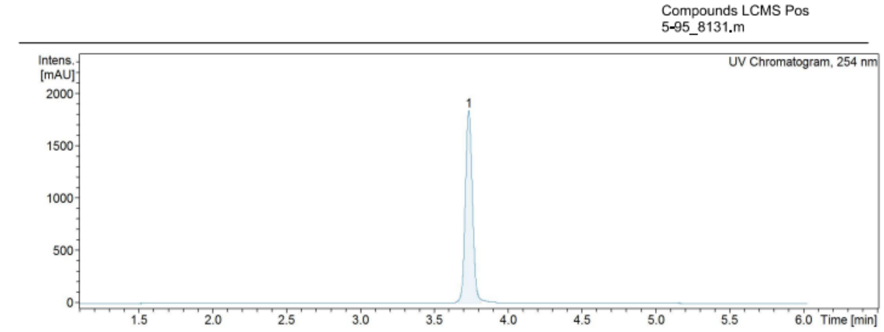

#	RT [min]	Area Frac. %	Chromatogram
1	3.7	100.00	UV Chromatogram, 254 nm
2	3.8	100.00	BPC 74.0000-1201.0000 +, Masses excluded

62

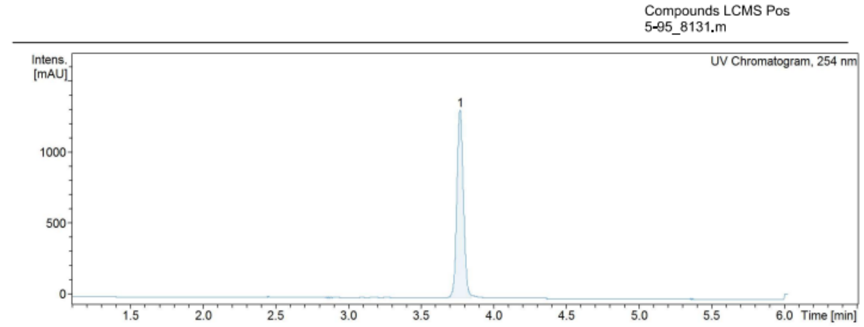

#	RT [min]	Area Frac. %	Chromatogram
1	3.8	100.00	UV Chromatogram, 254 nm
2	3.8	100.00	BPC 74.0000-1200.0000 +, Masses excluded

63

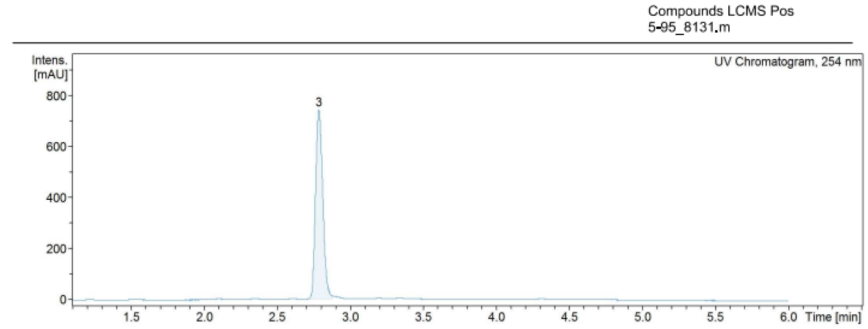

#	RT [min]	Area Frac. %	Chromatogram
1	1.2	4.63	BPC 74.0000-1201.0000 +, Masses excluded
2	1.4	3.42	BPC 74.0000-1201.0000 +, Masses excluded
3	2.8	100.00	UV Chromatogram, 254 nm
4	2.8	89.71	BPC 74.0000-1201.0000 +, Masses excluded
5	3.3	2.24	BPC 74.0000-1201.0000 +, Masses excluded

64

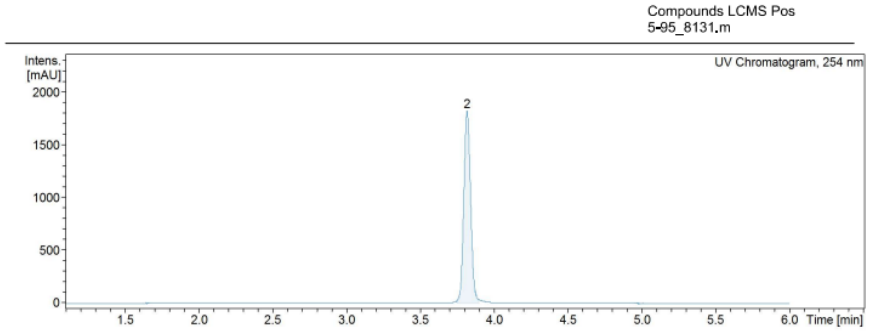

#	RT [min]	Area Frac. %	Chromatogram
1	1.2	2.29	BPC 74.0000-1201.0000 +, Masses excluded
2	3.8	100.00	UV Chromatogram, 254 nm
[illegible]	[illegible]	[illegible]	[illegible]

65

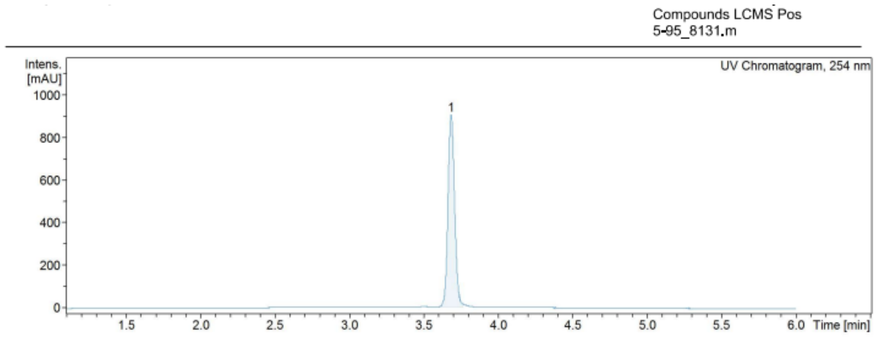

#	RT [min]	Area Frac. %	Chromatogram
1	3.7	100.00	UV Chromatogram, 254 nm
2	3.7	100.00	BPC 74.0000-1201.0000 +, Masses excluded

66

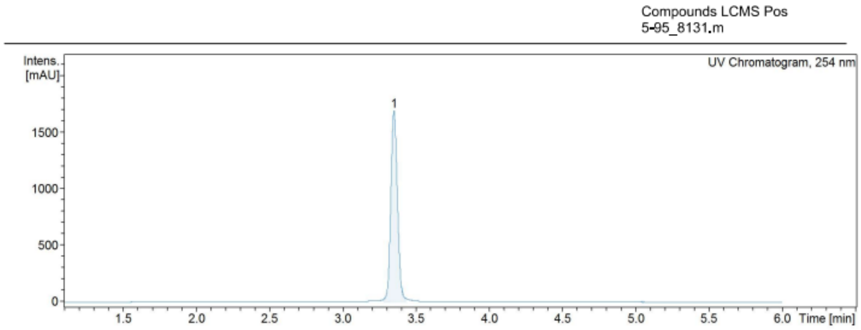

#	RT [min]	Area Frac. %	Chromatogram
1	3.4	100.00	UV Chromatogram, 254 nm
2	3.4	100.00	BPC 74.0000-1201.0000 +, Masses excluded

67

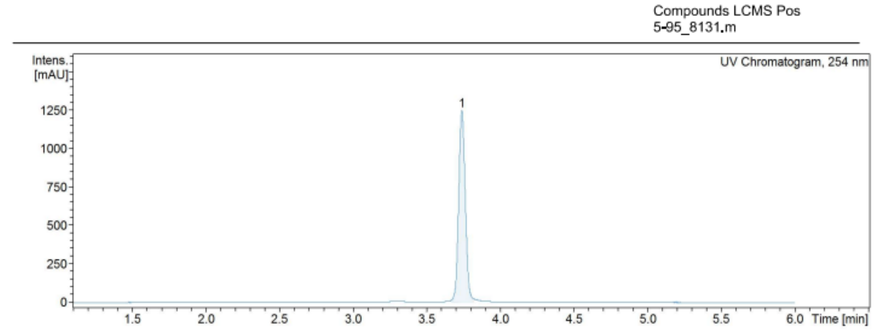

#	RT [min]	Area Frac. %	Chromatogram
1	3.7	100.00	UV Chromatogram, 254 nm
2	3.8	100.00	BPC 74.0000-1201.0000 +, Masses excluded

3

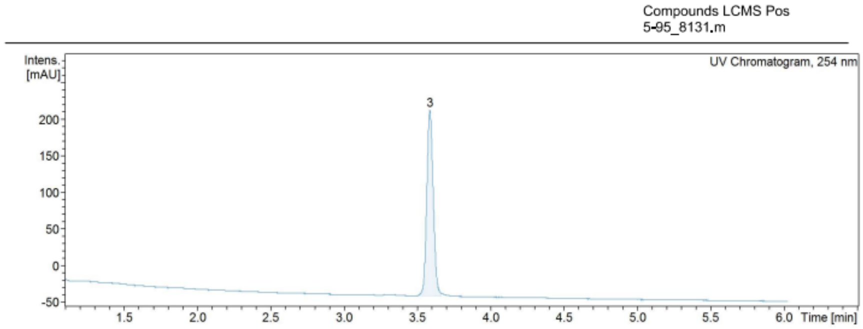

#	RT [min]	Area Frac. %	Chromatogram
1	1.1	3.44	BPC 75.0000-1201.0000 +, Masses excluded
2	1.1	7.19	BPC 75.0000-1201.0000 +, Masses excluded
3	3.6	100.00	UV Chromatogram, 254 nm
4	3.6	89.37	BPC 75.0000-1201.0000 +, Masses excluded

45

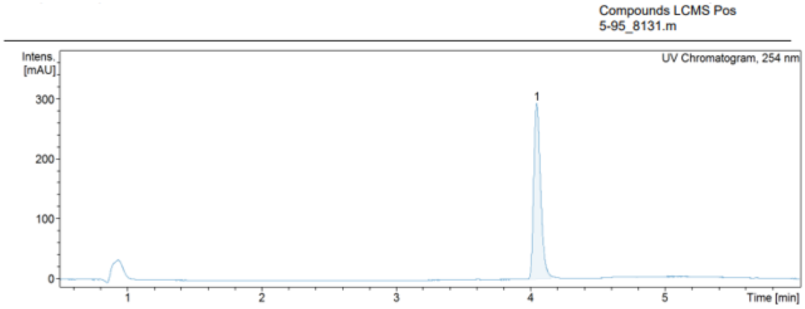

#	RT [min]	Area Frac. %	Chromatogram
1	4.0	100.00	UV Chromatogram, 254 nm
2	4.1	95.60	BPC 74.0000-1601.0000 +, Masses excluded
3	5.0	4.40	BPC 74.0000-1601.0000 +, Masses excluded
